# Pd‐Catalyzed Allylation of Imines to Access α‐CF_3_‐Substituted α‐Amino Acid Derivatives

**DOI:** 10.1002/ejoc.201901272

**Published:** 2019-11-06

**Authors:** Michael Winter, Hyunwoo Kim, Mario Waser

**Affiliations:** ^1^ Institute of Organic Chemistry Johannes Kepler University Linz Altenbergerstr. 69 4040 Linz Austria; ^2^ Department of Chemistry Korea Advanced Institute of Science and Technology 291 Daehak‐ro 34141 Daejeon Yuseong‐gu Republic of Korea

**Keywords:** Amino acids, Allylation, Umpolung, Asymmetric catalysis, Trifluoropyruvate

## Abstract

We herein report a high yielding protocol for the direct α‐allylation of easily accessible trifluoropyruvate‐derived imines using Pd‐catalysis. The reaction gives access to a variety of different α‐allylated‐α‐CF_3_‐amino acids in a straightforward manner, starting from commercially available trifluoropyruvate. We also provide a proof‐of‐concept for an enantioselective protocol (up to *er* = 75:25) by using chiral phosphane ligands.

## Graphical abstract

The Pd‐catalyzed allylation of trifluoropyruvate‐based imines gives access to different α‐allylated‐α‐CF_3_‐substituted‐α‐amino acids straightforwardly in good to excellent yields.

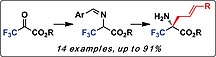

## Introduction

Syntheses and applications of fluorine‐containing α‐amino acids (α‐AA) are heavily investigated research fields and the incorporation of such amino acids in peptides or proteins is an appealing strategy to alter their (bio)‐chemical and (bio)‐physical properties^1^, ^2^, ^3^ One particularly powerful approach to influence the nature of biologically active molecules is the introduction of a trifluoromethyl group.^4^ Not surprisingly, α‐CF_3_‐containing α‐amino acids (α‐CF_3_‐α‐AA) have thus emerged as target molecules of high interest.^2^

**Scheme 1:**
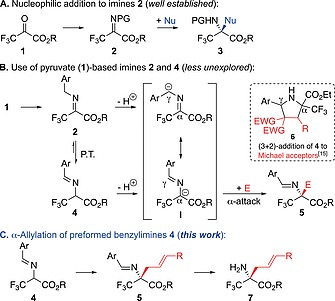
Known and targeted utilization of trifluoropyruvate‐derived imines to access chiral α‐CF_3_‐α‐amino acids.

A broad variety of complementary strategies for the synthesis of (chiral) CF_3_‐containing compounds are known,^5^ either relying on the nucleophilic^6^ or electrophilic^7^ (late‐stage) CF_3_‐introduction on already appropriately substituted compounds, or making use of simple CF_3_‐containing (commercial) building blocks to access further structural complexity. With respect to the synthesis of quaternary α‐CF_3_‐α‐AA one common methodology is to make use of simple CF_3_‐ketimines and install the α‐AA motive by means of Strecker‐type chemistry.^8^ An alternative approach relies on the use of commercially available 3,3,3‐trifluoropyruvates **1**, which are commonly used building blocks to access quaternary α‐CF_3_‐esters by means of nucleophilic additions either to the pyruvates themselves,^9^ or by using the corresponding ketimines **2**, which upon addition of different nucleophiles give diversely substituted quaternary α‐CF_3_‐α‐amino acid derivatives **3** (Scheme 1A).^10^

In addition to using imines as acceptors for nucleophilic additions, the last years saw an increasing number of reports demonstrating that the inherent reactivity of imines can be inverted (making the imine carbon nucleophilic). Such an imine umpolung can be achieved by using a suited benzylamine protecting group that allows for the formation of ambident nucleophilic azaallyl anions under basic reaction conditions, which then preferably react with electrophiles in the α‐position (compare with Scheme 1B).^11^, ^12^, ^13^, ^14^, ^15^, ^16^ Accordingly, these umpolung approaches allow for the synthesis of valuable acyclic^12^, ^13^ or cyclic^14^, ^15^ products by starting from easily accessible imines. Interestingly however, CF_3_‐containing benzylic imines **2** have only sparingly been reported and utilized as building blocks so far.^15^, ^17^ This comes as a surprise, as the reaction between benzylamines and pyruvates **1** proceeds easily and already in the initial reports by Soloshonok and co‐workers the very rapid tautomerization of the initially formed imines **2** to imines **4** was observed.^17^ This fast isomerization allows for the direct formation of **4** by heating **1** with benzylamines and most likely proceeds via formation of the intermediate azaallyl anion **I**. Given this observation, it seemed very likely to us that reactions of both, imines **2** (provided that these can be isolated) and the thermodynamically more stable imines **4** with suited electrophiles under basic conditions would proceed via α‐attack predominantly. Overall such this concept would therefore result in a formal reactivity umpolung of the commercially available simple starting materials **1** and will give access to a variety of (chiral) α‐CF_3_‐α‐amino acid derivatives in a direct and, compared to established protocols, complementary manner (Scheme 1B).

We have recently shown that preformed imines **4** undergo highly diastereoselective (3+2)‐type cyclizations with Michael acceptors to access CF_3_‐proline derivatives **6**, with the α‐position acting as the donor site.^15^ Based on these observations, we now became interested in developing this concept further towards a more general approach to access (novel) acyclic α‐CF_3_‐α‐amino acid derivatives in an unprecedented fashion. We opted for Pd‐catalyzed α‐allylation approaches,^18^ as these would give access to highly functionalized α‐allylated trifluoroalanine derivatives **5** (and upon imine hydrolysis **7**) straightforwardly (Scheme 1C).^19^, ^20^

## Results and Discussion

**Scheme 2:**
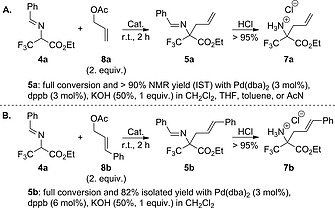
Optimized reaction conditions for the racemic α‐allylation of **4a** with allylic acetates **8a** and **8b** (dba = dibenzylideneacetone; dppb = 1,4‐bis(diphenylphosphanyl)butane: IST = internal standard).

We started our investigations by carrying out the racemic reactions between the ethyl ester **4a** and the simple allylic acetates **8a** and **8b** (Scheme 2). Literally the first attempt with **4a** and **8a** in the presence of bis(dibenzylideneacetone)palladium (Pd(dba)_2_) as a simple and cheap Pd(0)‐source and 1,4‐bis(diphenylphosphanyl)butane (dppb) resulted in the almost quantitative formation of **5a** (using aqueous KOH as a base in acetonitrile as the solvent). The reaction was then found to be rather tolerant to different solvents and CH_2_Cl_2_, toluene and THF were equally well suited (with NMR yields > 90 % in all cases after 1–2 h). Other aqueous alkali hydroxide bases were well tolerated too, and a catalyst loading of 3 mol‐% Pd(dba)_2_ and 3 mol‐% dppb was found to be the optimum (lower amounts unfortunately did not allow for full conversions even after longer reaction times). Unfortunately, however, product **5a** hydrolyzed relatively quickly during normal phase silica gel column chromatography and could therefore only be isolated in around 35 % yield after column chromatography. Thus, crude **5a** was directly hydrolyzed in a quantitative manner by treatment with either HCl or TFA. This procedure also allowed for a simple extractive purification, giving isolated **7a** in around 85 % isolated yield (over both steps) on up to 1 mmol scale (Scheme 2A).

**Scheme 3:**
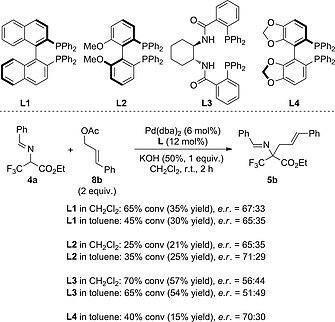
Attempted asymmetric α‐allylation of **4a** with cinnamyl acetate **8b** (*er* determined by HPLC analysis using a chiral stationary phase).

Having identified operationally simple and high yielding conditions for the formation of the α‐allylated amino acid derivatives **5a** and **7a**, we next tested the reaction of **4a** with the cinnamyl acetate **8b** (Scheme 2B). Interestingly, this transformation was found to be a bit more sensitive to the used solvent and incomplete conversion was observed in acetonitrile, while CH_2_Cl_2_ and toluene allowed for complete conversion to **5b** within 2 h again (other Pd‐sources did not perform better in CH_3_CN). Concerning the catalyst loading again 3 mol‐% of (Pd(dba)_2_) were found to be the optimum. Surprisingly in this case however the use of 6 mol‐% of the ligand (dppb) were necessary and other (i.e. chiral) ligands like e.g. BINAP were giving lower yields (please see Scheme 3 below for our attempts to develop an asymmetric variant). In contrast to **5a**, compound **5b** was found to be more stable under column chromatography conditions and could be isolated in 82 % isolated yield. The reaction turned out to be robust and could easily be carried out using 1 g of **4a** (3.85 mmol) giving **5b** in 77 % isolated yield. Hydrolysis with HCl then gave the ammonium chloride **7b** quantitatively. The later compound could also be successfully employed for standard amide bond forming reactions with benzoyl chloride or N‐protected glycine under classical peptide coupling conditions, as outlined in the online supporting information.^21^

With these robust racemic conditions in hand, we next evaluated the scope of the reaction between imines **4** and differently substituted γ‐substituted acetates **8** (Table 1). We initially wondered if a change of the imine‐protecting group may influence the outcome, but when replacing the phenyl imine group in **4a** by a p‐NO_2_‐phenyl group the outcome was not much different (entry 1), showing that this group does not significantly influence the reactivity of the nucleophile **4**. A broad variety of different aryl‐based acetates **8b‐l** were well accepted, all resulting in isolated yields between 70–90 % (entries 1–11). Only the thienyl‐based acetate **8m** reacted somewhat slower and the corresponding product **5m** could only be isolated in 54 % yield (entry 12). We also tested the crotyl‐based acetate **8n** (entry 13), but unfortunately conversion was much slower in that case and the product tends to decompose rather quickly. Finally, the branched acetate **8o** was employed as well (entry 14), which resulted again in the formation of the linear allylation product **5b** under the Pd‐catalyzed conditions, but in notably lower yield (and much slower) compared to the analogous reaction with **8b** (compare entries 1 and 14).

**Table 1:**
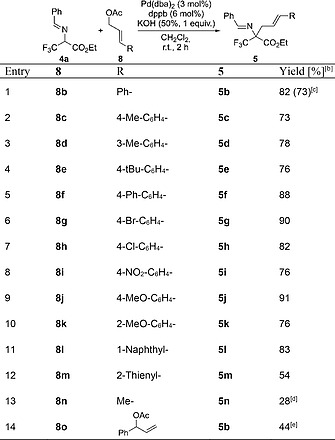
Application scope of the racemic α‐allyation using different γ‐substituted acetates **8** a

a All reactions were run at room temperature using 0.1 mmol **4** and 0.2 mmol **8**.

Isolated Yields.

Using p‐NO_2_‐phenyl imine **4**.

Around 35 % conversion.

Around 50 % conversion.

Having demonstrated the applicability of the racemic protocol, we then put our efforts on developing an asymmetric variant. Here we focused on the use of the four commercially available chiral diphosphane ligands **L1**–**L4** 22 We started by investigating the reaction between imine **4a** and cinnamyl acetate **8b** (Scheme 3). Unfortunately, the chiral ligands slowed down the reaction significantly, compared to the use of the achiral dppb (compare with Scheme 2B), and even with 6 mol‐% Pd(dba)_2_ and 12 mol‐% of the ligands the reaction did not proceed to completion (longer reaction times did not allow to overcome this limitation as the reactions stalled before completion). In addition, in most of these attempts pronounced amounts of unidentified side products were formed, thus rationalizing the considerable difference between conversion and isolated yields. Besides these limitations also the enantioselectivity was found to be modest only (highest *er* = 71:29 obtained with ligand **L2**). A screening of different solvents and dilutions did not improve this outcome. In addition, we also tried other imine protecting groups (like the above‐mentioned p‐NO_2_‐phenyl group) but without any improvement. It has been well described that the synergistic combination of chiral transition metal catalysis and asymmetric ammonium salt ion pairing catalysis can be rather fruitful to facilitate asymmetric allylation reactions.[13c], 23 We therefore also tested the reactions with chiral phosphane ligands and in the presence of different asymmetric ammonium salts (i.e. Cinchona alkaloid‐based ones^24^) but in neither case any positive effect on the enantioselectivity was observed.

**Scheme 4:**
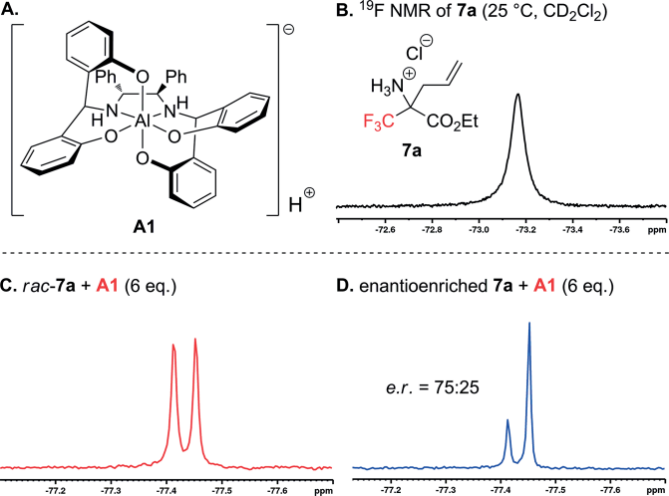
Direct chiral ^19^F NMR analysis of **7a** using the chiral aluminium complex **A1**.

As we found the racemic allylation with allylic acetate **8a** to be somewhat more robust and tolerant to different solvents and lower ligand loading than the one with **8b** (Scheme 2), we also investigated the asymmetric allylation of **4a** with **8a** under a variety of different conditions (Scheme 4). Unfortunately, the quick hydrolysis of **5a** under protic and/or slightly acidic conditions turned out to be challenging for determining the enantioselectivity. With any mobile phase and HPLC column the hydrolysis of **5a** was found to be rather fast, making a reliable chiral HPLC analysis of this compound impossible. On the other hand, the quantitative hydrolysis to **7a** was easily possible, but we were not able to identify any suited HPLC method to directly determine the *er* of compound **7a**. One option to overcome this limitation would be to carry out the derivatization of the free amine with FMOC, which was found to be possible in principle. However, this turned out to be rather time consuming, low yielding, and required a not that easy column chromatographic purification, which made this approach not practical for a fast screening.

Alternatively, the direct use of chiral NMR shift reagents may allow for a simple determination of the enantiomeric composition by just mixing the (crude) reaction product with the shift reagent.^25^ Some of us (H. Kim's group) have recently introduced a novel class of chiral aluminum complexes **A** (Scheme 4A) that allowed for the straightforward analysis of chiral amines using ^1^H NMR spectroscopy.^26^ Considering the simplicity of this approach, we reasoned that this would allow us to overcome the difficulties observed for the analysis of compound **7a** with HPLC by using standard ^19^F NMR to directly quantify the enantiomeric composition of the CF_3_‐amino acid **7a**. We were glad to see that mixing racemic **7a** with 6 equiv. of the aluminum complex **A1** leads to 1:1 splitting of the CF_3_‐signal in the ^19^F NMR spectrum (Scheme 4B vs. 4C). Applying the same method to enantioenriched **7a** (see Table 2 for the reaction optimization) then allows for the rapid determination of the *er* as shown in Scheme 4D (the integrity of these results was confirmed by carrying out HPLC analysis of corresponding FMOC‐derivatives as well^21^).

**Table 2:** Attempted asymmetric synthesis of **5a** a

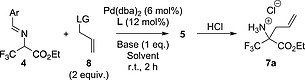							
Entry	LG	Ar	L^b^	Solv.	Base	Conv. [%]^c^	*er* (**7a**)^d^
1	OAc	Ph	**L1**	CH_2_Cl_2_	KOH (50 %)	>99	57:43
2	OAc	Ph	**L1**	toluene	KOH (50 %)	75	59:41
3	OAc	Ph	**L1**	Et_2_O	KOH (50 %)	>99	54:46
4	OAc	Ph	**L2**	toluene	KOH (50 %)	50	58:42
5	OAc	Ph	**L4**	toluene	KOH (50 %)	50	59:41
6	OAc	Ph	**L3**	toluene	KOH (50 %)	>99	65:35
7	OAc	Ph	**L3**	CH_2_Cl_2_	KOH (50 %)	80	52:48
8	OAc	Ph	**L3**	CH_3_CN	KOH (50 %)	>99	69:31
9	OAc	Ph	**L3**	CH_3_CN^e^	KOH (50 %)	>99 (78)^f^	75:25
10	OAc	Ph	**L3**	CH_3_CN^e^	K_2_CO_3_	35	70:30
11	OAc	Ph	**L3**	CH_3_CN^e^	Cs_2_CO_3_	90	70:30
12	OBoc	Ph	**L3**	CH_3_CN^e^	KOH (50 %)	>99	70:30
13	Br	Ph	**L3**	CH_3_CN^e^	KOH (50 %)	15	68:32
14	OAc	4‐NO_2_‐C_6_H_4_‐	**L3**	CH_3_CN^e^	KOH (50 %)	>99	72:28
15	OAc	4‐*t*Bu‐C_6_H_4_‐	**L3**	CH_3_CN^e^	KOH (50 %)	75	62:38
16	OAc	1‐Np	**L3**	CH_3_CN^e^	KOH (50 %)	>99	66:34

a All reactions were run at room temperature using 0.1 mmol **4** and 0.2 mmol **8** under the conditions given in the table using 1 equiv. of base in the indicated solvent (0.05 m with respect to** 4**) unless otherwise stated.

b See Scheme 3 for structures.

c Conversion based on **4** was determined by NMR analysis of crude **5**.

d Determined by ^19^F NMR analysis of **7a** with reagent **A1**.

e 0.005 m with respect to **4**.

f Isolated yield **7a**.

With a reliable and fast chiral analysis method at hand, we then screened the asymmetric Pd‐catalyzed allylation of imines **4** with allylic acceptors **8** (Table 2 gives an overview of the most significant results obtained in a very detailed screening of different conditions). First experiments between the phenyl‐based imine **4a** and allylic acetate **8a** using BINAP (**L1**) as a ligand showed that toluene may be slightly better suited than CH_2_Cl_2_ or Et_2_O with respect to asymmetric induction (entries 1–3). However, the selectivities were quite low and thus other ligands were tested next (entries 4–6). Here the DACH‐based ligand **L3** turned out to be the most selective, resulting in an *er* of 65:35 (entry 6). We then carried out a very detailed optimization with this ligand for the reaction of **4a** with **8a** (see entries 6–11 for the most interesting results). It turned out the combination of acetonitrile with aqueous KOH as a base under relatively dilute conditions gives the hydrolyzed product **7a** in high yield (78 % yield over both steps) and with a modest enantioselectivity of 75:25.

Unfortunately, this was the best result we could obtain under a variety of different conditions and also lowering the temperature or using other amounts of catalysts did not improve this outcome. Also, the analogous Me‐ester of nucleophile **4** did give more or less the same result as well. We again tested the addition of chiral PTCs but those had no positive effect either.^21^

We then changed the nature of the electrophile leaving group but neither the Boc‐protected allylic alcohol (entry 12) nor allyl bromide (entry 13) did allow for higher selectivities. Finally, the imine protecting group was varied as well (entries 14–16), but none of these groups performed better than the simple phenylimine. Accordingly, despite this reaction performs well under a variety of conditions with respect to conversion and yield, the asymmetric protocol is currently limited to an *er* of 75:25 (see entry 9 for the most selective conditions).

## Conclusions

In conclusion, we have developed a high yielding protocol for the direct allylation of easily accessible trifluoropyruvate **1**‐based imines **4** under Pd‐catalysis. This protocol gives access to a variety of different α‐allylated‐α‐CF_3_‐amino acids **5** and **7** straightforwardly and with modest enantioselectivities (up to *er* = 75:25) in the presence of chiral phosphane ligands. In addition, we also demonstrated the use of the chiral aluminum complex **A1** as an operationally simple and reliable tool to determine the enantiomeric composition of the target molecules by ^19^F NMR analysis.

## Experimental Section

General details as well as the analytical details and characterization data of all the novel compounds can be found in the online supporting information.^21^

**General racemic allylation procedure**: To a stirred solution of 3 mol‐% Pd(dba)_2_ and 3 or 6 mol‐% dppb in CH_2_Cl_2_ the corresponding acetate **8** (2 equiv.), the imine **4** (1 equiv.), and KOH (aq. 50 %, 1 equiv.) were added successively. The reaction mixture was stirred for 2 h at room temperature. After completion, the mixture was filtered through a pad of Na_2_SO_4_ and washed with Et_2_O. After evaporation of the solvent the product was purified by column chromatography with CH_2_Cl_2_ and heptanes (2:1) to yield products **5a**–**n** in the reported yields (please note that compound **5a** rapidly hydrolyses during column chromatography).

**Hydrolysis**: Compound **5a** or **5b** was dissolved in 1 mL of CH_2_Cl_2_ and 2 mL of 3 n HCl were added. The mixture was stirred at room temperature for 45 min and the layers were separated. Afterwards the aqueous layer was evaporated to dryness to get the hydrolyzed products **7a** and **7b** in quantitative yield.

Compound **5a**: Synthesized according to the general procedure on 0.1 mmol and 1 mmol scale and obtained as a yellow oil in > 95 % NMR‐yield (ISTD = Mesitylene) and with an isolated yield of 32 % after column chromatography. HRMS (ESI): *m/z* calculated for C_15_H_16_F_3_NO_2_: 300.1206 [M + H]^+^, found 300.1210. *R*_f_: 0.82 (CH_2_Cl_2_/heptane: 3:1). ^1^H NMR (300 MHz, CDCl_3_, 298 K): *δ* = 8.33 (s, 1H), 7.82–7.98 (m, 2H), 7.48–7.40 (m, 3H), 5.87–5.73 (m, 1H), 5.17–5.12 (m, 2H), 4.35–4.28 (m, 2H), 3.04–2.95 (m, 1H), 2.84–2.77 (m, 1H), 1.32 (t, *J* = 7.1 Hz, 3H); ^19^F‐NMR (282 MHz, CDCl_3_, 298 K): *δ* = –72.92 (s, 3F) ppm; ^13^C‐NMR (75 MHz, CDCl_3_, 298 K): *δ* = 166.7, 164.5, 135.5, 131.8, 130.8, 128.8, 128.7, 124.5 (q, *J* = 284.7 Hz), 120.5, 74.2 (q, *J* = 25.1 Hz), 62.3, 38.0, 14.0 ppm.

Compound **7a**: Synthesized according to the general hydrolysis procedure on 0.1 mmol scale (**5a** was directly used without purification by column chromatography). The product occurs as a white oil and in an isolated yield of 85 % (over 2 steps). HRMS (ESI): *m/z* calculated for C_8_H_13_F_3_NO_2_^+^: 212.0893 [M]^+^, found 212.0898. 1H NMR (300 MHz, MeOD, 298 K): *δ* = 5.81–5.67 (m, 1H), 5.51–5.45 (m, 2H), 4.48 (q, *J* = 7.1 Hz, 2H), 3.16–3.09 (m, 1H), 2.92–2.85 (m, 1H), 1.40 (t, *J* = 7.1 Hz, 3H); ^19^F‐NMR (282 MHz, MeOD, 298 K): *δ* = –74.91 (s, 3F) ppm; ^13^C‐NMR (75 MHz, MeOD, 298 K): *δ* = 164.5, 127.5, 125.1, 123.7 (q, *J* = 285.4 Hz), 66.1 (q, *J* = 28.9 Hz), 66.1, 36.0, 14.2 ppm.

Compound **5b**: The product was synthesized according to the general procedure on 0.1 mmol scale and 3.85 mmol scale as a white oil and with an isolated yield of 82 % (0.1 mmol scale) and 77 % (3.85 mmol scale). HRMS (ESI): *m/z* calculated for C_21_H_20_F_3_NO_2_: 376.1519 [M + H]^+^, found 376.1522. *R*_f_: 0.86 (CH_2_Cl_2_/heptane: 3:1). ^1^H NMR (300 MHz, CDCl_3_, 298 K): *δ* = 8.33 (s, 1H), 7.80–7.77 (m, 2H), 7.48–7.40 (m, 3H), 7.29–7.20 (m, 5H), 6.47 (d, *J* = 15.7 Hz, 1H), 6.20–6.10 (m, 1H), 4.36–4.28 (m, 2H), 3.17–3.11 (m, 1H), 2.97–2.89 (m, 1H),1.31 (t, *J* = 7.1 Hz, 3H); ^19^F‐NMR (282 MHz, CDCl_3_, 298 K): *δ* = –72.84 (s, 3F) ppm; ^13^C‐NMR (176 MHz, CDCl_3_, 298 K): *δ* = 166.9, 164.8, 137.1, 135.7, 135.6, 131.9, 128.9, 128.8, 128.7, 127.7, 126.4, 124.6 (q, *J* = 284.9 Hz), 122.3, 74.6 (q, *J* = 24.8 Hz), 62.5, 37.5, 14.2 ppm.

Compound **7b**: The product was synthesized according to the general hydrolysis on a 0.1 mmol scale and occurs as a white oil and with an isolated yield of 78 % (2 steps). HRMS (ESI): *m/z* calculated for C_14_H_17_F_3_NO_2_^+^: 288.1206 [M]^+^, found 288.1210. ^1^H NMR (300 MHz, CDCl_3_, 298 K): *δ* = 7.35–7.22 (m, 5H), 6.56 (d, *J* = 15.7 Hz, 1H), 6.10–6.00 (m, 1H), 4.34–4.27 (m, 2H), 3.02–2.95 (m, 1H), 2.69–2.62 (m, 1H), 1.32 (t, *J* = 7.1 Hz, 3H); ^19^F‐NMR (282 MHz, CDCl_3_, 298 K): *δ* = –74.84 (s, 3F) ppm; ^13^C‐NMR (75 MHz, CDCl_3_, 298 K): *δ* = 169.1, 136.6, 136.3, 128.8, 128.0, 126.5, 124.8 (q, *J* = 285.9 Hz), 121.2, 64.5 (q, *J* = 26.6 Hz), 62.9, 36.8, 14.2 ppm.

## Supporting information

Supporting Information
