# Supplementary material for: Pd‐Catalyzed Allylation of Imines to Access α‐CF3‐Substituted α‐Amino Acid Derivatives
Source: European J Org Chem. 2019 Nov 6;2019(42):7122–7. doi: 10.1002/ejoc.201901272 (PMC6887540; doi:10.1002/ejoc.201901272)
Supplement: Supplementary file 1 — Supporting Information [file EJOC-2019-7122-s001.pdf]

European Journal of Organic Chemistry

Supporting Information

Pd-Catalyzed Allylation of Imines to Access α-CF_3-Substituted α-Amino Acid Derivatives

Michael Winter, Hyunwoo Kim, and Mario Waser*

ejoc201901272-sup-0001-SupMat.pdf

1. General Information: 2
1.1. General Methods 2
2. Syntheses 3
2.1 Syntheses of Imines 4 3
2.2 Pd-catalyzed Allylation 4
2.2.1 Analytical Details of the Allylated Products 5a-n 5
2.3 Further transformations 11
2.3.1 Dipeptide formation 11
2.3.1 Benzoylation 12
3. Chiral Analysis 13
3.1 Copies of HPLC Chromatograms and ^{19}F-NMR spectra of 7a/10 13
3.2 Copies of HPLC Chromatograms of 5b 16
4. Asymmetric Reaction Condition Screening 18
5. Copies of NMR-Spectra of new Compounds 19

1. General Information:

1.1. General Methods

^{1}H-, ^{19}F- and ^{13}C-NMR spectra were recorded on a Bruker Avance III 300 MHz spectrometer with a broad band observe probe and a sample changer for 16 samples and on a Bruker Avance DRX 500 MHz spectrometer, which are both property to the Austro-Czech NMR-Research Center "RERI-uasb". All NMR spectra were referenced on the solvent peak. High resolution mass spectra were obtained using an Agilent 6520 Q-TOF mass spectrometer with an ESI source and an Agilent G1607A coaxial sprayer or a Thermo Fisher Scientific LTQ Orbitrap XL with an Ion Max API Source. Analyses were made in the positive ionization mode if not otherwise stated. Purine (exact mass for $[M+H]^+$ = 121.050873) and 1,2,3,4,5,6-hexakis(2,2,3,3-tetrafluoropropoxy)-1,3,5,2,4,6-triaza-triphosphinane (exact mass for $[M+H]^+$ = 922.009798) were used for internal mass calibration.

Preparative column chromatography was carried out using Davisil LC 60A 70-200 MICRON silica gel. TLC probes were detected at 254 nm or stained with an appropriate staining solution (compare section 3.1.3).

HPLC was performed using a Dionex Summit HPLC system with a Chiralcel YMC-SB (250 x 4.6 mm, 5 µm) and a Chiralpak AD-H (4.6 mm x 250 mm, 5 µm) chiral stationary phase.

All chemicals were purchased from commercial suppliers and used without further purification unless otherwise stated. All reactions were carried out under Argon.

The allyl acetate **8a** is commercially available and was purchased from Sigma Aldrich. The acetates **8b-n** were synthesized according to literature procedures.[1,2]

[1] T. Song, S. Arseniyadis, J. Cossy, *Chem.Eur.J.* **2018**, 24, 8076.
[2] J. Ruan; X. Li; O. Saidi; J. Xiao, *J. Am. Chem. Soc.* **2008**, 130, 2424.

2. Syntheses

2.1 Syntheses of Imines 4

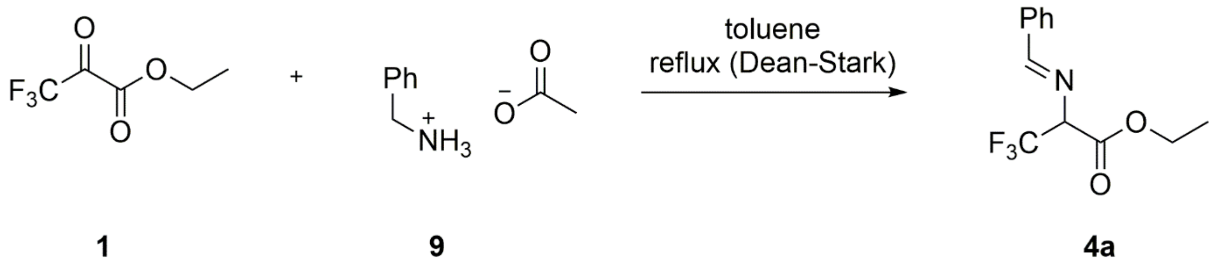

General procedure 1 (*exemplified for the synthesis of 4a*): The synthesis was performed according to literature procedure.[3] Acetic acid (0.63 mL, 11 mmol) was added to a stirred solution of benzylamine (1.2 mL, 11 mmol) in 40 mL dry toluene at room temperature and the reaction mixture turned turbid/solid (indicating the formation of the ammonium acetate **9**). Subsequently ethyl-3,3,3-trifluoropyruvate (**1**) (1.32 mL, 10 mmol) was added and the solution turned clear again. The reaction mixture was stirred at reflux under Dean-Stark conditions for 16 h. The crude product was purified by flash chromatography to yield the imine **4a** in a yield of 55% (1.42 g, 5.5 mmol).

[3] (a) H. Ohkura, D. O. Berbasov, V. A. Soloshonok, *Tetrahedron* **2003**, 59, 1647-1656. (b) M. Winter, K. Faust, M. Himmelsbach, M. Waser, *Org. Biomol. Chem.* **2019**, 17, 5731-5735.

2.2 Pd-catalyzed Allylation

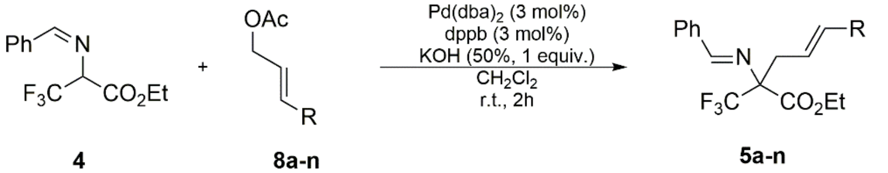

4 8a-n 5a-n

8a: R = H
8b: R = Ph
8c: R = 4-Me-C_6H_4-
8d: R = 3-Me-C_6H_4-
8e: R = 4-tBu-C_6H_4-
8f: R = 4-Ph-C_6H_4-
8g: R = 4-Br-C_6H_4-
8h: R = 4-Cl-C_6H_4-
8i: R = 4-NO_2-C_6H_4-
8j: R = 4-MeO-C_6H_4-
8k: R = 2-MeO-C_6H_4-
8l: R = 1-Naphthyl-C_6H_4-
8m: R = 2-Thienyl-C_6H_4-
8n: R = Me

General procedure 2: To a stirred solution of 3 mol% $Pd(dba)_2$ and 3 or 6 mol% dppb in CH_2Cl_2 the corresponding acetate **8** (2 equiv.), the imine **4** (1 equiv.) and KOH (aq. 50%, 1 equiv.) were added successively. The reaction mixture was stirred for 2 h at room temperature. After completion of the reaction the mixture was filtered over a pad of Na_2SO_4 and washed with Et_2O. After evaporation of the solvent the product was purified by column chromatography with CH_2Cl_2 and heptanes (2:1) to yield products **5a-n** in the reported yields (please note that compound **5a** rapidly hydrolysis during column chromatography).

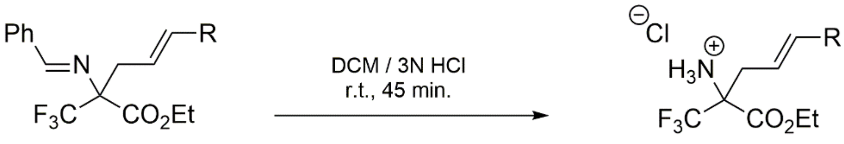

5a-b 7a-b

Hydrolysis: Compound **5a** or **5b** was dissolved in 1 mL CH_2Cl_2 and 2 mL 3N HCl were added. The mixture was stirred at room temperature for 45 min and the layers were separated. Afterwards the aqueous layer was evaporated to dryness to get the hydrolyzed products **7a** and **7b** in quantitative yield. For the chiral analysis 1 equiv. of **7** and 6 equiv. of **A1** were dissolved in 0.7 mL CD_2Cl_2 and this mixture was analyzed by ^{19}F-NMR spectroscopy.

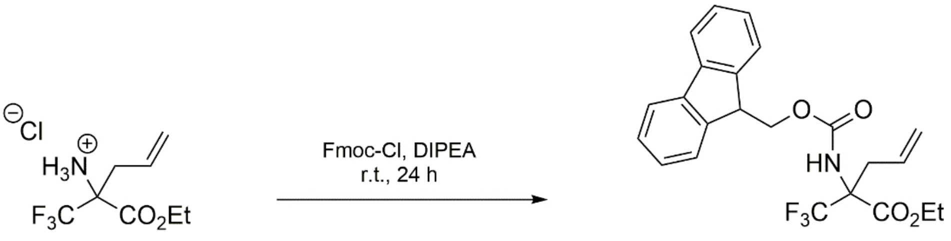

7a 10

Derivatization for HPLC analysis: The product **7a** was dissolved in 5 mL CH_2Cl_2 and 1 equiv. of Fmoc-Cl as well as 2 equiv. of DIPEA were added. The reaction mixture was stirred at room temperature for 24 h. Then the solvent was evaporated and the crude reaction mixture was purified by column chromatography with a gradient of heptanes and ethylacetate (10:1 – 5:1) to yield the Fmoc protected product **10**.

2.2.1 Analytical Details of the Allylated Products 5a-n

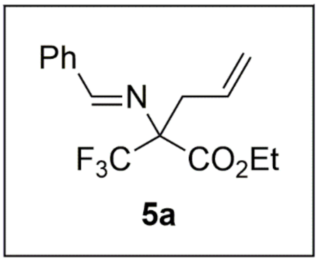

5a: The product was synthesized according to general procedure 2 on a 0.1 mmol and a 1 mmol scale and is obtained as yellow oil in a quantitative NMR-yield (ISTD=Mesitylene) and with an isolated yield of 32% after column chromatography.

HRMS (ESI): *m/z* calculated for $C_{15}H_{16}F_3NO_2$: 300.1206 $[M+H]^+$; found: 300.1210.

R_f: 0.82 (CH_2Cl_2/Heptane: 3/1).

^{1}H-NMR (300 MHz, $CDCl_3$, 298 K): δ = 8.33 (s, 1H), 7.82-7.98 (m, 2H), 7.48-7.40 (m, 3H), 5.87-5.73 (m, 1H), 5.17-5.12 (m, 2H), 4.35-4.28 (m, 2H), 3.04-2.95 (m, 1H), 2.84-2.77 (m, 1H), 1.32 (t, J = 7.1 Hz, 3H); ^{19}F-NMR (282 MHz, $CDCl_3$, 298 K): δ = -72.92 (s, 3F) ppm; ^{13}C-NMR (75 MHz, $CDCl_3$, 298 K): δ = 166.7, 164.5, 135.5, 131.8, 130.8, 128.8, 128.7, 124.5 (q, J = 284.7 Hz), 120.5, 74.2 (q, J = 25.1 Hz), 62.3, 38.0, 14.0 ppm.

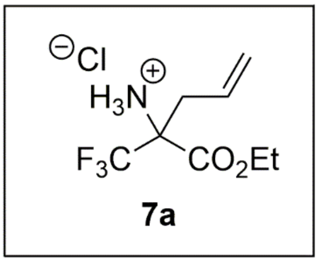

7a: The product was synthesized according to general procedure 2 on 0.1 mmol scale and instead of the column chromatography of **5a** the direct hydrolysis step was performed. The product occurs as a white oil and with an isolated yield of 85% (over 2 steps).

HRMS (ESI): *m/z* calculated for $C_8H_{13}F_3NO_2^+$: 212.0893 $[M+H]^+$; found: 212.0898.

^{1}H-NMR (300 MHz, MeOD, 298 K): δ = 5.81-5.67 (m, 1H), 5.51-5.45 (m, 2H), 4.48 (q, J = 7.1 Hz, 2H), 3.16-3.09 (m, 1H), 2.92-2.85 (m, 1H), 1.40 (t, J = 7.1 Hz, 3H); ^{19}F-NMR (282 MHz, MeOD, 298 K): δ = -74.91 (s, 3F) ppm; ^{13}C-NMR (75 MHz, MeOD, 298 K): δ = 164.5, 127.5, 125.1, 123.7 (q, J = 285.4 Hz), 66.1 (q, J = 28.9 Hz), 66.1, 36.0, 14.2 ppm.

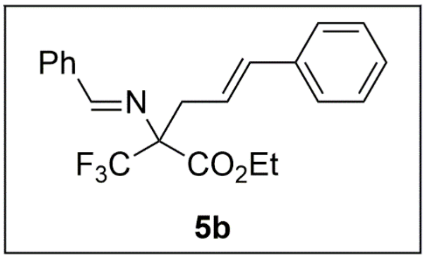

5b: The product was synthesized according to general procedure 2 on a 3.85 mmol (1g of imine starting material) scale and occurs as white oil and with an isolated yield of 77% (82% on 0.1 mmol scale).

HRMS (ESI): *m/z* calculated for $C_{21}H_{20}F_3NO_2$: 376.1519 $[M+H]^+$; found: 376.1522.

R_f: 0.86 (CH_2Cl_2/Heptane: 3/1).

^{1}H-NMR (300 MHz, $CDCl_3$, 298 K): δ = 8.33 (s, 1H), 7.80-7.77 (m, 2H), 7.48-7.40 (m, 3H), 7.29-7.20 (m, 5H), 6.47 (d, J = 15.7 Hz, 1H), 6.20-6.10 (m, 1H), 4.36-4.28 (m, 2H), 3.17-3.11 (m, 1H), 2.97-2.89 (m, 1H),1.31 (t, J = 7.1 Hz, 3H); ^{19}F-NMR (282 MHz, $CDCl_3$, 298 K): δ = -72.84 (s, 3F) ppm; ^{13}C-NMR (176 MHz, $CDCl_3$, 298 K): δ = 166.9, 164.8, 137.1, 135.7, 135.6, 131.9, 128.9, 128.8, 128.7, 127.7, 126.4, 124.6 (q, J = 284.9 Hz), 122.3, 74.6 (q, J = 24.8 Hz), 62.5, 37.5, 14.2 ppm.

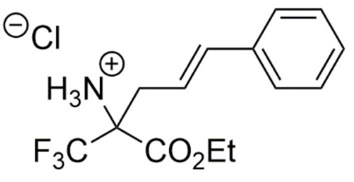

7b

<u>**7b**:</u> The product was synthesized according to general procedure 2 and the hydrolyses step successively in a 0.1 mmol scale and occurs as white oil and with an isolated yield of 78% (2 steps).

HRMS (ESI): *m/z* calculated for $C_{14}H_{17}F_3NO_2^+$: 288.1206 [M+H]$^+$; found: 288.1210.

^{1}H-NMR (300 MHz, $CDCl_3$, 298 K): δ = 7.35-7.22 (m, 5H), 6.56 (d, J = 15.7 Hz, 1H), 6.10-6.00 (m, 1H), 4.34-4.27 (m, 2H), 3.02-2.95 (m, 1H), 2.69-2.62 (m, 1H), 1.32 (t, J = 7.1 Hz, 3H); ^{19}F-NMR (282 MHz, $CDCl_3$, 298 K): δ = -74.84 (s, 3F) ppm; ^{13}C-NMR (75 MHz, $CDCl_3$, 298 K): δ = 169.1, 136.6, 136.3, 128.8, 128.0, 126.5, 124.8 (q, J = 285.9 Hz), 121.2, 64.5 (q, J = 26.6 Hz), 62.9, 36.8, 14.2 ppm.

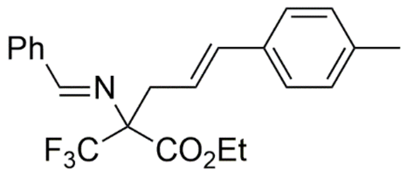

5c

<u>**5c**:</u> The product was synthesized according to general procedure 2 on a 0.1 mmol scale and occurs as white oil and with an isolated yield of 73%.

HRMS (ESI): *m/z* calculated for $C_{22}H_{22}F_3NO_2$: 390.1676 [M+H]$^+$; found: 390.1681.

R_f: 0.91 (CH_2Cl_2/Heptane: 3/1).

^{1}H-NMR (300 MHz, $CDCl_3$, 298 K): δ = 8.33 (s, 1H), 7.80-7.77 (m, 2H), 7.48-7.40 (m, 3H), 7.20-7.17 (m, 2H), 7.11-7.08 (m, 2H), 6.43 (d, J = 15.8, 1H), 6.15-6.04 (m, 1H), 4.36-4.28 (m, 2H), 3.18-3.11 (m, 1H), 2.95-2.88 (m, 1H), 2.32 (s, 3H), 1.31 (t, J = 7.1 Hz, 3H); ^{19}F-NMR (282 MHz, $CDCl_3$, 298 K): δ = -72.90 (s, 3F) ppm; ^{13}C-NMR (75 MHz, $CDCl_3$, 298 K): δ = 166.9, 164.8, 137.6, 135.7, 134.3, 131.9, 129.9, 129.4, 128.9, 128.8, 126.3, 124.5 (q, J = 283.7 Hz), 121.2, 74.7 (q, J = 24.9 Hz), 62.4, 37.4, 21.3, 14.2 ppm.

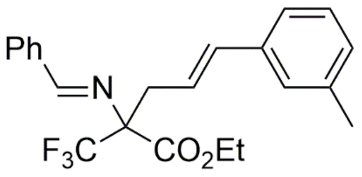

5d

<u>**5d**:</u> The product was synthesized according to general procedure 2 in a 0.1 mmol scale and occurs as white oil and with an isolated yield of 78%.

HRMS (ESI): *m/z* calculated for $C_{22}H_{22}F_3NO_2$: 390.1676 [M+H]$^+$; found: 390.1679.

R_f: 0.85 (CH_2Cl_2/Heptane: 3/1).

^{1}H-NMR (300 MHz, $CDCl_3$, 298 K): δ = 8.33 (s, 1H), 7.81-7.78 (m, 2H), 7.49-7.40 (m, 3H), 7.18-7.16 (m, 1H), 7.10-7.03 (m, 3H), 6.44 (d, J = 15.8, 1H), 6.19-6.09 (m, 1H), 4.36-4.29 (m, 2H), 3.18-3.12 (m, 1H), 2.97-2.90 (m, 1H), 2.32 (s, 3H), 1.32 (t, J = 7.1 Hz, 3H); ^{19}F-NMR (282 MHz, $CDCl_3$, 298 K): δ = -72.83 (s, 3F) ppm; ^{13}C-NMR (75 MHz, $CDCl_3$, 298 K): δ = 166.9, 164.8, 138.2, 137.0, 135.9, 131.9, 129.9, 129.1, 128.9, 128.8, 128.6, 128.5, 127.1, 124.6 (q, J = 284.8 Hz), 123.5, 122.0, 74.7 (q, J = 25.1 Hz), 62.5, 37.5, 21.5, 14.2 ppm.

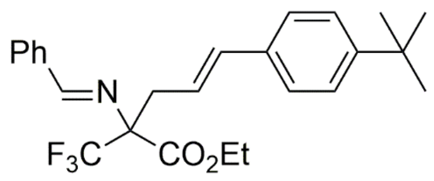

5e

<u>**5e**:</u> The product was synthesized according to general procedure 2 on a 0.1 mmol scale and occurs as a white oil and with an isolated yield of 76%.

HRMS (ESI): *m/z* calculated for $C_{25}H_{28}F_3NO_2$: 432.2145 $[M+H]^+$; found: 432.2147.

R_f: 0.84 (CH_2Cl_2/Heptane: 3/1).

^{1}H-NMR (300 MHz, $CDCl_3$, 298 K): δ = 8.34 (s, 1H), 7.81-7.78 (m, 2H), 7.49-7.40 (m, 3H), 7.33-7.31 (m, 2H), 7.25-7.22 (m, 2H), 6.46 (d, J = 16.2, 1H), 6.17-6.06 (m, 1H), 4.34-4.28 (m, 2H), 3.18-3.11 (m, 1H), 2.97-2.90 (m, 1H), 1.32-1.29 (m, 12H); ^{19}F-NMR (282 MHz, $CDCl_3$, 298 K): δ = -72.83 (s, 3F) ppm; ^{13}C-NMR (75 MHz, $CDCl_3$, 298 K): δ = 166.9, 164.7, 150.8, 135.6, 135.5, 134.3, 131.8, 129.9, 129.1, 128.9, 128.8, 126.1, 125.5, 124.6 (q, J = 285.0 Hz), 121.4, 74.7 (q, J = 25.1 Hz), 62.4, 37.5, 34.7, 31.4, 14.2 ppm.

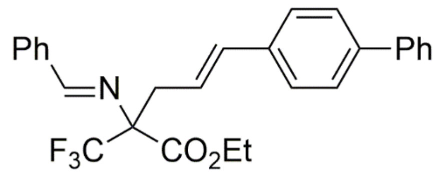

5f

<u>**5f**:</u> The product was synthesized according to general procedure 2 in a 0.1 mmol scale and occurs as white oil and with an isolated yield of 88%.

HRMS (ESI): *m/z* calculated for $C_{27}H_{24}F_3NO_2$: 452.1832 $[M+H]^+$; found: 452.1841.

R_f: 0.86 (CH_2Cl_2/Heptane: 3/1).

^{1}H-NMR (300 MHz, $CDCl_3$, 298 K): δ = 8.34 (s, 1H), 7.82-7.79 (m, 2H), 7.60-7.31 (m, 12H), 7.20-7.17 (m, 2H), 7.11-7.08 (m, 2H), 6.51 (d, J = 16.2, 1H), 6.26-6.16 (m, 1H), 4.37-4.29 (m, 2H), 3.21-3.14 (m, 1H), 3.00-2.93 (m, 1H), 1.32 (t, J = 7.1 Hz, 3H); ^{19}F-NMR (282 MHz, $CDCl_3$, 298 K): δ = -72.78 (s, 3F) ppm; ^{13}C-NMR (75 MHz, $CDCl_3$, 298 K): δ = 166.9, 164.8, 140.8, 140.5, 136.1, 135.8, 135.6, 135.3, 134.6, 132.0, 129.9, 129.1, 128.9, 128.8, 127.4, 127.1, 126.9, 126.8, 124.4 (q, J = 284.8 Hz), 122.4, 121.3, 74.7 (q, J = 25.1 Hz), 62.5, 37.6, 14.2 ppm.

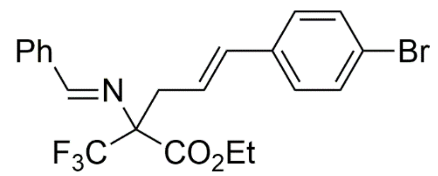

5g

<u>**5g**:</u> The product was synthesized according to general procedure 2 on a 0.1 mmol scale and occurs as a white oil and with an isolated yield of 90%.

HRMS (ESI): *m/z* calculated for $C_{21}H_{19}BrF_3NO_2$: 454.0624 $[M+H]^+$; found: 454.0631.

R_f: 0.83 (CH_2Cl_2/Heptane: 3/1).

^{1}H-NMR (300 MHz, $CDCl_3$, 298 K): δ = 8.31 (s, 1H), 7.80-7.77 (m, 2H), 7.50-7.40 (m, 3H), 7.26-7.19 (m, 4H), 6.44 (d, J = 16.0, 1H), 6.20-6.10 (m, 1H), 4.36-4.28 (m, 2H), 3.17-3.10 (m, 1H), 2.97-2.90 (m, 1H), 1.31 (t, J = 7.3 Hz, 3H); ^{19}F-NMR (282 MHz, $CDCl_3$, 298 K): δ = -72.71 (s, 3F) ppm; ^{13}C-NMR (75 MHz, $CDCl_3$, 298 K): δ = 166.8, 164.8, 135.5, 134.4, 133.4, 132.0, 128.9, 128.8, 127.5, 126.4, 124.3 (q, J = 284.6 Hz), 123.1, 74.7 (q, J = 25.2 Hz), 62.5, 37.5, 14.2 ppm.

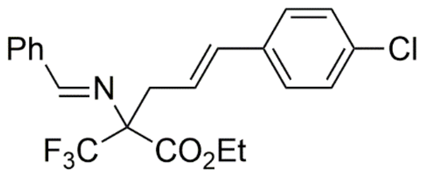

5h

5h: The product was synthesized according to general procedure 2 on a 0.1 mmol scale and occurs as a white oil and with an isolated yield of 82%.

HRMS (ESI): *m/z* calculated for $C_{21}H_{19}ClF_3NO_2$: 410.1129 $[M+H]^+$; found: 410.1136.

R_f: 0.82 (CH_2Cl_2/Heptane: 3/1).

^{1}H-NMR (300 MHz, $CDCl_3$, 298 K): δ = 8.31 (s, 1H), 7.80-7.77 (m, 2H), 7.49-7.39 (m, 5H), 7.16-7.14 (m, 2H), 6.40 (d, J = 15.8, 1H), 6.21-6.11 (m, 1H), 4.36-4.28 (m, 2H), 3.17-3.10 (m, 1H), 2.97-2.90 (m, 1H), 1.31 (t, J = 7.0 Hz, 3H); ^{19}F-NMR (282 MHz, $CDCl_3$, 298 K): δ = -72.71 (s, 3F) ppm; ^{13}C-NMR (75 MHz, $CDCl_3$, 298 K): δ = 166.8, 164.8, 136.0, 135.5, 134.9, 134.5, 132.0, 131.2, 128.9, 128.8, 127.9, 124.3 (q, J = 284.5 Hz), 123.2 74.6 (q, J = 25.1 Hz), 62.5, 37.5, 14.2 ppm.

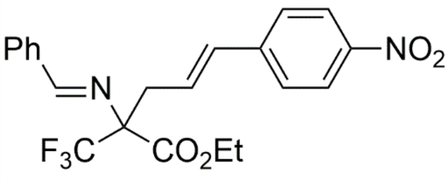

5i

5i: The product was synthesized according to general procedure 2 in a 0.1 mmol scale and occurs as white oil and with an isolated yield of 76%.

HRMS (ESI): *m/z* calculated for $C_{21}H_{19}F_3N_2O_4$: 421.1370 $[M+H]^+$; found: 421.1375.

R_f: 0.82 (CH_2Cl_2/Heptane: 3/1).

^{1}H-NMR (300 MHz, $CDCl_3$, 298 K): δ = 8.32 (s, 1H), 8.16-8.13 (m, 2H), 7.80-7.78 (m, 2H), 7.53-7.40 (m, 5H), 6.55 (d, J = 15.8, 1H), 6.44-6.34 (m, 1H), 4.38-4.30 (m, 2H), 3.20-3.13 (m, 1H), 3.05-2.98 (m, 1H), 1.31 (t, J = 7.1 Hz, 3H); ^{19}F-NMR (282 MHz, $CDCl_3$, 298 K): δ = -72.47 (s, 3F) ppm; ^{13}C-NMR (75 MHz, $CDCl_3$, 298 K): δ = 166.7, 164.8, 147.1, 143.4, 135.4, 133.4, 132.2, 128.9, 128.9, 127.8, 126.9, 124.6 (q, J = 286.0 Hz), 124.1, 74.6 (q, J = 25.4 Hz), 62.7, 37.8, 14.2 ppm.

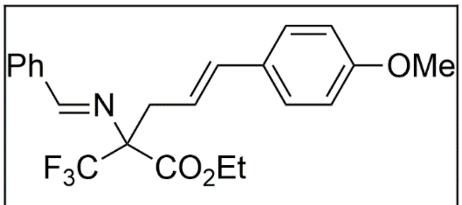

5j

5j: The product was synthesized according to general procedure 2 on 0.1 mmol scale and occurs as white oil and with an isolated yield of 91%.

HRMS (ESI): *m/z* calculated for $C_{22}H_{22}F_3NO_3$: 406.1625 $[M+H]^+$; found: 406.1628.

R_f: 0.80 (CH_2Cl_2/Heptane: 3/1).

^{1}H-NMR (300 MHz, $CDCl_3$, 298 K): δ = 8.33 (s, 1H), 7.80-7.77 (m, 2H), 7.48-7.40 (m, 3H), 7.24-7.21 (m, 2H), 6.84-6.81 (m, 2H), 6.40 (d, J = 15.3, 1H), 6.06-5.96 (m, 1H), 4.36-4.28 (m, 2H), 3.80 (s, 1H), 3.17-3.10 (m, 1H), 2.94-2.87 (m, 1H), 1.31 (t, J = 7.1 Hz, 3H); ^{19}F-NMR (282 MHz, $CDCl_3$, 298 K): δ = -72.89 (s, 3F) ppm; ^{13}C-NMR (75 MHz, $CDCl_3$, 298 K): δ = 166.9, 164.8, 159.4, 135.6, 135.2, 131.8, 129.9, 128.9, 128.9, 127.5, 124.8 (q, J = 285.6 Hz), 119.9, 114.1, 74.8 (q, J = 24.9 Hz), 62.4, 55.4, 37.4, 14.2 ppm.

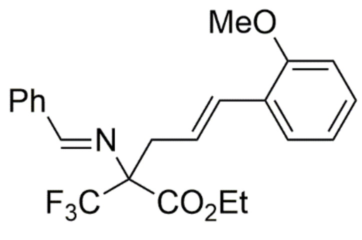

5k

5k: The product was synthesized according to general procedure 2 in a 0.1 mmol scale and occurs as white oil and with an isolated yield of 76%.

HRMS (ESI): *m/z* calculated for $C_{22}H_{22}F_3NO_3$: 406.1625 $[M+H]^+$; found: 406.1629.

R_f: 0.83 (CH_2Cl_2/Heptane: 3/1).

^{1}H-NMR (300 MHz, $CDCl_3$, 298 K): δ = 8.35 (s, 1H), 7.81-7.77 (m, 2H), 7.47-7.40 (m, 3H), 7.35-7.32 (m, 1H), 7.23-7.17 (m, 1H), 6.91-6.75 (m, 3H), 6.17-6.09 (m, 1H), 4.35-4.28 (m, 2H), 3.28 (s, 3H), 3.22-3.15 (m, 1H), 2.98-2.91 (m, 1H), 1.32 (t, J = 7.1 Hz, 3H); ^{19}F-NMR (282 MHz, $CDCl_3$, 298 K): δ = -73.07 (s, 3F) ppm; ^{13}C-NMR (75 MHz, $CDCl_3$, 298 K): δ = 166.9, 164.9, 156.7, 135.7, 131.8, 130.9, 128.9, 129.1, 128.9, 128.7, 126.9, 126.3 124.0 (q, J = 285.4 Hz), 122.7, 120.7, 110.9, 74.7 (q, J = 23.9 Hz), 62.5, 55.4, 37.6, 14.2 ppm.

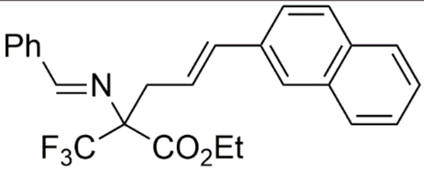

5l

5l: The product was synthesized according to the general procedure 2 on 0.1 mmol scale and occurs as a white oil and with an isolated yield of 83%.

HRMS (ESI): *m/z* calculated for $C_{25}H_{22}F_3NO_2$: 426.1676 $[M+H]^+$; found: 426.1680.

R_f: 0.80 (CH_2Cl_2/Heptane: 3/1).

^{1}H-NMR (300 MHz, $CDCl_3$, 298 K): δ = 8.38 (s, 1H), 7.91-7.75 (m, 5H), 7.51-7.33 (m, 7H), 7.21 (d, J = 15.7, 1H), 6.24-6.14 (m, 1H), 4.38-4.31 (m, 2H), 3.32-3.26 (m, 1H), 3.11-3.04 (m, 1H), 1.32 (t, J = 7.1 Hz, 3H); ^{19}F-NMR (282 MHz, $CDCl_3$, 298 K): δ = -72.89 (s, 3F) ppm; ^{13}C-NMR (75 MHz, $CDCl_3$, 298 K): δ = 166.9, 164.8, 135.6, 135.1, 134.6, 133.6, 132.0, 131.2, 129.9, 129.0, 128.8, 128.1, 126.1, 125.9, 125.7, 124.8 (q, J = 285.2 Hz), 124.2, 124.0, 74.8 (q, J = 25.1 Hz), 62.5, 37.7, 14.2 ppm.

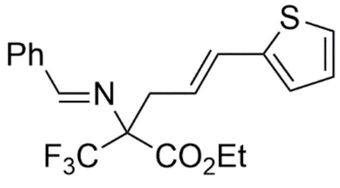

5m

5m: The product was synthesized according to general procedure 2 in a 0.1 mmol scale and occurs as white oil and with an isolated yield of 54% (this compound hydrolysis quickly during column chromatography and traces of these by products can be observed in the NMR spectra).

HRMS (ESI): *m/z* calculated for $C_{19}H_{18}F_3NO_2S$: 382.1083 $[M+H]^+$; found: 382.1095.

R_f: 0.88 (CH_2Cl_2/Heptane: 3/1).

^{1}H-NMR (300 MHz, $CDCl_3$, 298 K): δ = 8.31 (s, 1H), 7.80-7.77 (m, 2H), 7.48-7.40 (m, 3H), 7.13-7.12 (m, 1H), 6.94-6.91 (m, 1H), 6.87-6.86 (m, 1H), 6.58 (d, J = 15.8, 1H), 6.04-5.93 (m, 1H), 4.37-4.29 (m, 2H), 3.15-3.08 (m, 1H), 2.92-2.84 (m, 1H), 1.32 (t, J = 7.1 Hz, 3H); ^{19}F-NMR (282 MHz, $CDCl_3$, 298 K): δ = -72.98 (s, 3F) ppm; ^{13}C-NMR (75 MHz, $CDCl_3$, 298 K): δ = 166.8,

164.8, 142.0, 135.6, 131.9, 128.9, 128.8, 127.5, 125.6, 124.6 (q, J = 283.0 Hz), 124.3, 121.9, 74.6 (q, J = 25.1 Hz), 62.5, 37.2, 14.2 ppm.

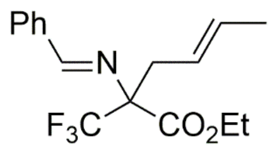

5n

5n: The product was synthesized according to general procedure 2 in a 0.1 mmol scale and occurs as white oil and with an isolated yield of 28%.

HRMS (ESI): *m/z* calculated for $C_{16}H_{18}F_3NO_2$: 314.1368 $[M+H]^+$; found: 314.1371.

R_f: 0.76 (CH_2Cl_2/Heptane: 3/1).

Product peaks from the ^{1}H and ^{19}F NMR of the isolated but not pure product (this compound tends to decompose relatively quickly): ^{1}H-NMR (300 MHz, $CDCl_3$, 298 K): δ = 8.29 (s, 1H), 7.81-7.78 (m, 2H), 7.47-7.40 (m, 3H), 5.53-5.36 (m, 2H), 4.34-4.27 (m, 2H), 2.97-2.90 (m, 1H), 2.73-2.66 (m, 1H), 1.64 (d, J = 6.1 Hz, 3H), 1.31 (t, J = 7.1 Hz, 3H); ^{19}F-NMR (282 MHz, $CDCl_3$, 298 K): δ = -73.01 (s, 3F) ppm.

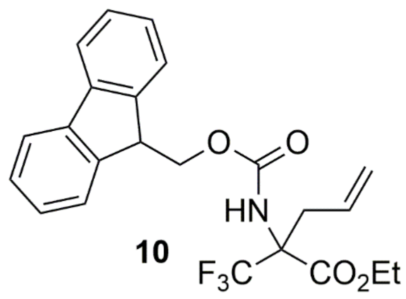

10

10: The product was synthesized according to the above described preparation for HPLC analysis on a 0.05 mmol scale and occurs as clear liquid with an isolated yield of 18% after column chromatography.

MS (ESI): *m/z* calculated for $C_{23}H_{22}F_3NO_4$: 434.15 $[M+H]^+$; found: 434.16.

R_f: 0.69 (Heptanes/Ethylacetate: 1/1).

^{1}H-NMR (300 MHz, $CDCl_3$, 298 K): δ = 7.78-7.76 (m, 2H), 7.60-7.57 (m, 2H), 7.43-7.39 (m, 2H), 7.35-7.29 (m, 2H), 5.86 (s, 1H), 5.56-5.52 (m, 1H), 5.24-5.15 (m, 2H), 4.48-4.31 (m, 4H), 4.26-4.21 (m, 1H), 3.63-3.55 (m, 1H), 2.86-2.79 (m, 1H), 1.33 (t, J = 7.1 Hz, 3H); ^{19}F-NMR (282 MHz, $CDCl_3$, 298 K): δ = -74.06 (s, 3F) ppm;

2.3 Further transformations

2.3.1 Dipeptide formation

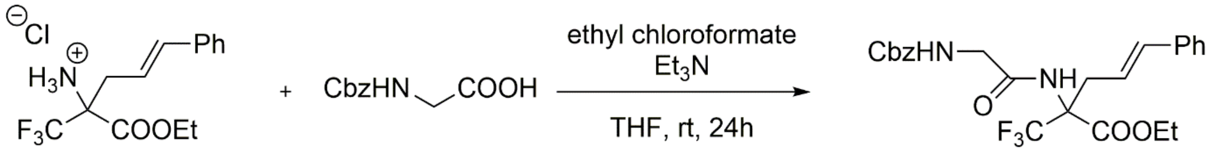

7b **11** **12**

The dipeptide **12** was synthesized according to a modified literature procedure[4]. Compound **7b** (0.1 mmol) and 1 equiv. of the Cbz-protected glycine **11** were dissolved in 1 mL THF, then 2 equiv. of Et_3N and 1.2 equiv. of ethyl chloroformate were added. The mixture was stirred at room temperature for 24 h. Afterwards the reaction was partitioned between water and dichloromethane. The organic layer was washed with $NaHCO_3$, water and brine, and dried with Na_2SO_4. After evaporation of the solvent, the dipeptide **12** was purified by column chromatography with DCM/heptanes (2/1) and an isolated yield of 72 %.

HRMS (ESI): *m/z* calculated for $C_{24}H_{25}F_3N_2O_5$: 479.1788 $[M+H]^+$; found: 479.1787.

R_f: 0.33 (DCM/heptanes: 2:1)

^{1}H-NMR (300 MHz, $CDCl_3$, 298 K): δ = 7.33-7.20 (m, 10H), 6.90 (s, 1H), 6.51 (d, J = 15.3 Hz, 1H), 5.97-5.89 (m, 1H), 5.30 (s, 1H), 5.07 (s, 1H), 4.35 (q, J = 7.3 Hz, 2H), 3.91 (s, 1H), 3.89 (s, 1H), 3.76-3.69 (m, 1H), 3.04-2.96 (m, 1H), 1.33 (t, J = 7.2 Hz, 3H); ^{19}F-NMR (282 MHz, $CDCl_3$, 298 K): δ = -73.12 (s, 3F) ppm; ^{13}C-NMR (125 MHz, $CDCl_3$, 298 K): δ = 176.1, 168.4, 166.4, 136.6, 136.1, 128.7, 128.4, 128.3, 128.0, 126.6, 123.9 (q, J = 286 Hz), 120.7, 67.5, 66.0 (q, J = 30.7), 63.8, 45.4, 32.9, 14.1 ppm.

[4] P. Singh, S. Kaur, J. Kaur, G. Singh, R. Bhatti, *J. Med. Chem.* **2016**, 59, 8, 3920-3934.

2.3.1 Benzoylation

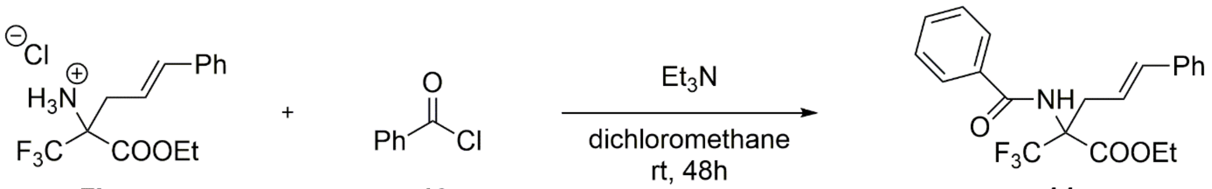

7b **13** **14**

For the benzoylation, compound **7b** (0.1 mmol) was dissolved in 1 mL dichloromethane, then 2 equiv. of Et_3N and 2 equiv. of benzoylchloride were added. The mixture was stirred at room temperature for 48 h. Afterwards the reaction was quenched with water and the phases were separated. The organic layer was dried with Na_2SO_4 and after evaporation of the solvent the benzoylated amino ester was purified by column chromatography with DCM/heptanes (2/1) and an isolated yield of 45 %.

HRMS (ESI): *m/z* calculated for $C_{21}H_{20}F_3NO_3$: 392.1474 $[M+H]^+$; found: 392.1471.

R_f: 0.46 (DCM/heptanes: 2:1)

^{1}H-NMR (300 MHz, $CDCl_3$, 298 K): δ = 7.80-7.77 (m, 2H), 7.56-7.51 (m, 1H), 7.47-7.42 (m, 2H), 7.28-7.20 (m, 5H), 7.04 (s, 1H), 6.59 (d, J = 16.1 Hz, 1H), 6.05-5.95 (m, 1H), 4.40 (q, J = 7.1 Hz, 2H), 3.97-3.90 (m, 1H), 3.16-3.08 (m, 1H), 1.37 (t, J = 7.1 Hz, 3H); ^{19}F-NMR (282 MHz, $CDCl_3$, 298 K): δ = -72.95 (s, 3F) ppm.

3. Chiral Analysis

3.1 Copies of HPLC Chromatograms and ^{19}F-NMR spectra of 7a/10

HPLC conditions:

Column: Chiralpak AD-H (4.6 mm x 250 mm, 5 μm) chiral stationary phase

Flow: 0.5 mL/min

Temperature: 10 °C

Hexane/IPA: 95/5

HPLC of the racemic sample of **10**:

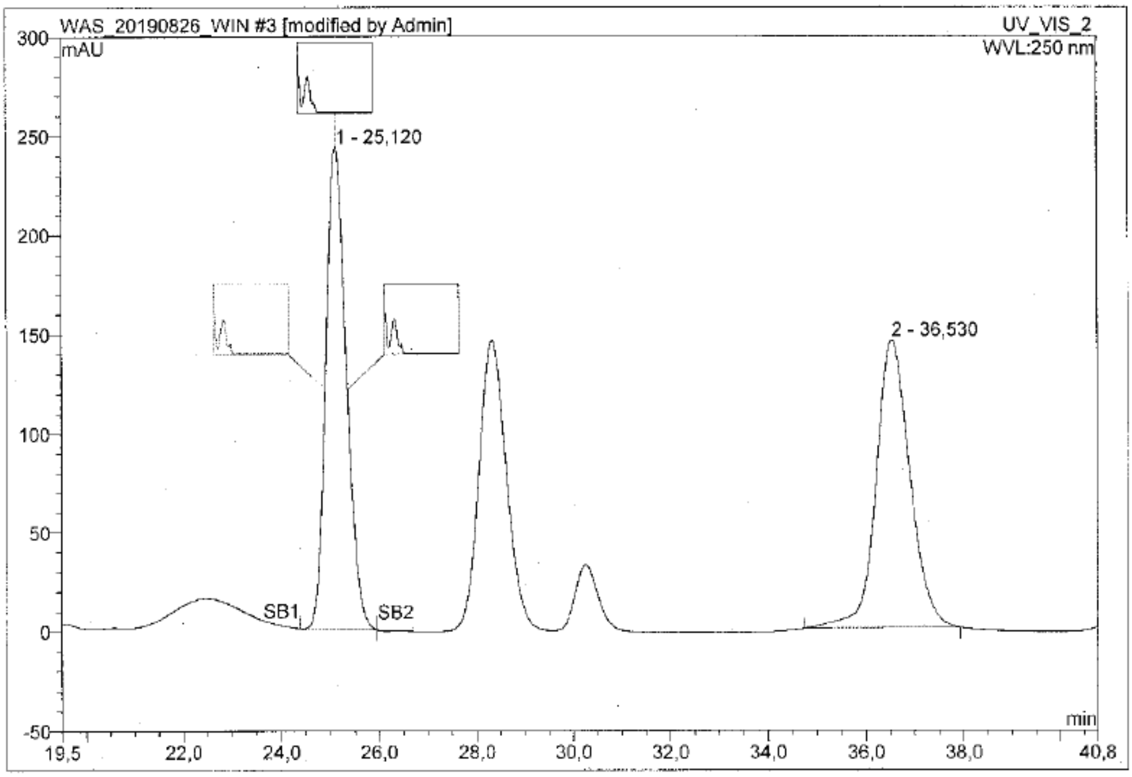

No.	Ret.Time min	Peak Name	Height mAU	Area mAU*min	Rel.Area %	Amount	Type
1	25,12	n.a.	243,515	118,007	50,15	n.a.	MB*
2	36,53	n.a.	145,519	117,303	49,85	n.a.	BMB*
Total:			389,034	235,310	100,00	0,000	

^{19}F-NMR of the racemic sample of **7a**:

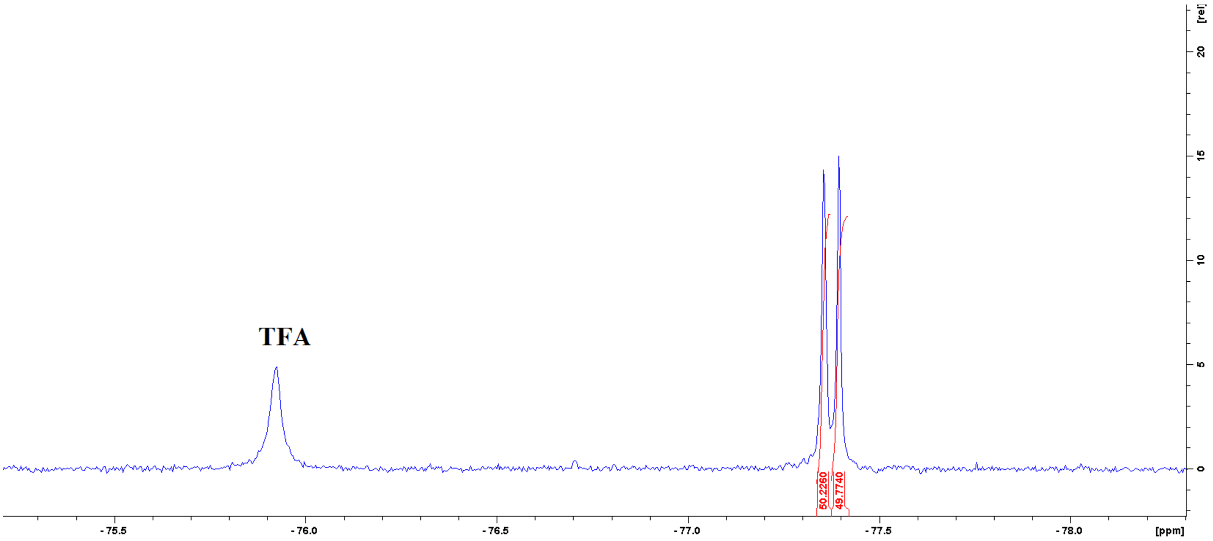

HPLC of an enantiomeric enriched sample of **10**:

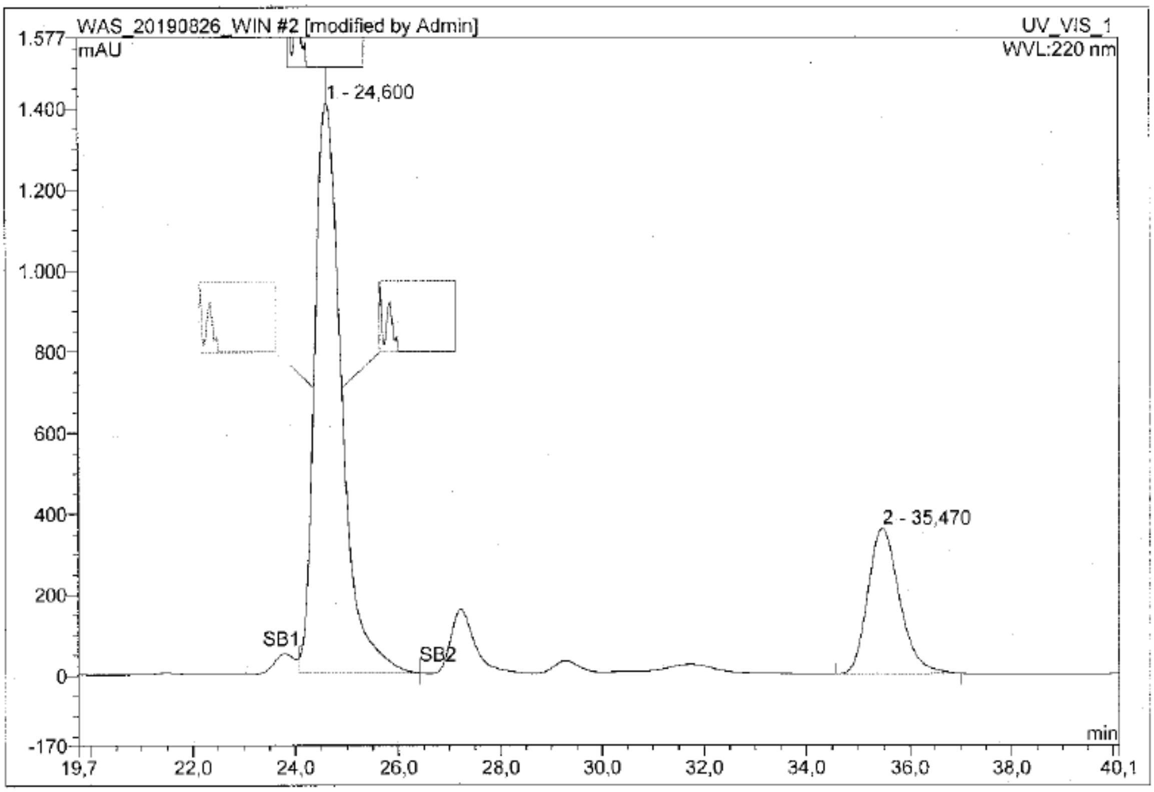

No.	Ret.Time min	Peak Name	Height mAU	Area mAU*min	Rel.Area %	Amount	Type
1	24,60	n.a.	1407,922	851,607	76,82	n.a.	MB*
2	35,47	n.a.	360,876	256,924	23,18	n.a.	BMB*
Total:			1768,798	1108,531	100,00	0,000	

^{19}F-NMR of an enantiomeric enriched sample of **7a**:

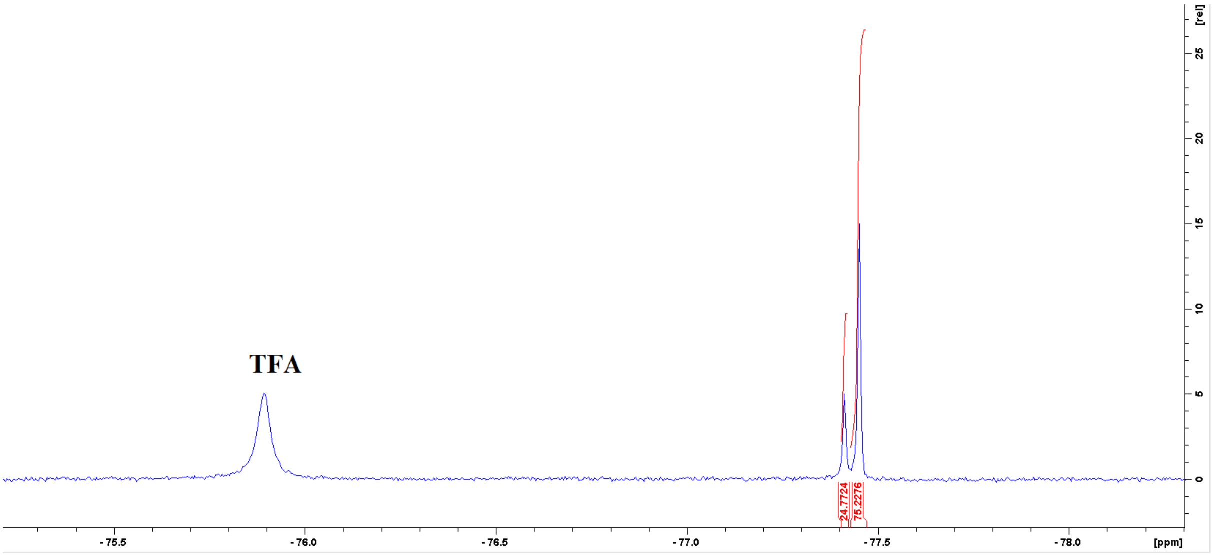

3.2 Copies of HPLC Chromatograms of 5b

HPLC Conditions:

Column: Chiralcel YMC-SB (250 x 4.6 mm, 5 μm) chiral stationary phase

Flow: 0.5 mL/min

Temperature: 10 °C

Hexane/IPA: 95/5

Racemic sample of **5b**:

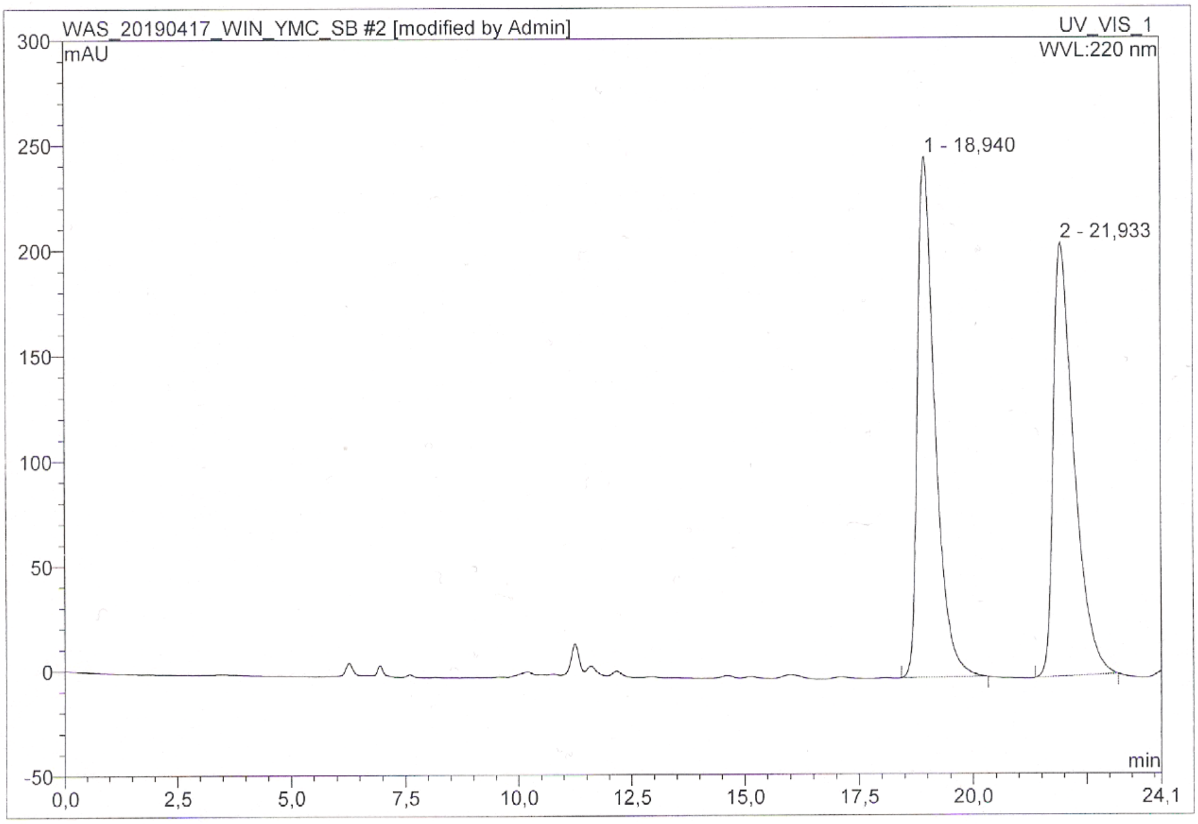

No.	Ret.Time min	Peak Name	Height mAU	Area mAU*min	Rel.Area %	Amount	Type
1	18,94	n.a.	246,994	109,902	49,81	n.a.	BMB*
2	21,93	n.a.	205,854	110,741	50,19	n.a.	BMB*
Total:			452,847	220,643	100,00	0,000	

Enantiomeric enriched sample of **5b**:

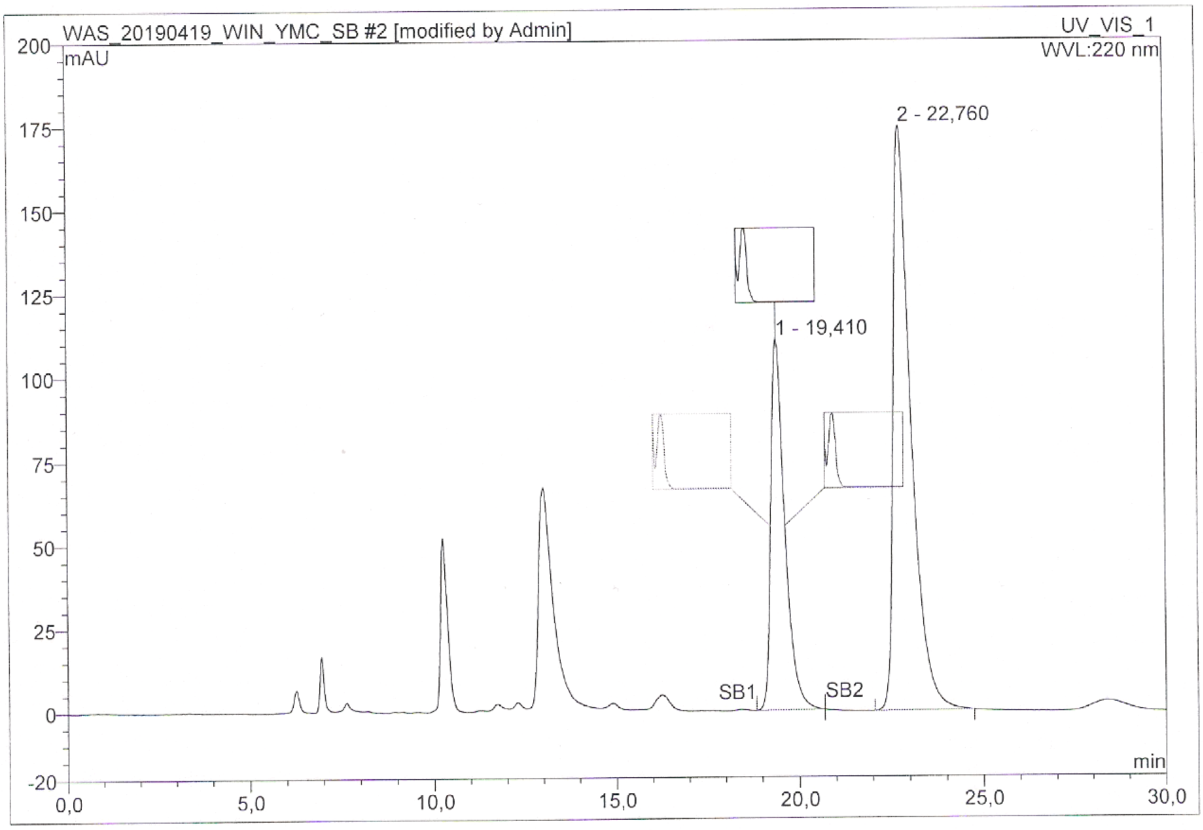

No.	Ret.Time min	Peak Name	Height mAU	Area mAU*min	Rel.Area %	Amount	Type
1	19,41	n.a.	110,504	48,692	33,43	n.a.	BMB*
2	22,76	n.a.	174,500	96,950	66,57	n.a.	BMB*
Total:			285,004	145,642	100,00	0,000	

4. Asymmetric Reaction Condition Screening

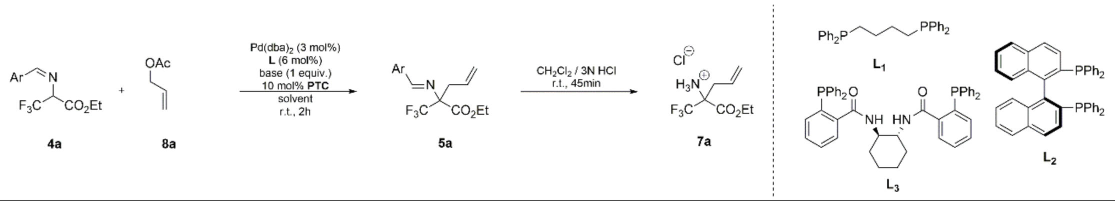

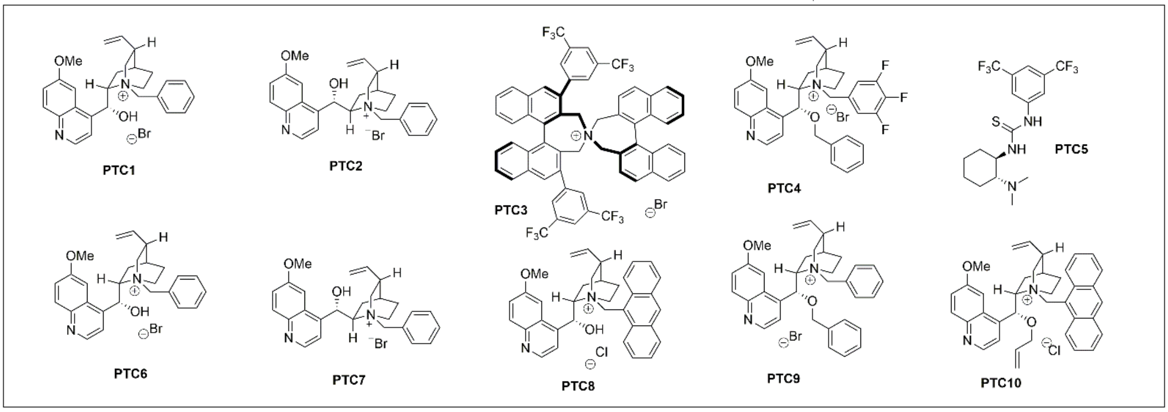

entry	Ar	solvent	conc. [M]	base	ligand	PTC	conv. [%]	ee [%]
**1**	Ph	toluene	0.05	aq. KOH (50%)	L1	PTC1	>99	0
**2**	Ph	toluene	0.05	aq. KOH (50%)	L1	PTC2	>99	0
**3**	Ph	toluene	0.05	K_2CO_3	L2	PTC1	89	26
**4**	Ph	toluene	0.05	aq. KOH (50%)	L2	PTC1	>99	24
**5**	Ph	toluene	0.05	$CsOH.H_2O$	L2	PTC1	79	22
**6**	Ph	toluene	0.05	Cs_2CO_3	L2	PTC1	59	18
**7**	Ph	toluene	0.05	aq. KOH (50%)	L2	PTC2	>99	20
**8**	Ph	CH_2Cl_2	0.05	aq. KOH (50%)	L2	PTC1	>99	12
**9**	Ph	CH_2Cl_2	0.05	K_2CO_3	L2	PTC1	23	22
**10**	Ph	CH_2Cl_2	0.05	K3PO4	L2	PTC1	79	4
**11**	Ph	CH_2Cl_2	0.05	NaHCO3	L2	PTC1	18	12
**12**	Ph	toluene	0.05	aq. KOH (50%)	L2	PTC3	>99	18
**13**	4-NO_2-Ph	toluene	0.005	aq. KOH (50%)	L3	PTC1	73	-46
**14**	4-NO_2-Ph	toluene	0.005	aq. KOH (50%)	L3	PTC4	98	-38
**15**	4-NO_2-Ph	toluene	0.005	aq. KOH (50%)	L3	PTC3	22	-48
**16**	4-NO_2-Ph	toluene	0.005	aq. KOH (50%)	L3	PTC5	17	-40
**17**	4-NO_2-Ph	toluene	0.005	aq. KOH (50%)	L3	PTC2	53	-28
**18**	4-NO_2-Ph	toluene	0.005	aq. KOH (50%)	L3	PTC6	65	-40
**19**	4-NO_2-Ph	toluene	0.005	aq. KOH (50%)	L3	PTC7	81	-26
**20**	4-NO_2-Ph	toluene	0.005	aq. KOH (50%)	L3	PTC8	33	-36
**21**	4-NO_2-Ph	toluene	0.005	aq. KOH (50%)	L3	PTC9	>99	-40
**22**	4-NO_2-Ph	toluene	0.005	aq. KOH (50%)	L3	PTC10	81	-46
**23**	4-NO_2-Ph	AcN	0.005	aq. KOH (50%)	L3	PTC1	>99	-48
**24**	4-NO_2-Ph	AcN	0.005	aq. KOH (50%)	L3	PTC4	>99	-46
**25**	4-NO_2-Ph	AcN	0.005	aq. KOH (50%)	L3	PTC3	>99	-46
**26**	4-NO_2-Ph	AcN	0.005	aq. KOH (50%)	L3	PTC5	>99	-42

5. Copies of NMR-Spectra of new Compounds

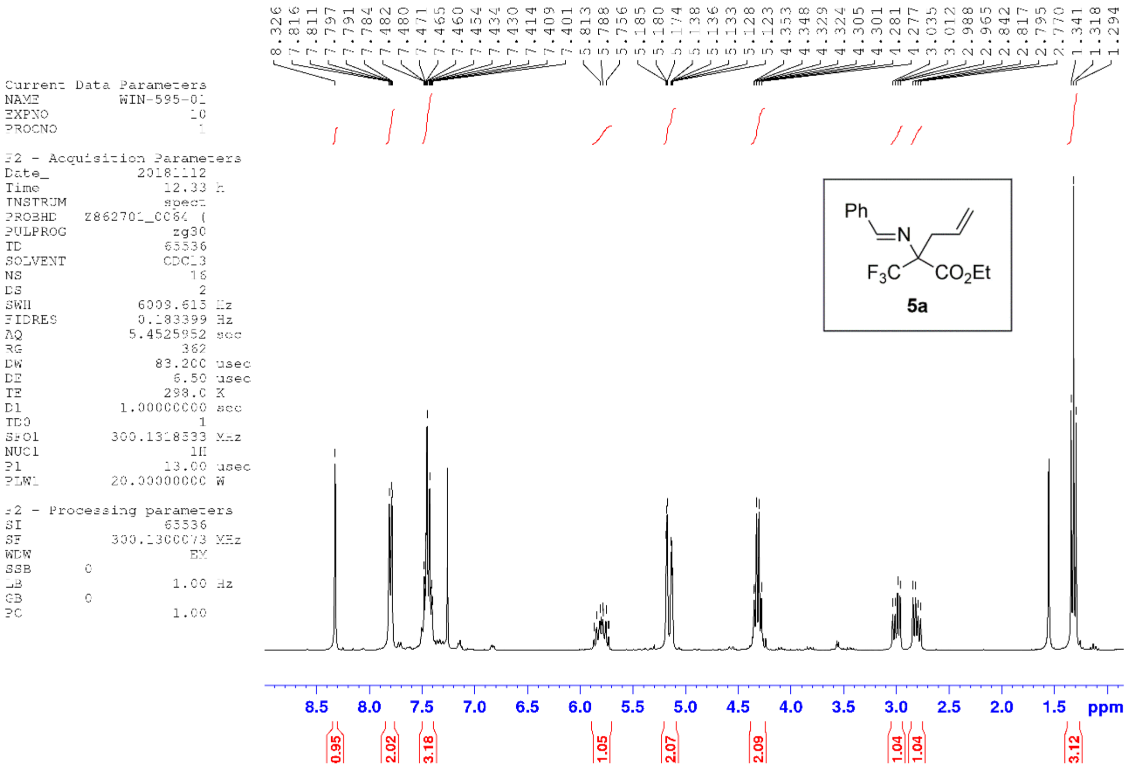

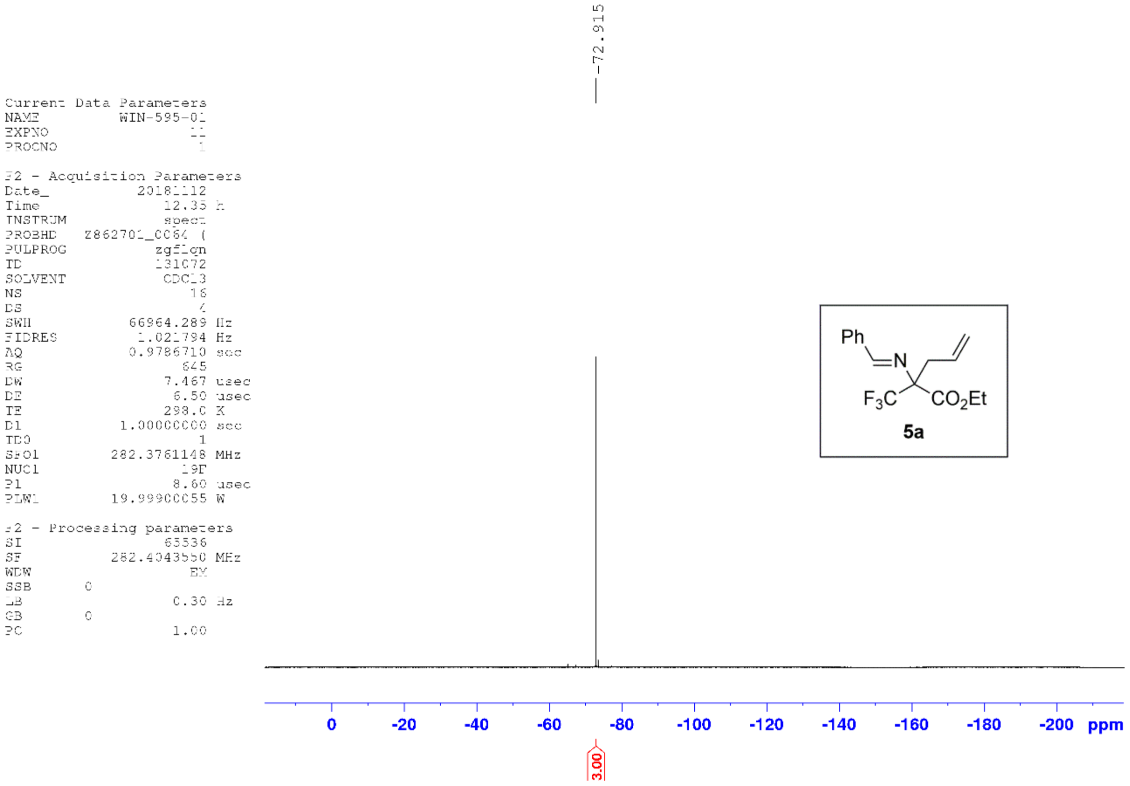

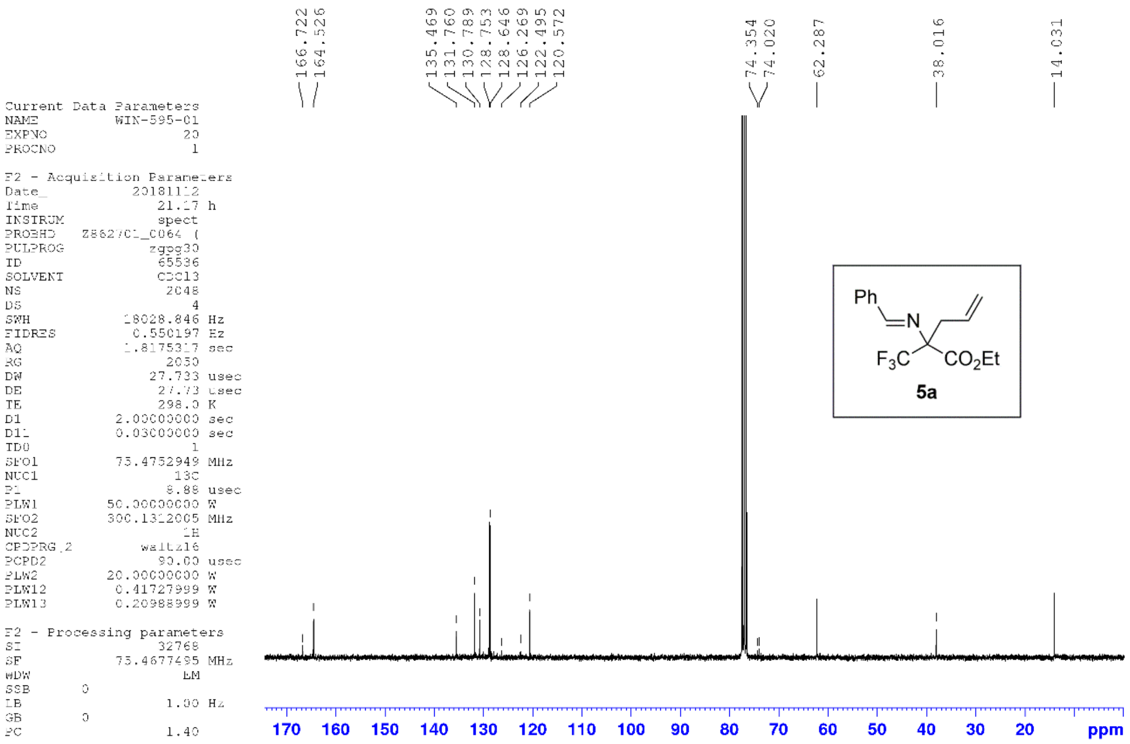

Current Data Parameters
NAME WIN-595-01
EXPNO 20
PROCNO 1
F2 - Acquisition Parameters
Date_ 20181112
Time 21.17 h
INSTRUM spect
PROBHD Z862701_0064 (
PULPROG zgpg30
TD 65536
SOLVENT CDCl3
NS 2048
DS 4
SWH 18028.846 Hz
FIDRES 0.550197 Hz
AQ 1.8175317 sec
RG 2050
DW 27.733 usec
DE 27.73 usec
TE 298.0 K
D1 2.00000000 sec
D11 0.03000000 sec
TD0 1
SFO1 75.4752949 MHz
NUC1 13C
P1 8.88 usec
PLW1 50.00000000 W
SFO2 300.1312005 MHz
NUC2 1H
CPDPRG_2 waltz16
PCPD2 90.00 usec
PLW2 20.00000000 W
PLW12 0.41727999 W
PLW13 0.20988999 W
F2 - Processing parameters
SI 32768
SF 75.4677495 MHz
WDW EM
SSB 0
LB 1.00 Hz
GB 0
PC 1.40
166.722
164.526
135.469
131.760
130.789
128.753
128.646
126.269
122.495
120.572
74.354
74.020
62.287
38.016
14.031
Ph
N
F3C
CO2Et
5a
170 160 150 140 130 120 110 100 90 80 70 60 50 40 30 20 ppm

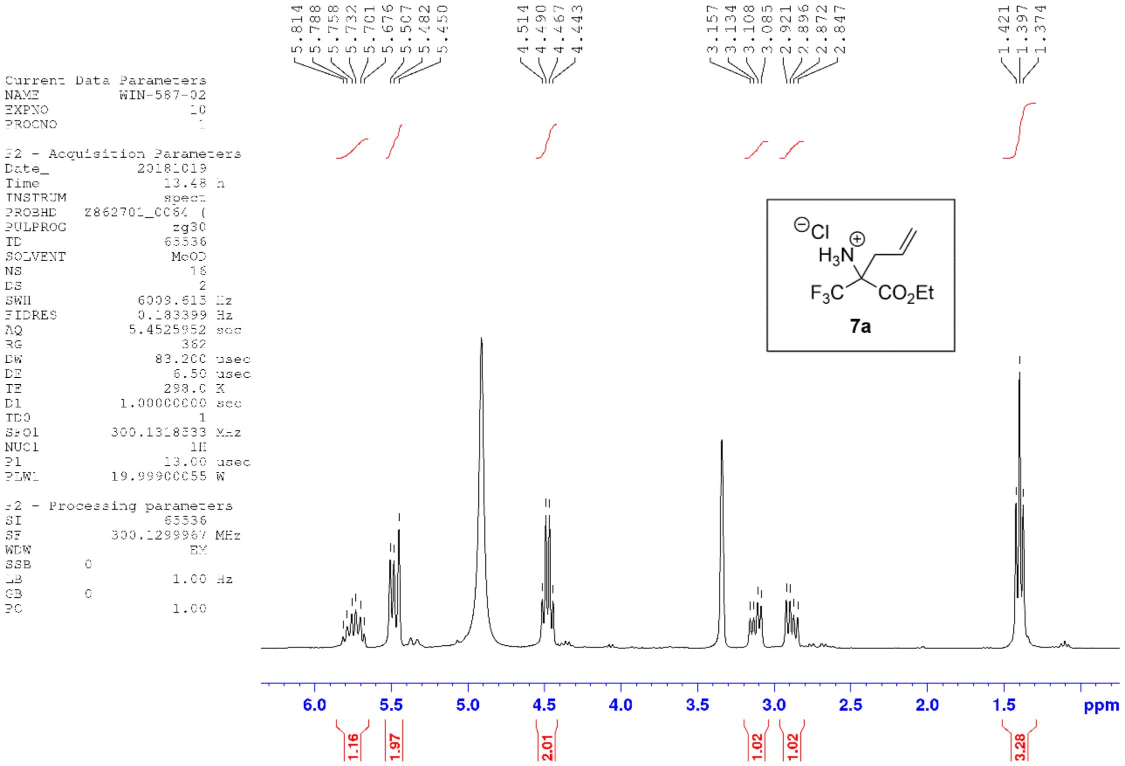

Current Data Parameters
NAME WIN-587-02
EXPNO 10
PROCNO 1
F2 - Acquisition Parameters
Date_ 20181019
Time 13.48 h
INSTRUM spect
PROBHD Z862701_0064 (
PULPROG zg30
TD 65536
SOLVENT MeOD
NS 16
DS 2
SWH 6009.615 Hz
FIDRES 0.183399 Hz
AQ 5.4525952 sec
RG 362
DW 83.200 usec
DE 6.50 usec
TE 298.0 K
D1 1.00000000 sec
TD0 1
SFO1 300.1318533 MHz
NUC1 1H
P1 13.00 usec
PLW1 19.99900055 W
F2 - Processing parameters
SI 65536
SF 300.1299967 MHz
WDW EM
SSB 0
LB 1.00 Hz
GB 0
PC 1.00
5.814
5.788
5.758
5.732
5.701
5.676
5.507
5.482
5.450
4.514
4.490
4.467
4.443
3.157
3.134
3.108
3.085
2.921
2.896
2.872
2.847
1.421
1.397
1.374
Cl
H3N
F3C
CO2Et
7a
6.0 5.5 5.0 4.5 4.0 3.5 3.0 2.5 2.0 1.5 ppm
1.16
1.97
2.01
1.02
1.02
3.28

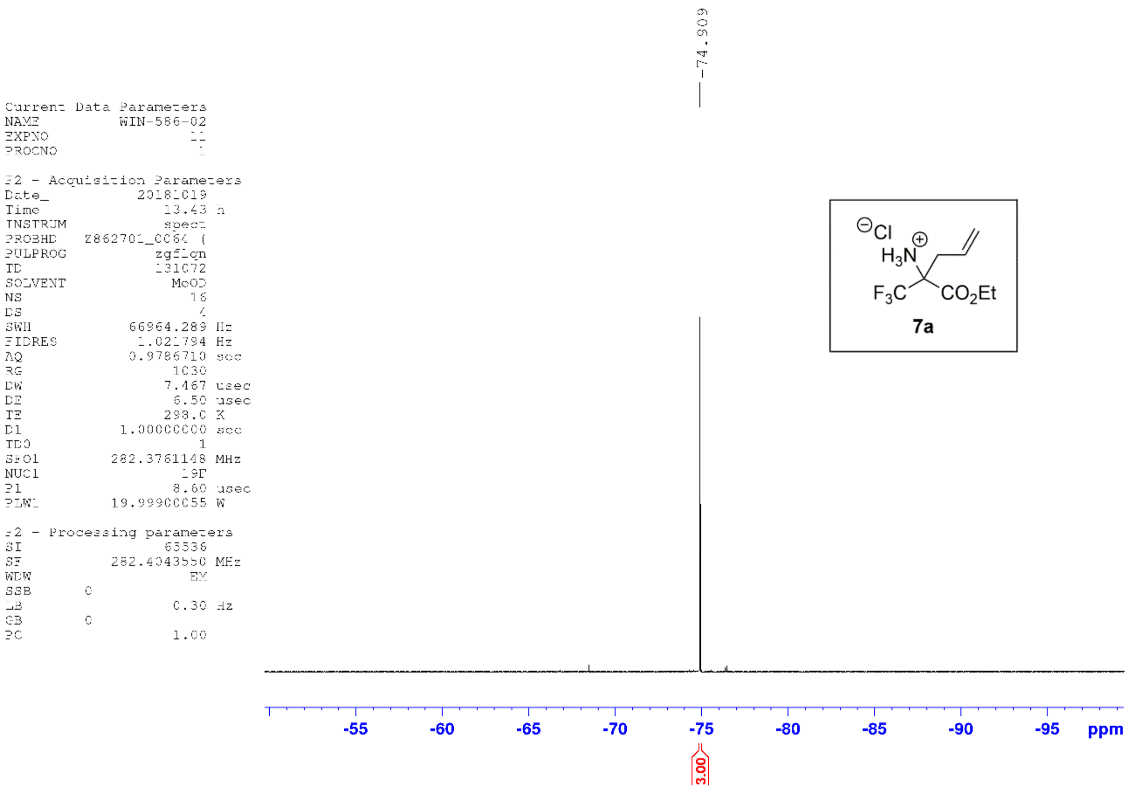

-74.909
Current Data Parameters
NAME WIN-586-02
EXPNO 11
PROCNO 1
F2 - Acquisition Parameters
Date_ 20181019
Time 13.43 h
INSTRUM spect
PROBHD Z862701_0064 (
PULPROG zgflqn
TD 131072
SOLVENT MeOD
NS 16
DS 4
SWH 66964.289 Hz
FIDRES 1.021794 Hz
AQ 0.9786710 sec
RG 1030
DW 7.467 usec
DE 6.50 usec
TE 298.0 K
D1 1.00000000 sec
TD0 1
SFO1 282.3781148 MHz
NUC1 19F
P1 8.60 usec
PLW1 19.99900055 W
F2 - Processing parameters
SI 65536
SF 282.4043550 MHz
WDW EM
SSB 0
LB 0.30 Hz
GB 0
PC 1.00
Cl
H3N
F3C
CO2Et
7a
-55
-60
-65
-70
-75
-80
-85
-90
-95
ppm
3.00

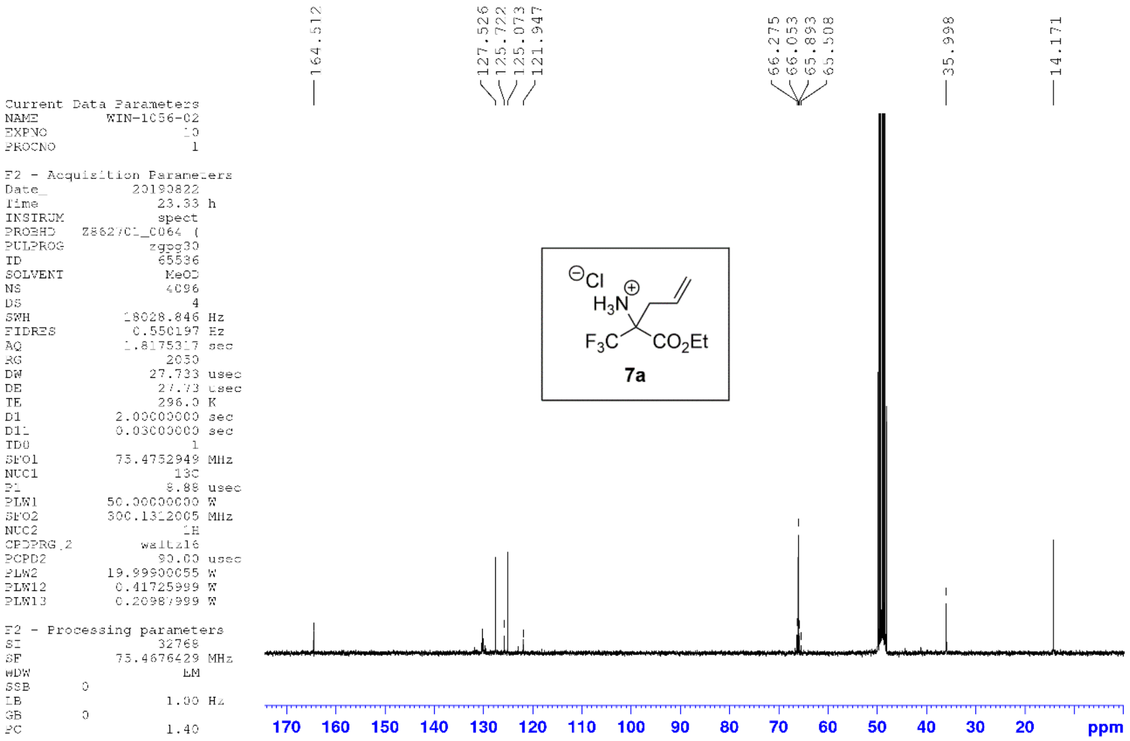

164.512
127.526
125.722
125.073
121.947
66.275
66.053
65.893
65.508
35.998
14.171
Current Data Parameters
NAME WIN-1056-02
EXPNO 10
PROCNO 1
F2 - Acquisition Parameters
Date_ 20190822
Time 23.33 h
INSTRUM spect
PROBHD Z862701_0064 (
PULPROG zgpg30
TD 65536
SOLVENT MeOD
NS 4096
DS 4
SWH 18028.846 Hz
FIDRES 0.550197 Hz
AQ 1.8175317 sec
RG 2050
DW 27.733 usec
DE 27.73 usec
TE 296.3 K
D1 2.00000000 sec
D11 0.03000000 sec
TD0 1
SFO1 75.4752949 MHz
NUC1 13C
P1 8.88 usec
PLW1 50.00000000 W
SFO2 300.1312005 MHz
NUC2 1H
CPDPRG[2] waltz16
PCPD2 90.00 usec
PLW2 19.99900055 W
PLW12 0.41725999 W
PLW13 0.20987999 W
F2 - Processing parameters
SI 32768
SF 75.4676429 MHz
WDW EM
SSB 0
LB 1.00 Hz
GB 0
PC 1.40
Cl
H3N
F3C
CO2Et
7a
170
160
150
140
130
120
110
100
90
80
70
60
50
40
30
20
ppm

Current Data Parameters
NAME WIN-603-02
EXPNO 10
PROCNO 1

F2 - Acquisition Parameters
Date_ 20180409
Time 14.13 h
INSTRUM spect
PROBHD Z862701_0064 (
PULPROG zg30
TD 65536
SOLVENT CDCl3
NS 16
DS 2
SWH 6009.615 Hz
FIDRES 0.183399 Hz
AQ 5.4525952 sec
RG 512
DW 83.200 usec
DE 6.50 usec
TE 298.0 K
D1 1.00000000 sec
TD0 1
SFO1 300.1318533 MHz
NUC1 1H
P1 13.00 usec
PLW1 20.00000000 W

F2 - Processing parameters
SI 65536
SF 300.1300073 MHz
WDW EM
SSB 0
LB 0.30 Hz
GB 0
PC 1.00

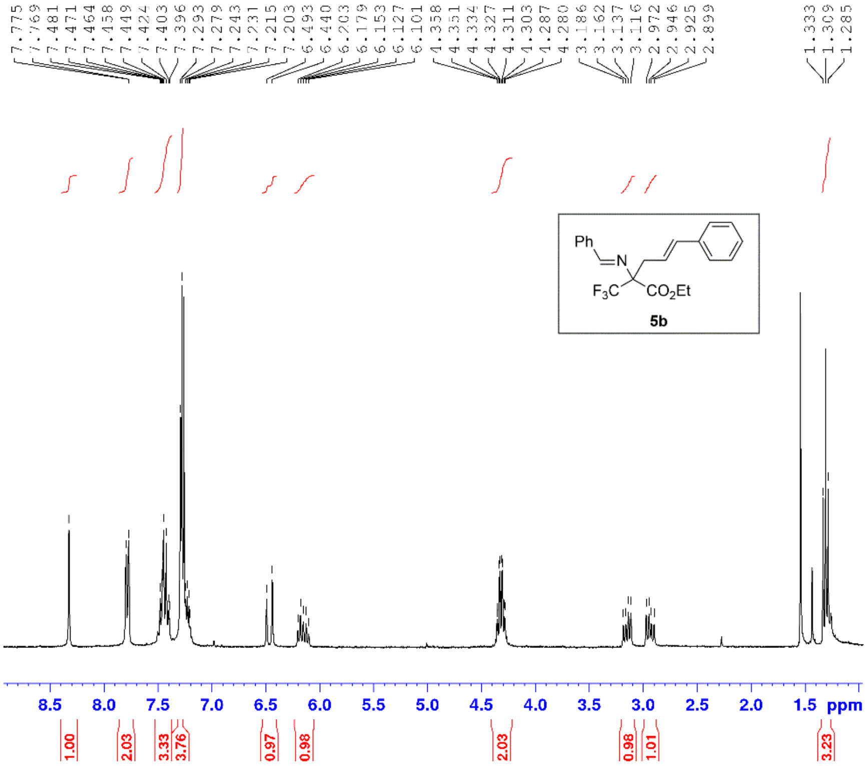

Current Data Parameters
NAME WIN-603-02
EXPNO 11
PROCNO 1

F2 - Acquisition Parameters
Date_ 20180409
Time 14.15 h
INSTRUM spect
PROBHD Z862701_0064 (
PULPROG zgflqn
TD 131072
SOLVENT CDCl3
NS 16
DS 4
SWH 66964.289 Hz
FIDRES 1.021794 Hz
AQ 0.9786710 sec
RG 645
DW 7.467 usec
DE 6.50 usec
TE 298.0 K
D1 1.00000000 sec
TD0 1
SFO1 282.3761148 MHz
NUC1 19F
P1 8.60 usec
PLW1 19.99900055 W

F2 - Processing parameters
SI 65536
SF 282.4043550 MHz
WDW EM
SSB 0
LB 0.30 Hz
GB 0
PC 1.00

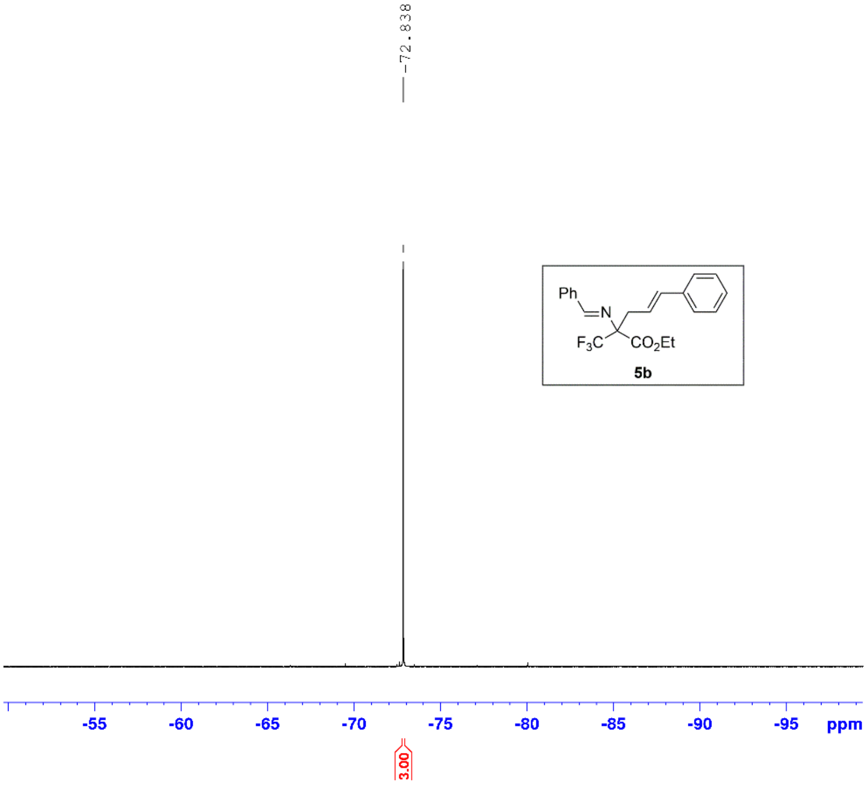

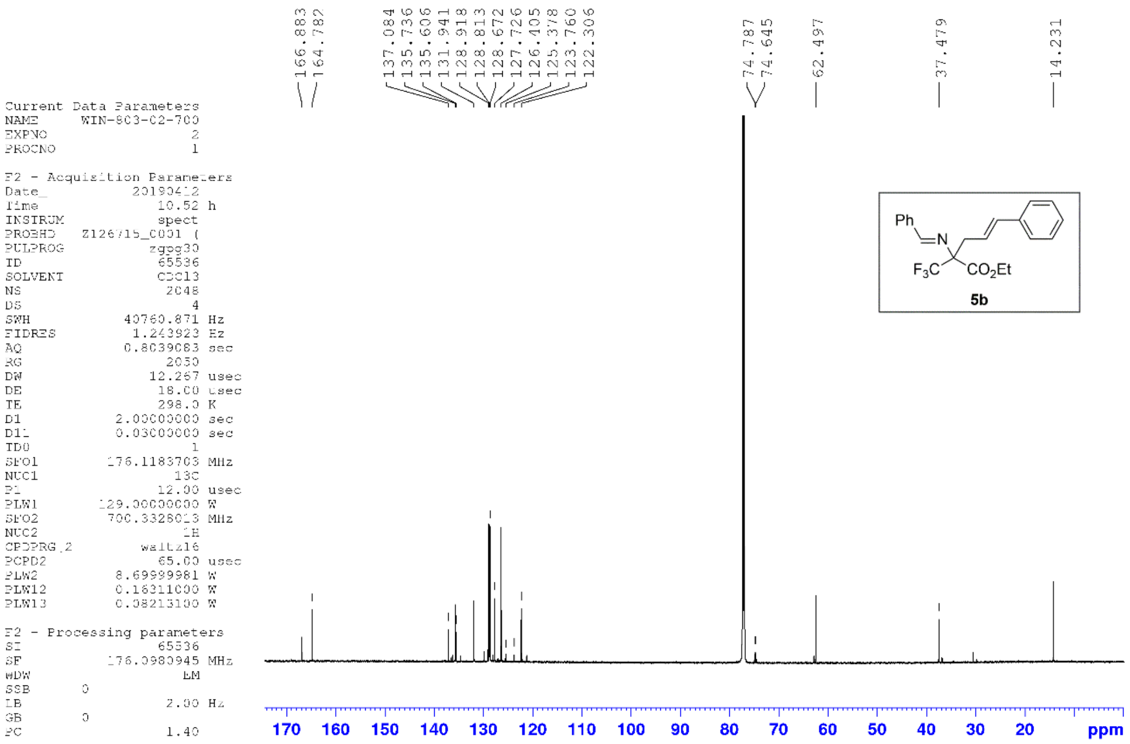
Ph
N
F3C
CO2Et
5b
ppm

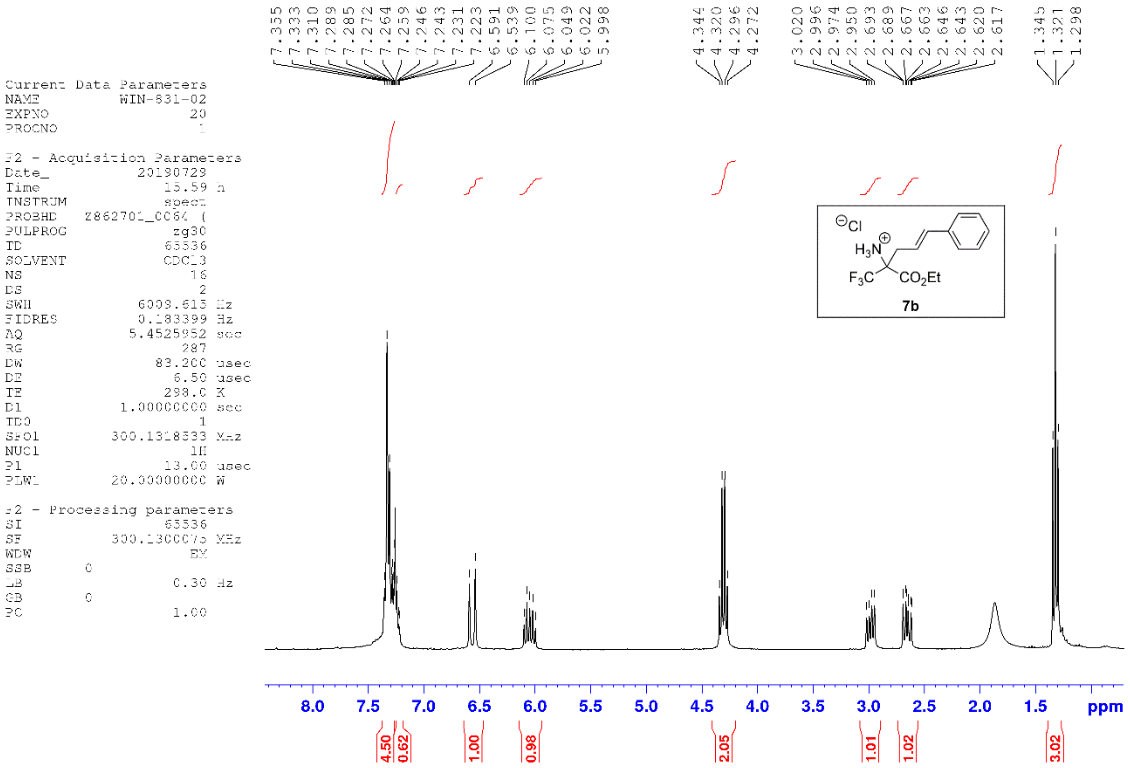
Cl
H3N
F3C
CO2Et
7b
ppm

```
Current Data Parameters
NAME          WIN-831-02
EXPNO                 11
PROCNO                 1

F2 - Acquisition Parameters
Date_           20190415
Time               16.10 h
INSTRUM            spect
PROBHD   Z862701_0064 (
PULPROG           zgflqn
TD                131072
SOLVENT            CDCl3
NS                    16
DS                     4
SWH            66964.289 Hz
FIDRES          1.021794 Hz
AQ             0.9786710 sec
RG                   645
DW                 7.467 usec
DE                  6.50 usec
TE                 298.0 K
D1            1.00000000 sec
TD0                    1
SFO1         282.3761148 MHz
NUC1                 19F
P1                  8.60 usec
PLW1         19.99900055 W

F2 - Processing parameters
SI                 65536
SF           282.4049249 MHz
WDW                   EM
SSB      0
LB                  0.30 Hz
GB       0
PC                  1.00
```

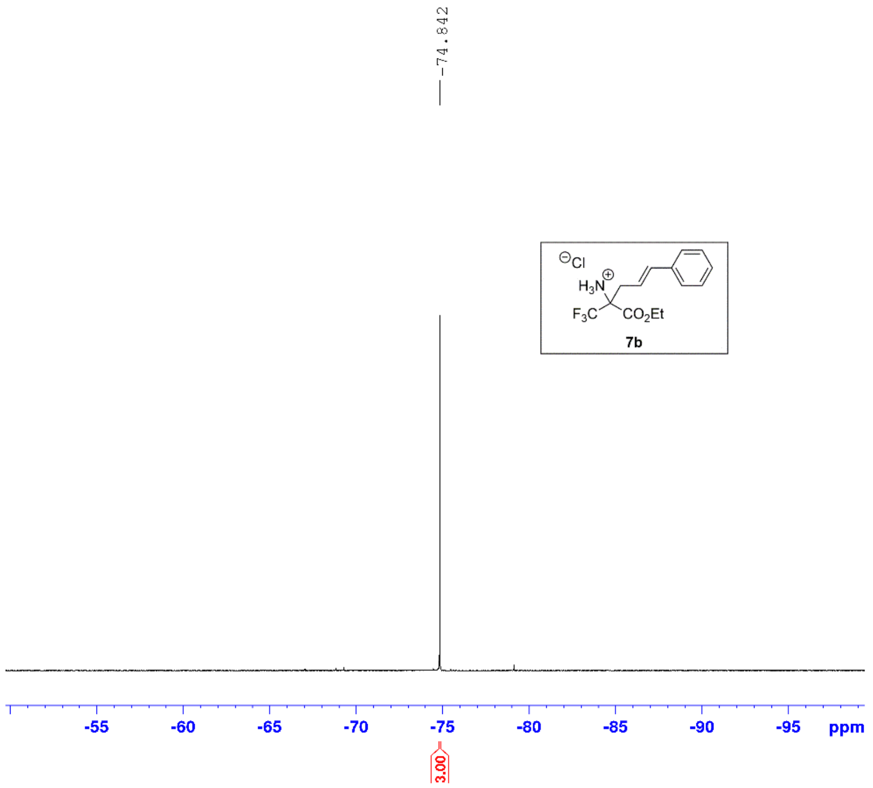


```
Current Data Parameters
NAME          WIN-831-02
EXPNO                 21
PROCNO                 1

F2 - Acquisition Parameters
Date_           20190729
Time               23.34 h
INSTRUM            spect
PROBHD   Z862701_0064 (
PULPROG           zgpg30
TD                 65536
SOLVENT            CDCl3
NS                  2048
DS                     4
SWH            18028.846 Hz
FIDRES          0.550197 Hz
AQ             1.8175317 sec
RG                  2050
DW                27.733 usec
DE                 27.73 usec
TE                 298.0 K
D1            2.00000000 sec
D11           0.03000000 sec
TD0                    1
SFO1          75.4752949 MHz
NUC1                 13C
P1                  8.88 usec
PLW1         50.00000000 W
SFO2         300.1312005 MHz
NUC2                  1H
CPDPRG[2          waltz16
PCPD2              90.00 usec
PLW2         20.00000000 W
PLW12         0.41727999 W
PLW13         0.20988999 W

F2 - Processing parameters
SI                 32768
SF            75.4677385 MHz
WDW                   EM
SSB      0
LB                  1.00 Hz
GB       0
PC                  1.40
```

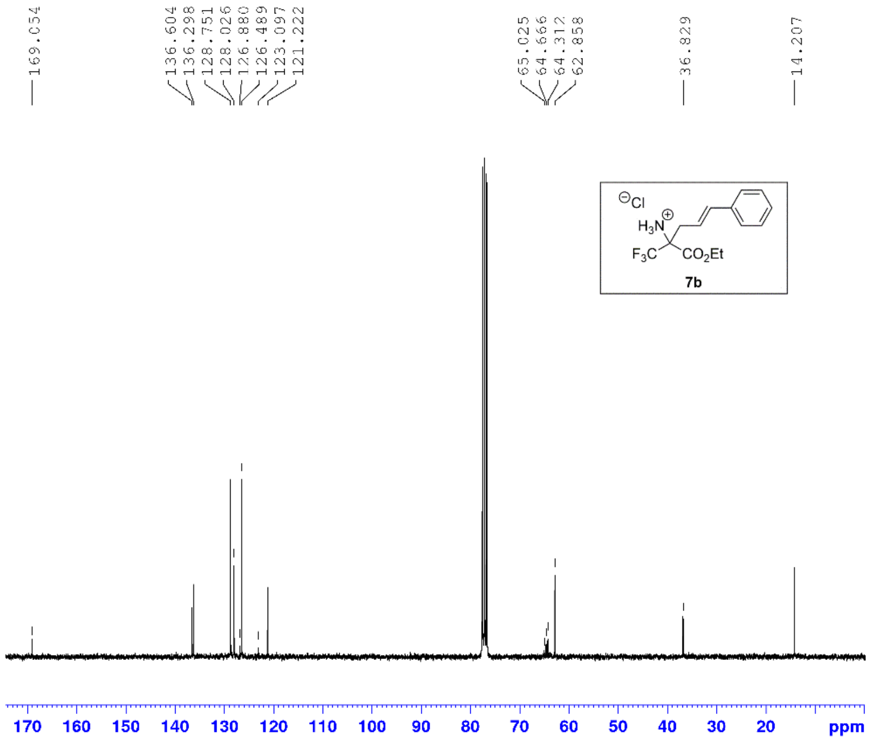

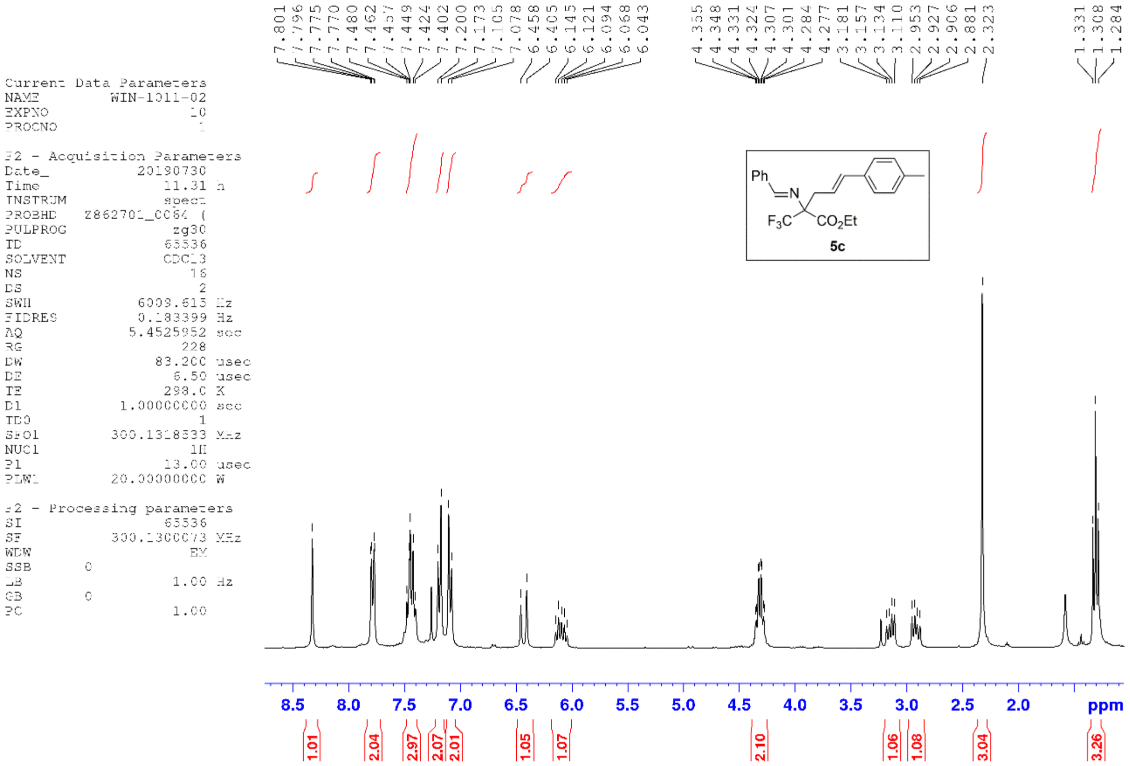
Current Data Parameters
NAME WIN-1011-02
EXPNO 10
PROCNO 1
F2 - Acquisition Parameters
Date_ 20190730
Time 11.31 h
INSTRUM spect
PROBHD Z862701_0064 (
PULPROG zg30
TD 65536
SOLVENT CDCl3
NS 16
DS 2
SWH 6009.615 Hz
FIDRES 0.183399 Hz
AQ 5.4525952 sec
RG 228
DW 83.200 usec
DE 6.50 usec
TE 298.0 K
D1 1.00000000 sec
TD0 1
SFO1 300.1318533 MHz
NUC1 1H
P1 13.00 usec
PLW1 20.00000000 W
F2 - Processing parameters
SI 65536
SF 300.1300073 MHz
WDW EM
SSB 0
LB 1.00 Hz
GB 0
PC 1.00
7.801 7.796 7.775 7.770 7.480 7.462 7.457 7.449 7.424 7.402 7.200 7.173 7.105 7.078 6.458 6.405 6.145 6.121 6.094 6.068 6.043
4.355 4.348 4.331 4.324 4.307 4.301 4.284 4.277 3.181 3.157 3.134 3.110 2.953 2.927 2.906 2.881 2.323
1.331 1.308 1.284
Ph
N
F3C
CO2Et
5c
8.5 8.0 7.5 7.0 6.5 6.0 5.5 5.0 4.5 4.0 3.5 3.0 2.5 2.0 ppm
1.01 2.04 2.97 2.07 2.01 1.05 1.07 2.10 1.06 1.08 3.04 3.26

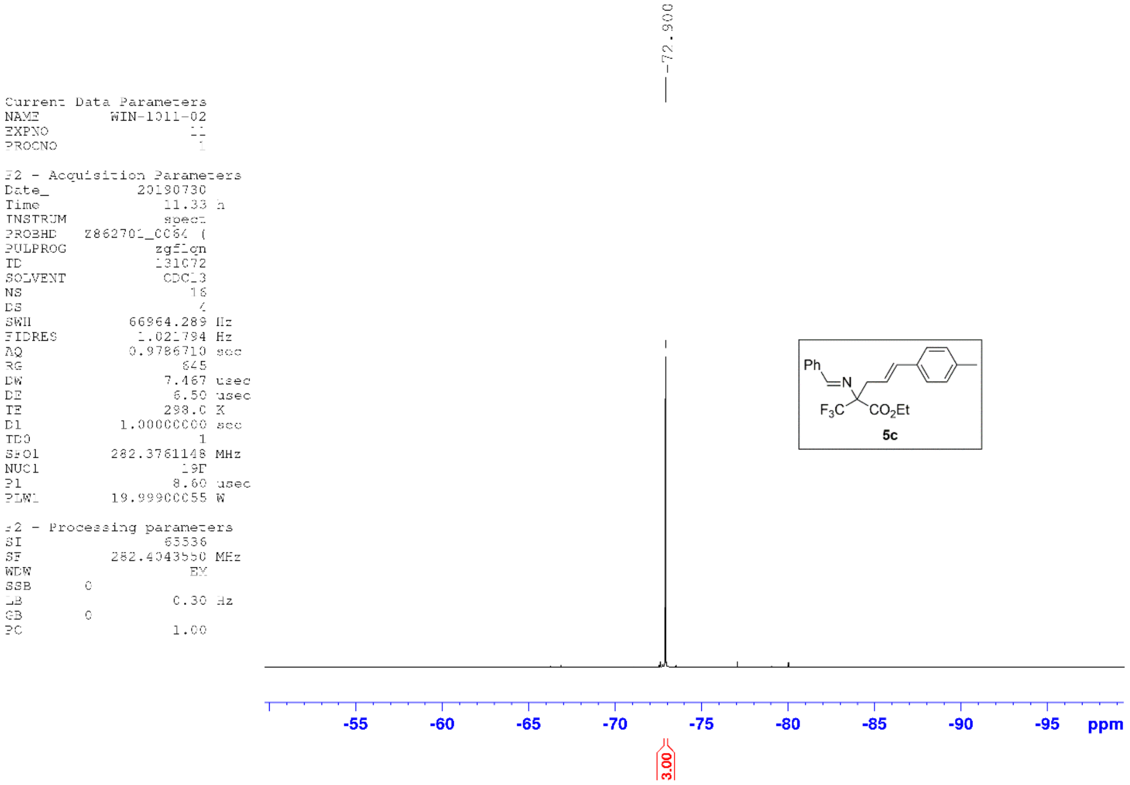
Current Data Parameters
NAME WIN-1011-02
EXPNO 11
PROCNO 1
F2 - Acquisition Parameters
Date_ 20190730
Time 11.33 h
INSTRUM spect
PROBHD Z862701_0064 (
PULPROG zgflqn
TD 131072
SOLVENT CDCl3
NS 16
DS 4
SWH 66964.289 Hz
FIDRES 1.021794 Hz
AQ 0.9786710 sec
RG 645
DW 7.467 usec
DE 6.50 usec
TE 298.0 K
D1 1.00000000 sec
TD0 1
SFO1 282.3761148 MHz
NUC1 19F
P1 8.60 usec
PLW1 19.99900055 W
F2 - Processing parameters
SI 65536
SF 282.4043550 MHz
WDW EM
SSB 0
LB 0.30 Hz
GB 0
PC 1.00
-72.900
Ph
N
F3C
CO2Et
5c
-55 -60 -65 -70 -75 -80 -85 -90 -95 ppm
3.00

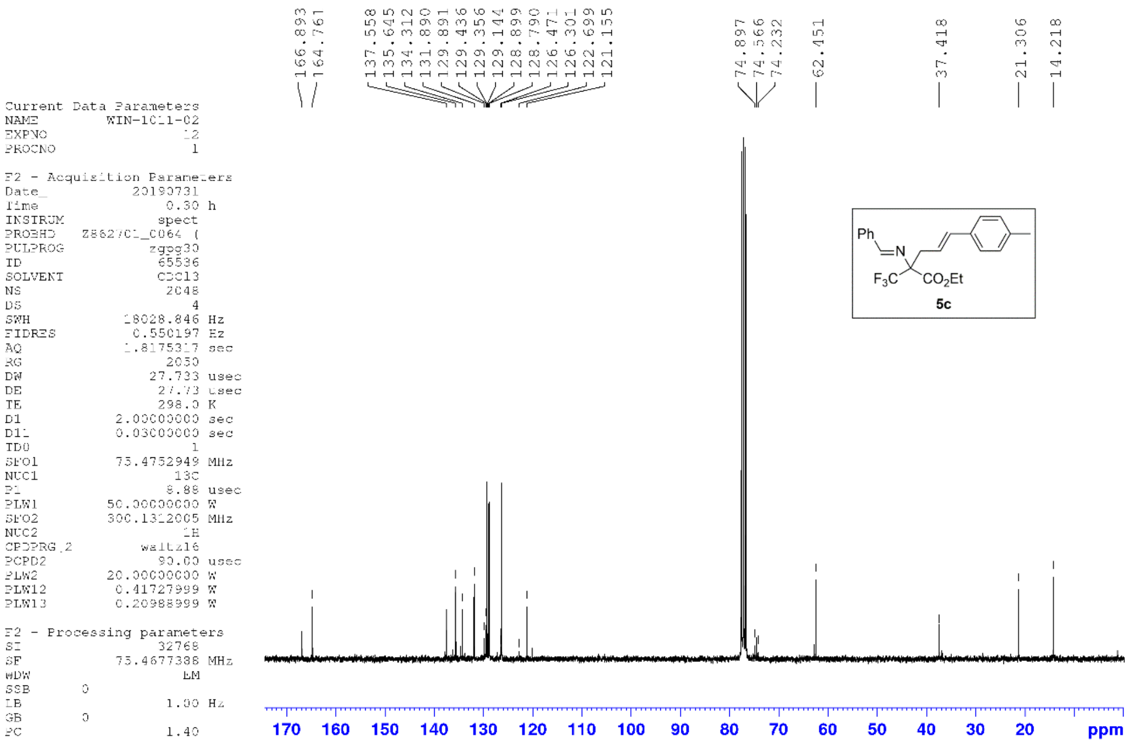
166.893
164.761
137.558
135.645
134.312
131.891
129.891
129.436
129.356
129.144
128.899
128.790
126.471
126.301
122.699
121.155
74.897
74.566
74.232
62.451
37.418
21.306
14.218
Current Data Parameters
NAME WIN-1011-02
EXPNO 12
PROCNO 1
F2 - Acquisition Parameters
Date_ 20190731
Time 0.30 h
INSTRUM spect
PROBHD Z862701_0064 (
PULPROG zgpg30
TD 65536
SOLVENT CDCl3
NS 2048
DS 4
SWH 18028.846 Hz
FIDRES 0.550197 Hz
AQ 1.8175317 sec
RG 2050
DW 27.733 usec
DE 27.73 usec
TE 298.0 K
D1 2.00000000 sec
D11 0.03000000 sec
TD0 1
SFO1 75.4752949 MHz
NUC1 13C
P1 8.88 usec
PLW1 50.00000000 W
SFO2 300.1312005 MHz
NUC2 1H
CPDPRG_2 waltz16
PCPD2 90.00 usec
PLW2 20.00000000 W
PLW12 0.41727999 W
PLW13 0.20988999 W
F2 - Processing parameters
SI 32768
SF 75.4677388 MHz
WDW EM
SSB 0
LB 1.00 Hz
GB 0
PC 1.40
Ph
N
F3C
CO2Et
5c
170 160 150 140 130 120 110 100 90 80 70 60 50 40 30 20 ppm

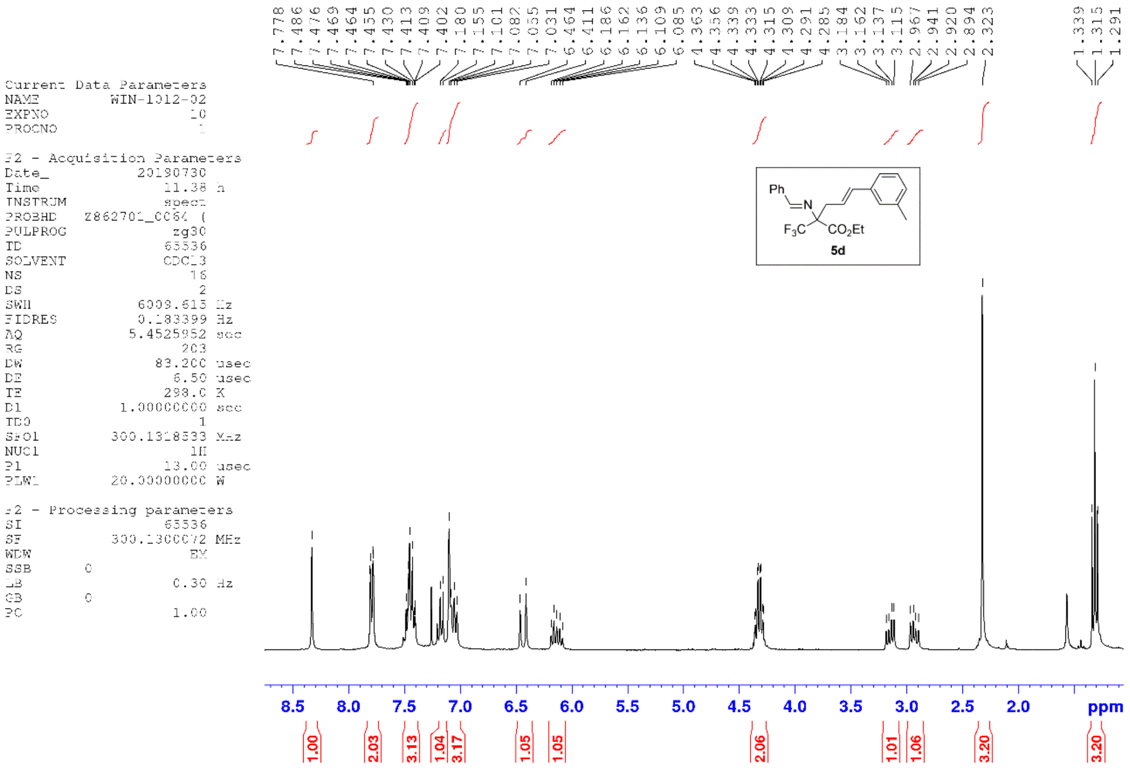
7.778
7.486
7.476
7.469
7.464
7.455
7.430
7.413
7.409
7.402
7.180
7.155
7.101
7.082
7.055
7.031
6.464
6.411
6.186
6.162
6.136
6.109
6.085
4.363
4.356
4.339
4.333
4.315
4.309
4.291
4.285
3.184
3.162
3.137
3.115
2.967
2.941
2.920
2.894
2.323
1.339
1.315
1.291
Current Data Parameters
NAME WIN-1012-02
EXPNO 10
PROCNO 1
F2 - Acquisition Parameters
Date_ 20190730
Time 11.38 h
INSTRUM spect
PROBHD Z862701_0064 (
PULPROG zg30
TD 65536
SOLVENT CDCl3
NS 16
DS 2
SWH 6009.615 Hz
FIDRES 0.183399 Hz
AQ 5.4525952 sec
RG 203
DW 83.200 usec
DE 6.50 usec
TE 298.0 K
D1 1.00000000 sec
TD0 1
SFO1 300.1318533 MHz
NUC1 1H
P1 13.00 usec
PLW1 20.00000000 W
F2 - Processing parameters
SI 65536
SF 300.1300072 MHz
WDW EM
SSB 0
LB 0.30 Hz
GB 0
PC 1.00
Ph
N
F3C
CO2Et
5d
8.5 8.0 7.5 7.0 6.5 6.0 5.5 5.0 4.5 4.0 3.5 3.0 2.5 2.0 ppm
1.00
2.03
3.13
1.04
3.17
1.05
1.05
2.06
1.01
1.06
3.20
3.20

```
Current Data Parameters
NAME          WIN-1012-02
EXPNO                  11
PROCNO                  1

F2 - Acquisition Parameters
Date_            20190730
Time                11.39 h
INSTRUM             spect
PROBHD   Z862701_0064 (
PULPROG           zgflqn
TD                131072
SOLVENT            CDCl3
NS                    16
DS                     4
SWH            66964.289 Hz
FIDRES          1.021794 Hz
AQ             0.9786710 sec
RG                   645
DW                 7.467 usec
DE                  6.50 usec
TE                 298.0 K
D1            1.00000000 sec
TD0                    1
SFO1         282.3781148 MHz
NUC1                 19F
P1                  8.60 usec
PLW1         19.99900055 W

F2 - Processing parameters
SI                 65536
SF           282.4043550 MHz
WDW                   EM
SSB      0
LB                  0.30 Hz
GB       0
PC                  1.00
```

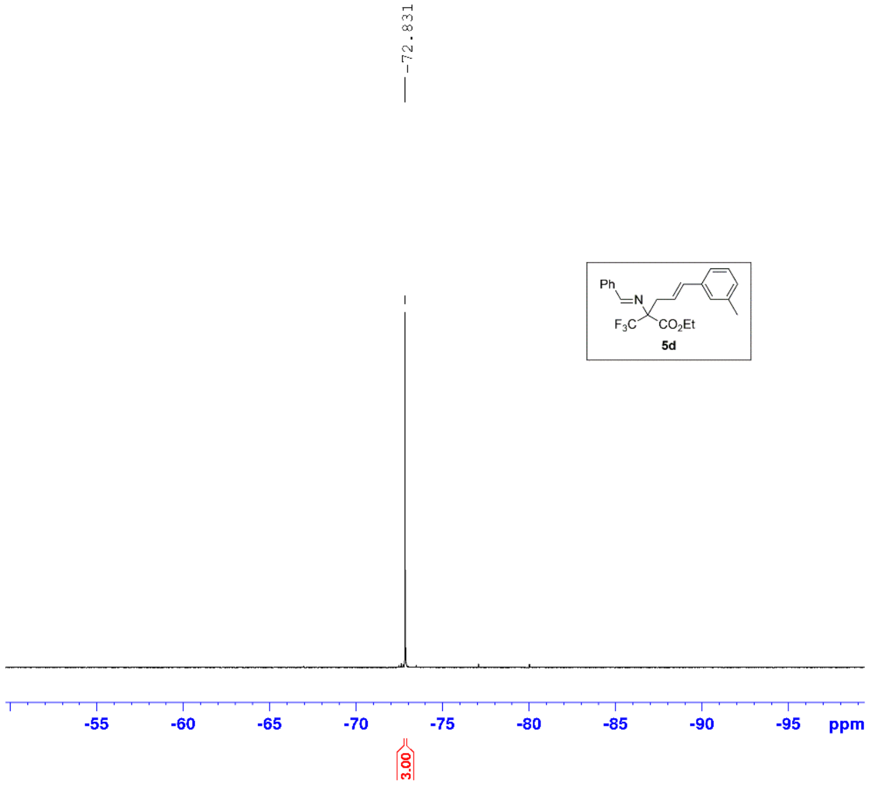


```
Current Data Parameters
NAME          WIN-1012-02
EXPNO                  12
PROCNO                  1

F2 - Acquisition Parameters
Date_            20190731
Time                 5.40 h
INSTRUM             spect
PROBHD   Z862701_0064 (
PULPROG           zgpg30
TD                 65536
SOLVENT            CDCl3
NS                  2048
DS                     4
SWH            18028.846 Hz
FIDRES          0.550197 Hz
AQ             1.8175317 sec
RG                  2050
DW                27.733 usec
DE                 27.73 usec
TE                 298.3 K
D1            2.00000000 sec
D11           0.03000000 sec
TD0                    1
SFO1          75.4752949 MHz
NUC1                 13C
P1                  8.88 usec
PLW1         50.00000000 W
SFO2         300.1312005 MHz
NUC2                  1H
CPDPRG[2         waltz16
PCPD2              90.00 usec
PLW2         20.00000000 W
PLW12         0.41727999 W
PLW13         0.20988999 W

F2 - Processing parameters
SI                 32768
SF            75.4677395 MHz
WDW                   EM
SSB      0
LB                  1.00 Hz
GB       0
PC                  1.40
```

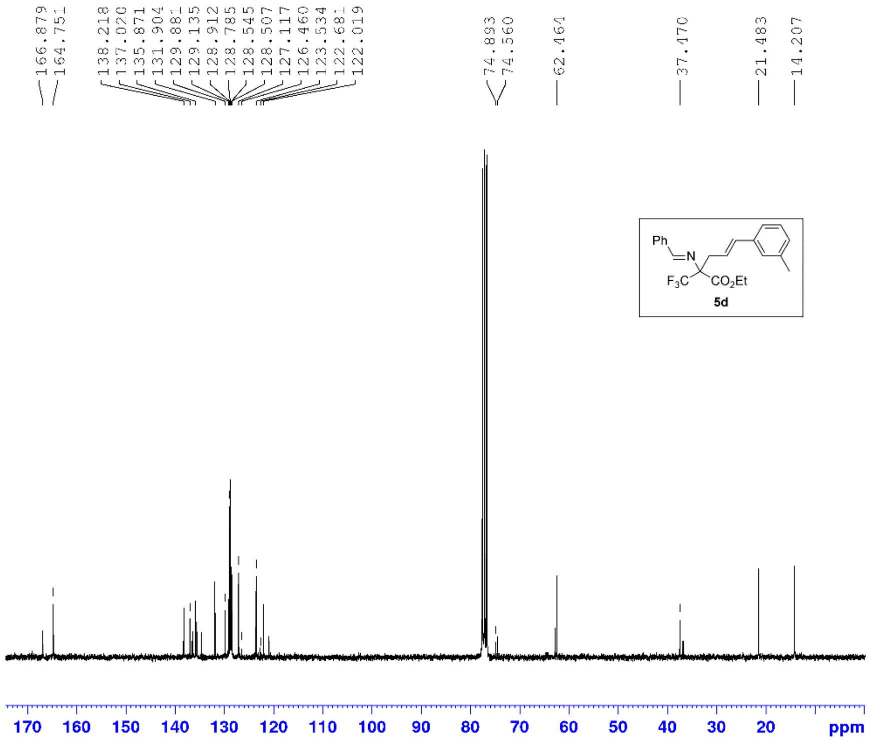

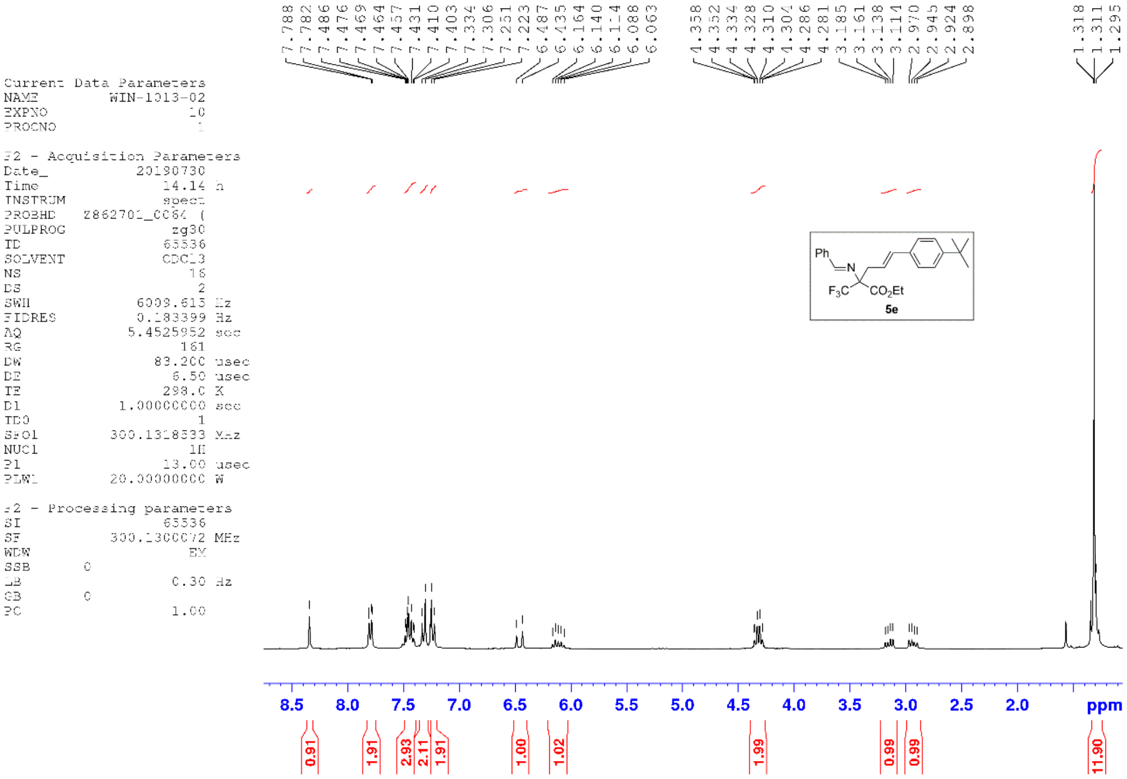

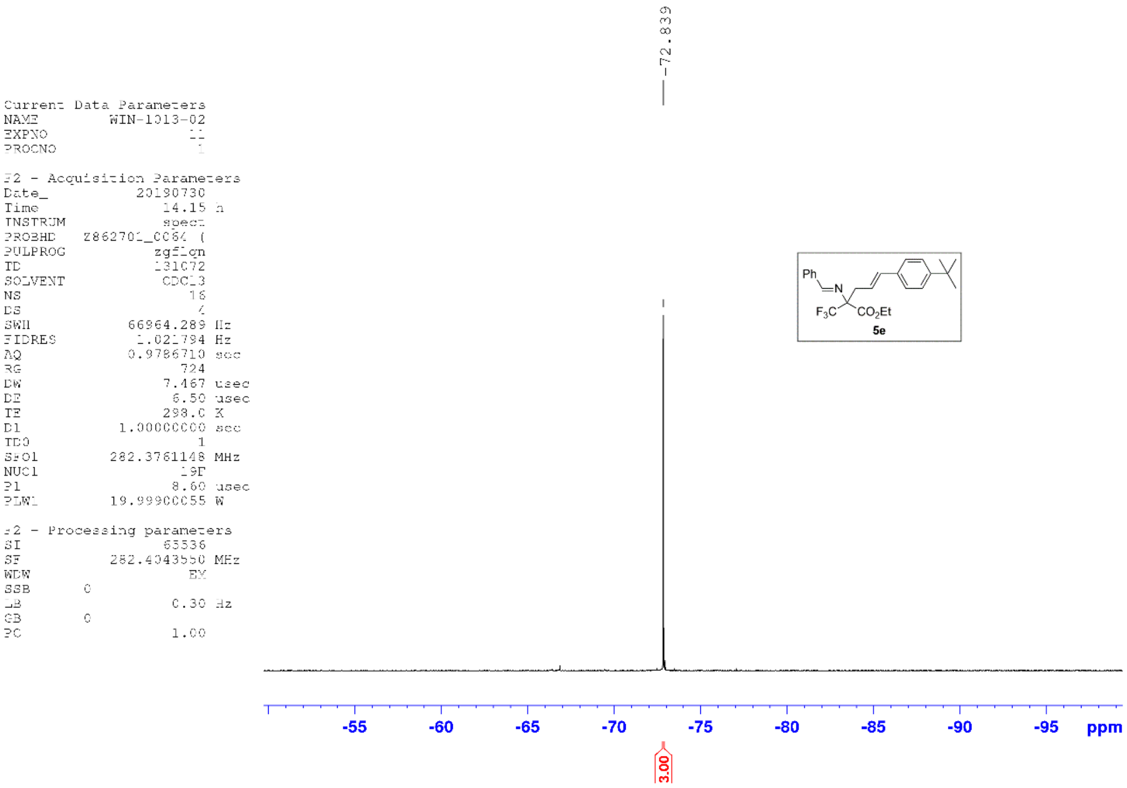

Current Data Parameters
NAME WIN-1013-02
EXPNO 12
PROCNO 1

F2 - Acquisition Parameters
Date_ 20190731
Time 22.23 h
INSTRUM spect
PROBHD Z862701_0064 (
PULPROG zgpg30
TD 65536
SOLVENT CDCl3
NS 2048
DS 4
SWH 18028.846 Hz
FIDRES 0.550197 Hz
AQ 1.8175317 sec
RG 2050
DW 27.733 usec
DE 27.73 usec
TE 298.0 K
D1 2.00000000 sec
D11 0.03000000 sec
TD0 1
SFO1 75.4752949 MHz
NUC1 13C
P1 8.88 usec
PLW1 50.00000000 W
SFO2 300.1312005 MHz
NUC2 1H
CPDPRG[2 waltz16
PCPD2 90.00 usec
PLW2 20.00000000 W
PLW12 0.41727999 W
PLW13 0.20988999 W

F2 - Processing parameters
SI 32768
SF 75.4677394 MHz
WDW EM
SSB 0
LB 1.00 Hz
GB 0
PC 1.40

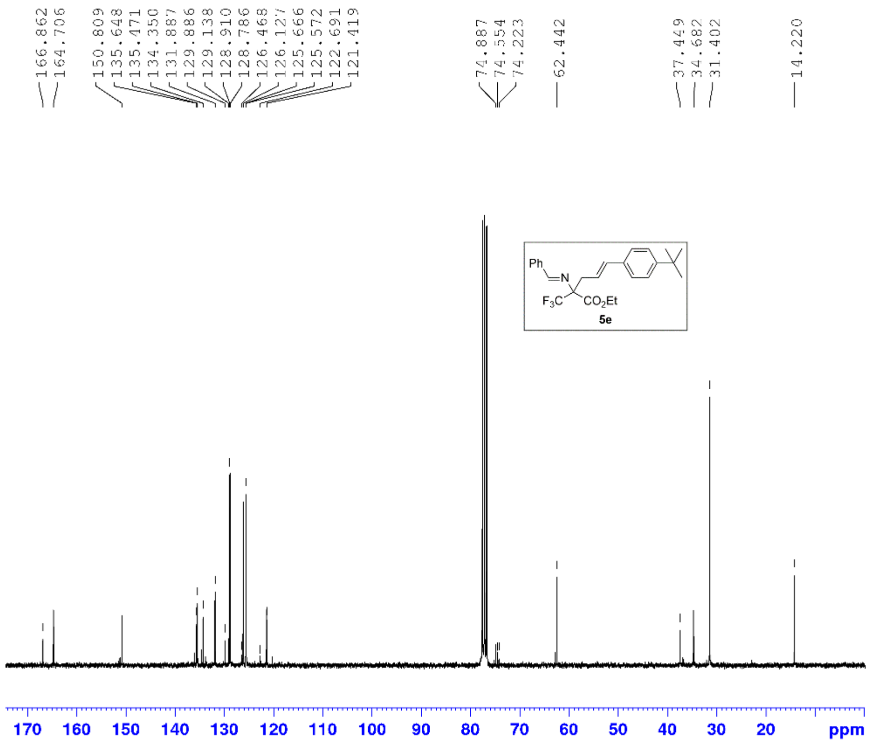

Current Data Parameters
NAME WIN-1043-02
EXPNO 10
PROCNO 1

F2 - Acquisition Parameters
Date_ 20190808
Time 15.37 h
INSTRUM spect
PROBHD Z862701_0064 (
PULPROG zg30
TD 65536
SOLVENT CDCl3
NS 16
DS 2
SWH 6009.615 Hz
FIDRES 0.183399 Hz
AQ 5.4525952 sec
RG 256
DW 83.200 usec
DE 6.50 usec
TE 298.0 K
D1 1.00000000 sec
TD0 1
SFO1 300.1318533 MHz
NUC1 1H
P1 13.00 usec
PLW1 20.00000000 W

F2 - Processing parameters
SI 65536
SF 300.1300079 MHz
WDW EM
SSB 0
LB 0.30 Hz
GB 0
PC 1.00

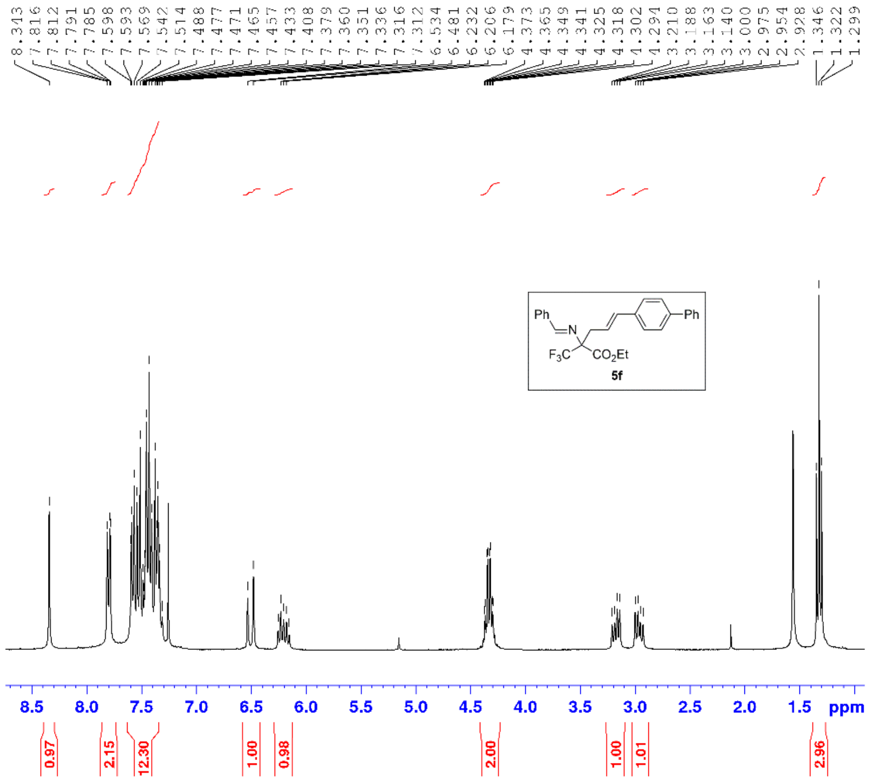

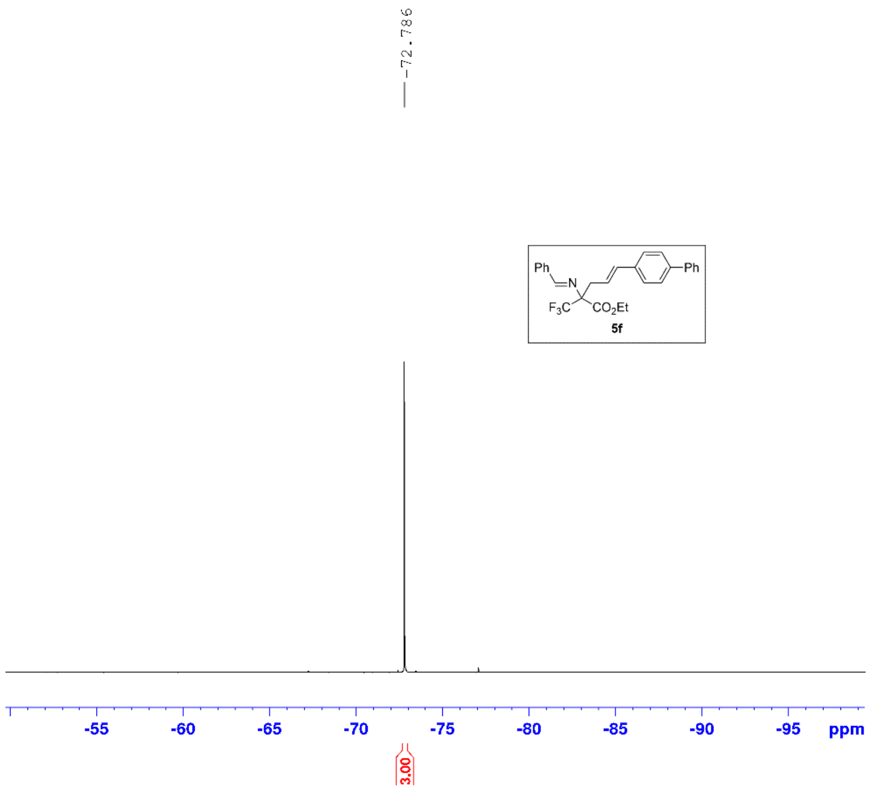

Current Data Parameters
NAME WIN-1043-02
EXPNO 11
PROCNO 1

F2 - Acquisition Parameters
Date_ 20190808
Time 15.38 h
INSTRUM spect
PROBHD Z862701_0064 (
PULPROG zgflqn
TD 131072
SOLVENT CDCl3
NS 16
DS 4
SWH 66964.289 Hz
FIDRES 1.021794 Hz
AQ 0.9786710 sec
RG 724
DW 7.467 usec
DE 6.50 usec
TE 298.0 K
D1 1.00000000 sec
TD0 1
SFO1 282.3761148 MHz
NUC1 19F
P1 8.60 usec
PLW1 19.99900055 W

F2 - Processing parameters
SI 65536
SF 282.4043550 MHz
WDW EM
SSB 0
LB 0.30 Hz
GB 0
PC 1.00

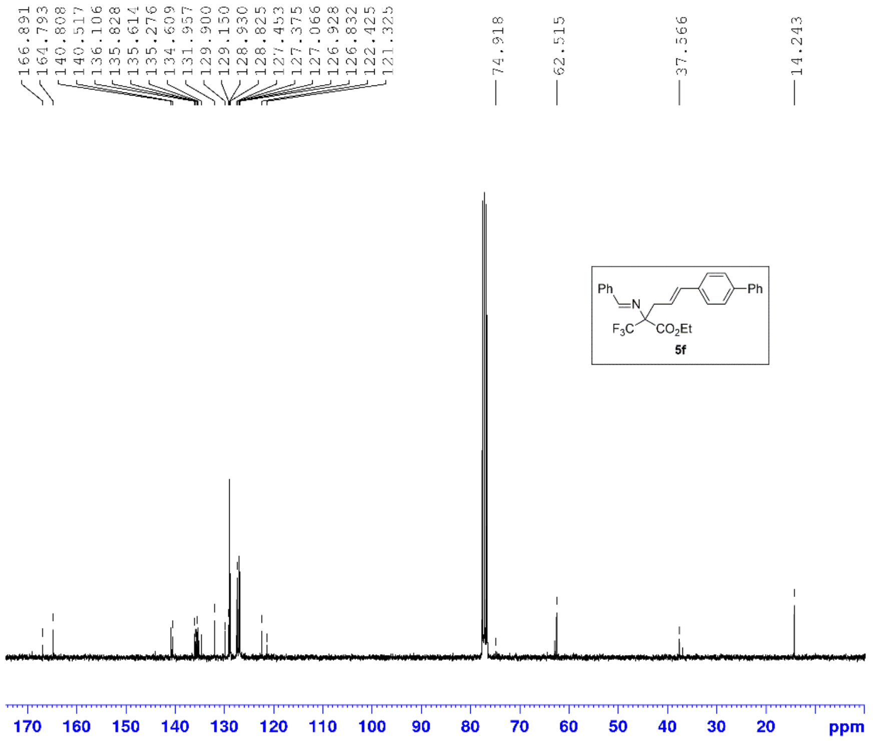

Current Data Parameters
NAME WIN-1043-02
EXPNO 12
PROCNO 1

F2 - Acquisition Parameters
Date_ 20190809
Time 5.59 h
INSTRUM spect
PROBHD Z862701_0064 (
PULPROG zgpg30
TD 65536
SOLVENT CDCl3
NS 2048
DS 4
SWH 18028.846 Hz
FIDRES 0.550197 Hz
AQ 1.8175317 sec
RG 2050
DW 27.733 usec
DE 27.73 usec
TE 298.3 K
D1 2.00000000 sec
D11 0.03000000 sec
TD0 1
SFO1 75.4752949 MHz
NUC1 13C
P1 8.88 usec
PLW1 50.00000000 W
SFO2 300.1312005 MHz
NUC2 1H
CPDPRG[2 waltz16
PCPD2 90.00 usec
PLW2 20.00000000 W
PLW12 0.41727999 W
PLW13 0.20988999 W

F2 - Processing parameters
SI 32768
SF 75.4677383 MHz
WDW EM
SSB 0
LB 1.00 Hz
GB 0
PC 1.40

Current Data Parameters
NAME WIN-1042-02
EXPNO 10
PROCNO 1

F2 - Acquisition Parameters
Date_ 20190807
Time 13.13 h
INSTRUM spect
PROBHD Z862701_0064 (
PULPROG zg30
TD 65536
SOLVENT CDCl3
NS 16
DS 2
SWH 6009.615 Hz
FIDRES 0.183399 Hz
AQ 5.4525952 sec
RG 181
DW 83.200 usec
DE 6.50 usec
TE 298.0 K
D1 1.00000000 sec
TD0 1
SFO1 300.1318533 MHz
NUC1 1H
P1 13.00 usec
PLW1 20.00000000 W

F2 - Processing parameters
SI 65536
SF 300.1300069 MHz
WDW EM
SSB 0
LB 0.30 Hz
GB 0
PC 1.00

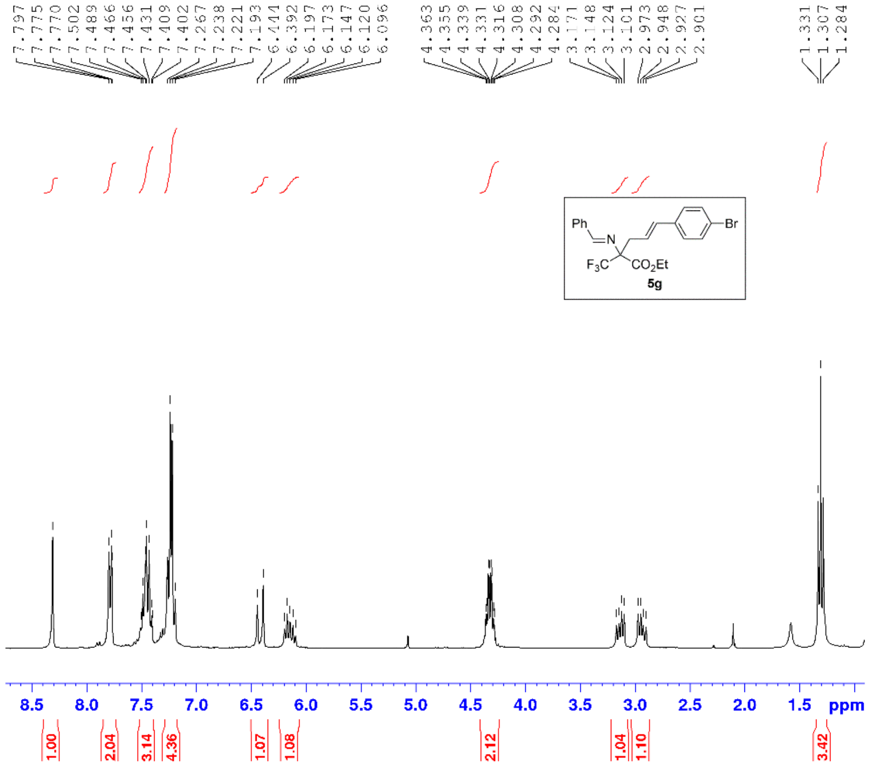

Current Data Parameters
NAME WIN-1042-02
EXPNO 11
PROCNO 1

F2 - Acquisition Parameters
Date_ 20190807
Time 13.15 h
INSTRUM spect
PROBHD Z862701_0064 (
PULPROG zgflqn
TD 131072
SOLVENT CDCl3
NS 16
DS 4
SWH 66964.289 Hz
FIDRES 1.021794 Hz
AQ 0.9786710 sec
RG 645
DW 7.467 usec
DE 6.50 usec
TE 298.0 K
D1 1.00000000 sec
TD0 1
SFO1 282.3761148 MHz
NUC1 19F
P1 8.60 usec
PLW1 19.99900055 W

F2 - Processing parameters
SI 65536
SF 282.4043550 MHz
WDW EM
SSB 0
LB 0.30 Hz
GB 0
PC 1.00

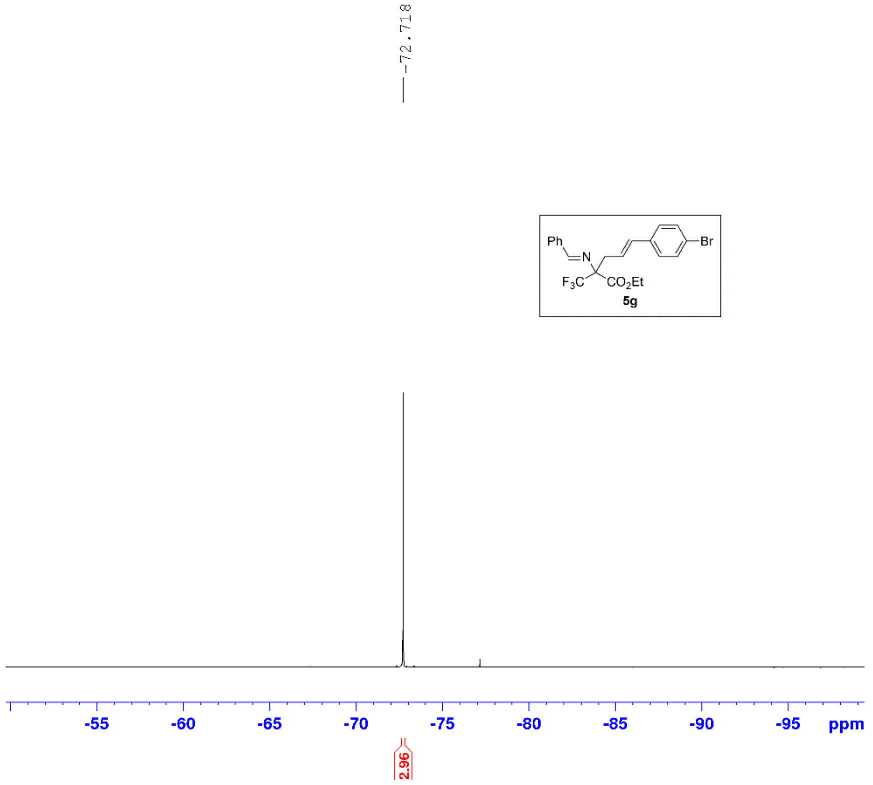

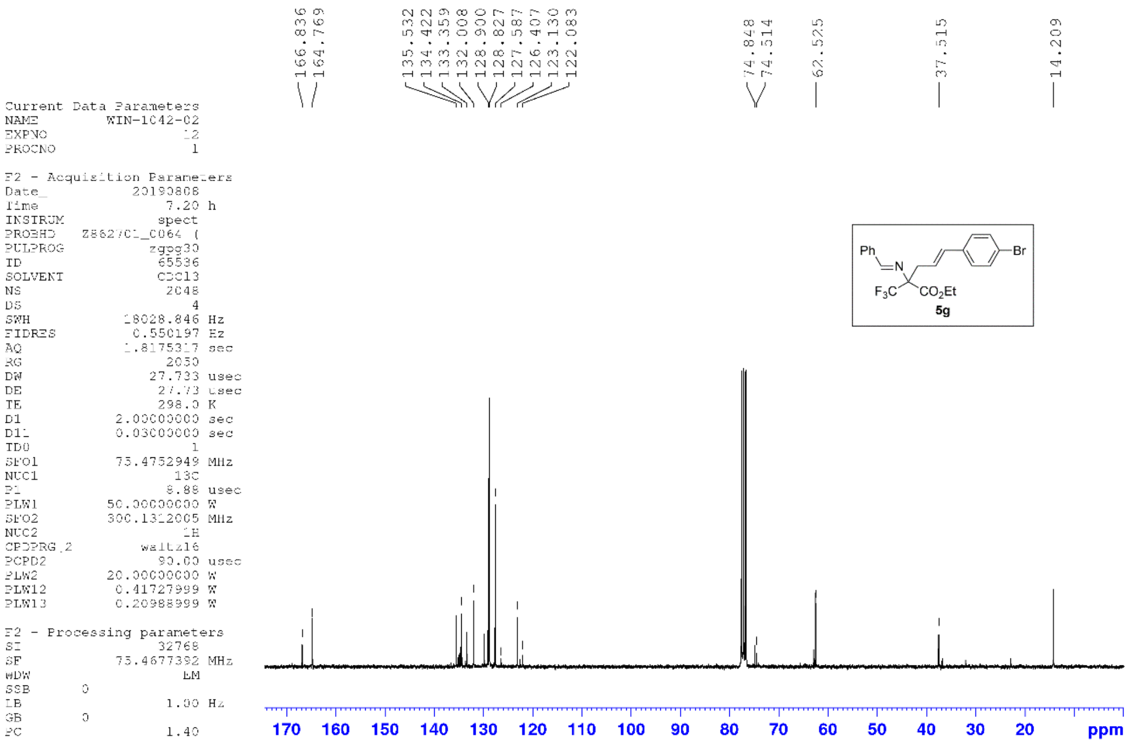
166.836
164.769
135.532
134.422
133.359
132.008
128.900
128.827
127.587
126.407
123.130
122.083
74.848
74.514
62.525
37.515
14.209
Current Data Parameters
NAME WIN-1042-02
EXPNO 12
PROCNO 1
F2 - Acquisition Parameters
Date_ 20190808
Time 7.20 h
INSTRUM spect
PROBHD Z862701_0064 (
PULPROG zgpg30
TD 65536
SOLVENT CDCl3
NS 2048
DS 4
SWH 18028.846 Hz
FIDRES 0.550197 Hz
AQ 1.8175317 sec
RG 2050
DW 27.733 usec
DE 27.73 usec
TE 298.0 K
D1 2.00000000 sec
D11 0.03000000 sec
TD0 1
SFO1 75.4752949 MHz
NUC1 13C
P1 8.88 usec
PLW1 50.00000000 W
SFO2 300.1312005 MHz
NUC2 1H
CPDPRG[2 waltz16
PCPD2 90.00 usec
PLW2 20.00000000 W
PLW12 0.41727999 W
PLW13 0.20988999 W
F2 - Processing parameters
SI 32768
SF 75.4677392 MHz
WDW EM
SSB 0
LB 1.00 Hz
GB 0
PC 1.40
Ph
N
Br
F3C
CO2Et
5g
170 160 150 140 130 120 110 100 90 80 70 60 50 40 30 20 ppm

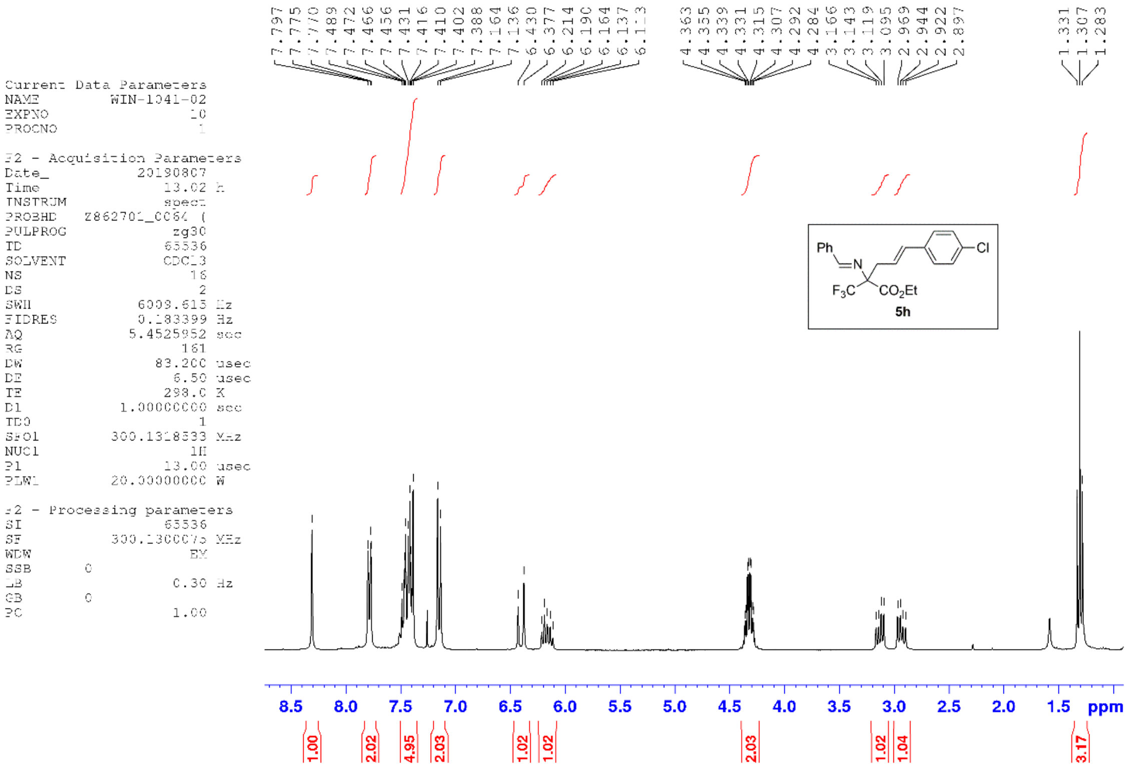
7.797
7.775
7.770
7.489
7.472
7.466
7.456
7.431
7.416
7.410
7.402
7.388
7.164
7.136
6.430
6.377
6.214
6.190
6.164
6.137
6.113
4.363
4.355
4.339
4.331
4.315
4.307
4.292
4.284
3.166
3.143
3.119
3.095
2.969
2.944
2.922
2.897
1.331
1.307
1.283
Current Data Parameters
NAME WIN-1041-02
EXPNO 10
PROCNO 1
F2 - Acquisition Parameters
Date_ 20190807
Time 13.02 h
INSTRUM spect
PROBHD Z862701_0064 (
PULPROG zg30
TD 65536
SOLVENT CDCl3
NS 16
DS 2
SWH 6009.615 Hz
FIDRES 0.183399 Hz
AQ 5.4525952 sec
RG 161
DW 83.200 usec
DE 6.50 usec
TE 298.0 K
D1 1.00000000 sec
TD0 1
SFO1 300.1318533 MHz
NUC1 1H
P1 13.00 usec
PLW1 20.00000000 W
F2 - Processing parameters
SI 65536
SF 300.1300075 MHz
WDW EM
SSB 0
LB 0.30 Hz
GB 0
PC 1.00
Ph
N
Cl
F3C
CO2Et
5h
8.5 8.0 7.5 7.0 6.5 6.0 5.5 5.0 4.5 4.0 3.5 3.0 2.5 2.0 1.5 ppm
1.00
2.02
4.95
2.03
1.02
1.02
2.03
1.02
1.04
3.17

```
Current Data Parameters
NAME          WIN-1041-02
EXPNO                  11
PROCNO                  1

F2 - Acquisition Parameters
Date_            20190807
Time                13.03 h
INSTRUM             spect
PROBHD   Z862701_0064 (
PULPROG            zgflqn
TD                 131072
SOLVENT             CDCl3
NS                     16
DS                      4
SWH             66964.289 Hz
FIDRES           1.021794 Hz
AQ              0.9786710 sec
RG                   1440
DW                  7.467 usec
DE                   6.50 usec
TE                  298.0 K
D1             1.00000000 sec
TD0                     1
SFO1          282.3781148 MHz
NUC1                  19F
P1                   8.60 usec
PLW1          19.99900055 W

F2 - Processing parameters
SI                  65536
SF            282.4043550 MHz
WDW                    EM
SSB      0
LB                   0.30 Hz
GB       0
PC                   1.00
```

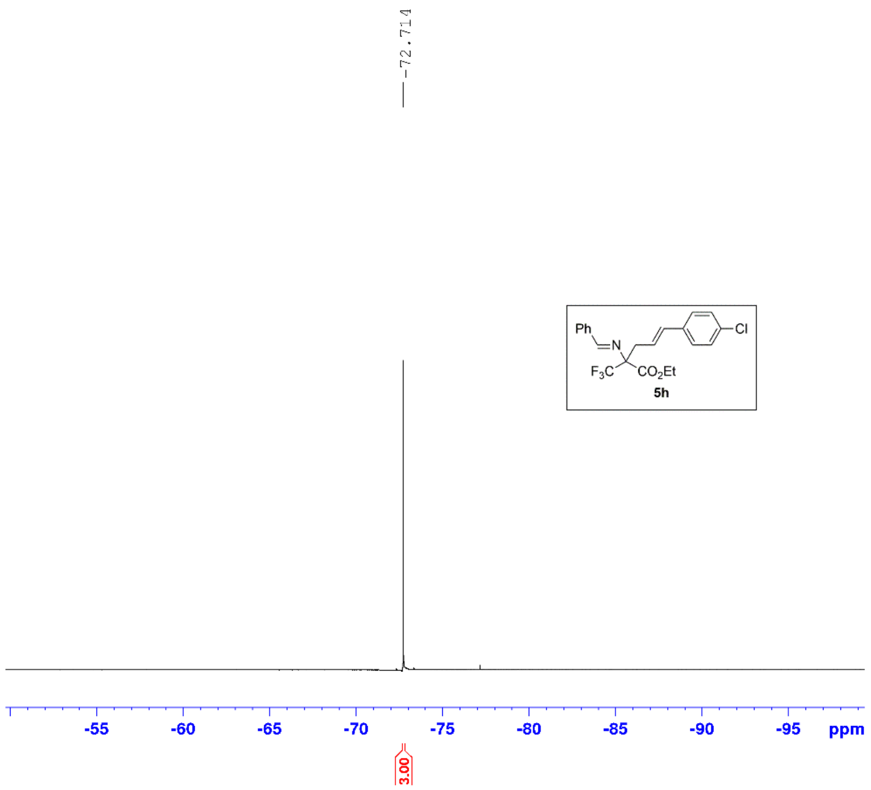


```
Current Data Parameters
NAME          WIN-1041-02
EXPNO                  12
PROCNO                  1

F2 - Acquisition Parameters
Date_            20190808
Time                 2.09 h
INSTRUM             spect
PROBHD   Z862701_0064 (
PULPROG           zgpg30
TD                  65536
SOLVENT             CDCl3
NS                   2048
DS                      4
SWH             18028.846 Hz
FIDRES           0.550197 Hz
AQ              1.8175317 sec
RG                   2050
DW                 27.733 usec
DE                  27.73 usec
TE                  298.3 K
D1             2.00000000 sec
D11            0.03000000 sec
TD0                     1
SFO1           75.4752949 MHz
NUC1                  13C
P1                   8.88 usec
PLW1          50.00000000 W
SFO2          300.1312005 MHz
NUC2                   1H
CPDPRG[2           waltz16
PCPD2               90.00 usec
PLW2          20.00000000 W
PLW12          0.41727999 W
PLW13          0.20988999 W

F2 - Processing parameters
SI                  32768
SF             75.4677392 MHz
WDW                    EM
SSB      0
LB                   1.00 Hz
GB       0
PC                   1.40
```

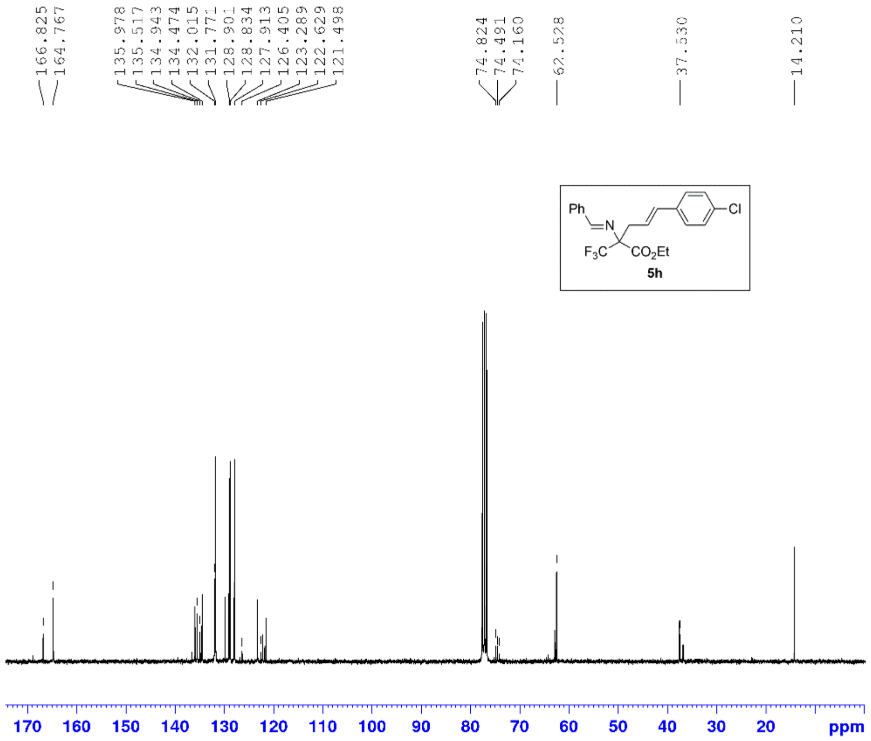

Current Data Parameters
NAME WIN-1044-02
EXPNO 10
PROCNO 1

F2 - Acquisition Parameters
Date_ 20190808
Time 15.43 h
INSTRUM spect
PROBHD Z862701_0064 (
PULPROG zg30
TD 65536
SOLVENT CDCl3
NS 16
DS 2
SWH 6009.615 Hz
FIDRES 0.183399 Hz
AQ 5.4525952 sec
RG 181
DW 83.200 usec
DE 6.50 usec
TE 298.0 K
D1 1.00000000 sec
TD0 1
SFO1 300.1318533 MHz
NUC1 1H
P1 13.00 usec
PLW1 20.00000000 W

F2 - Processing parameters
SI 65536
SF 300.1300072 MHz
WDW EM
SSB 0
LB 0.30 Hz
GB 0
PC 1.00

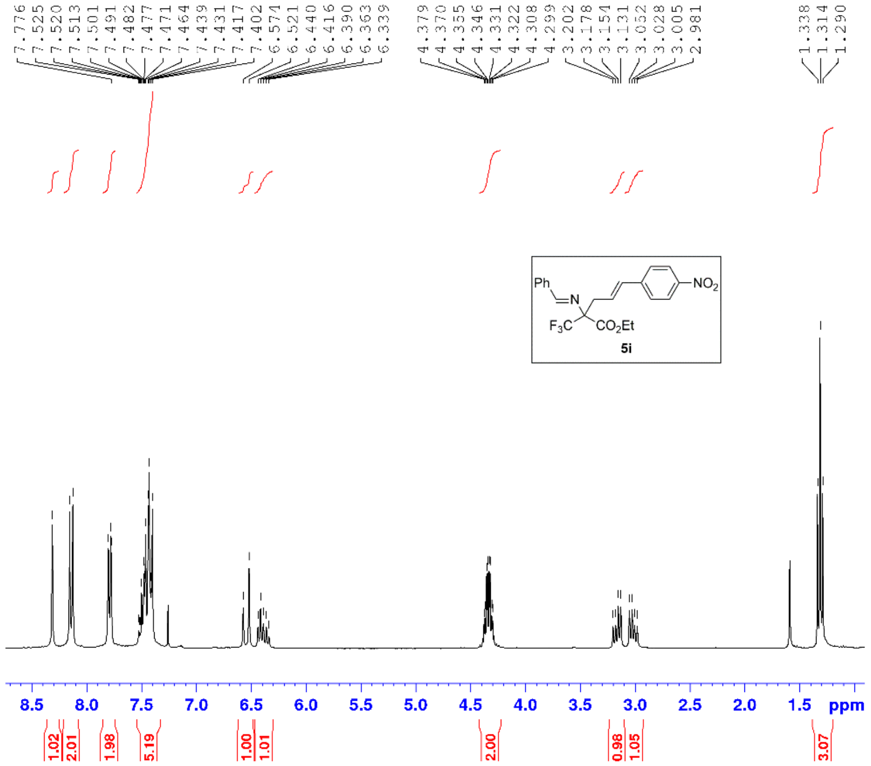

Current Data Parameters
NAME WIN-1044-02
EXPNO 11
PROCNO 1

F2 - Acquisition Parameters
Date_ 20190808
Time 15.44 h
INSTRUM spect
PROBHD Z862701_0064 (
PULPROG zgflqn
TD 131072
SOLVENT CDCl3
NS 16
DS 4
SWH 66964.289 Hz
FIDRES 1.021794 Hz
AQ 0.9786710 sec
RG 724
DW 7.467 usec
DE 6.50 usec
TE 298.0 K
D1 1.00000000 sec
TD0 1
SFO1 282.3761148 MHz
NUC1 19F
P1 8.60 usec
PLW1 19.99900055 W

F2 - Processing parameters
SI 65536
SF 282.4043550 MHz
WDW EM
SSB 0
LB 0.30 Hz
GB 0
PC 1.00

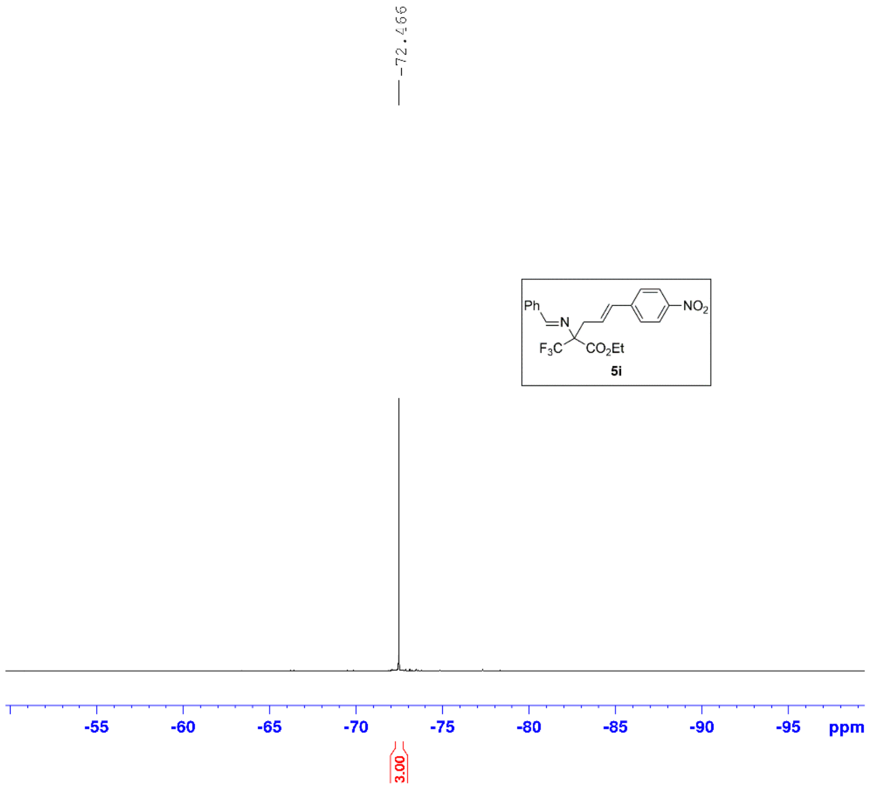

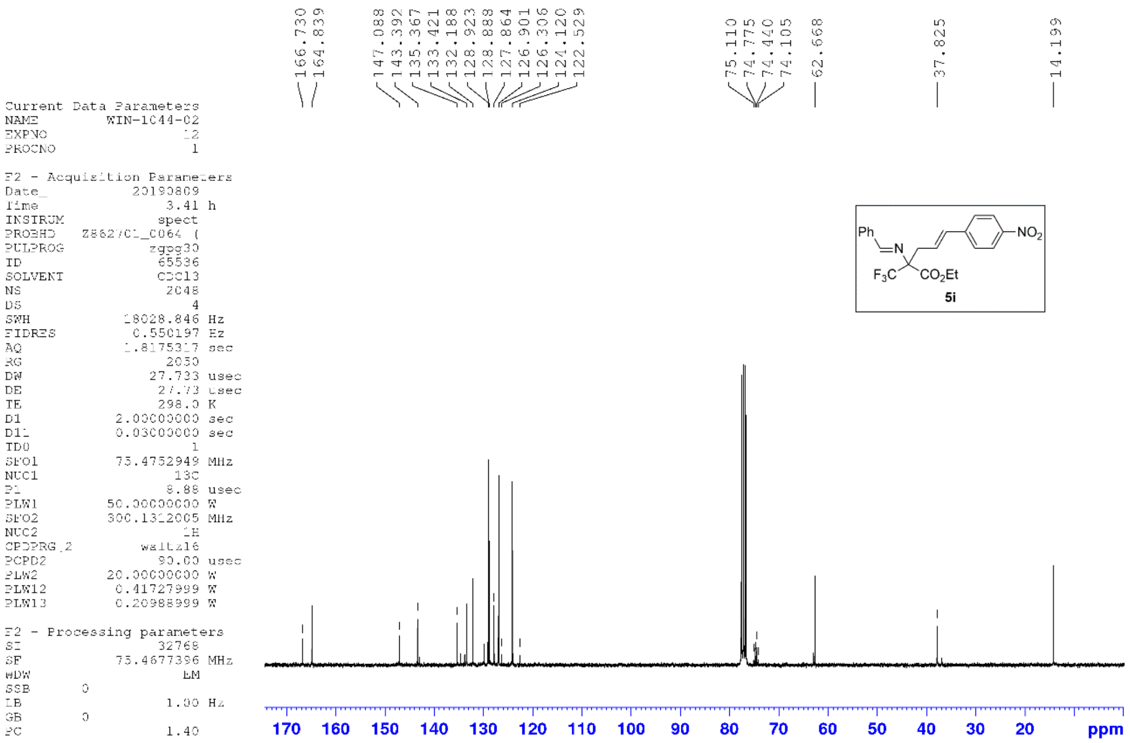
166.730
164.839
147.088
143.392
135.367
133.421
132.188
128.923
128.888
127.864
126.901
126.306
124.120
122.529
75.110
74.775
74.440
74.105
62.668
37.825
14.199
Current Data Parameters
NAME WIN-1044-02
EXPNO 12
PROCNO 1
F2 - Acquisition Parameters
Date_ 20190809
Time 3.41 h
INSTRUM spect
PROBHD Z862701_0064 (
PULPROG zgpg30
TD 65536
SOLVENT CDCl3
NS 2048
DS 4
SWH 18028.846 Hz
FIDRES 0.550197 Hz
AQ 1.8175317 sec
RG 2050
DW 27.733 usec
DE 27.73 usec
TE 298.0 K
D1 2.00000000 sec
D11 0.03000000 sec
TD0 1
SFO1 75.4752949 MHz
NUC1 13C
P1 8.88 usec
PLW1 50.00000000 W
SFO2 300.1312005 MHz
NUC2 1H
CPDPRG[2 waltz16
PCPD2 90.00 usec
PLW2 20.00000000 W
PLW12 0.41727999 W
PLW13 0.20988999 W
F2 - Processing parameters
SI 32768
SF 75.4677396 MHz
WDW EM
SSB 0
LB 1.00 Hz
GB 0
PC 1.40
Ph
N
NO2
F3C
CO2Et
5i
170 160 150 140 130 120 110 100 90 80 70 60 50 40 30 20 ppm

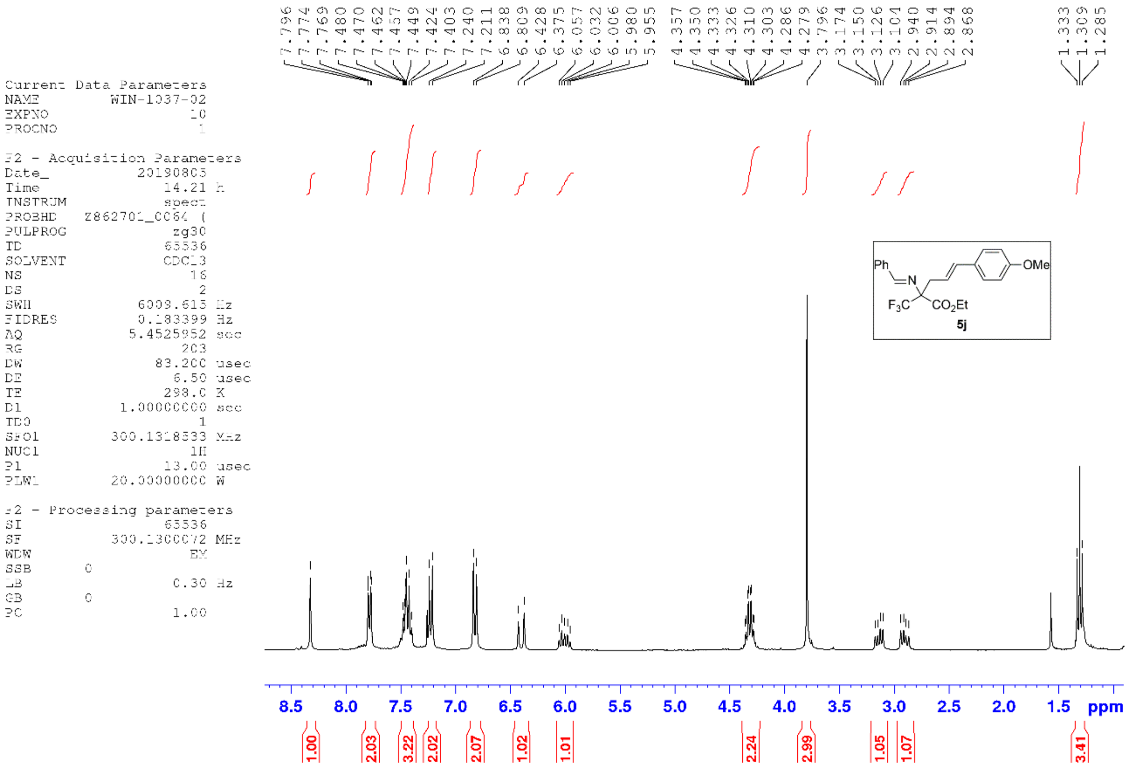
7.796
7.774
7.769
7.480
7.470
7.462
7.457
7.449
7.424
7.403
7.240
7.211
6.838
6.809
6.428
6.375
6.057
6.032
6.006
5.980
5.955
4.357
4.350
4.333
4.326
4.310
4.303
4.286
4.279
3.796
3.174
3.150
3.126
3.101
2.940
2.914
2.894
2.868
1.333
1.309
1.285
Current Data Parameters
NAME WIN-1037-02
EXPNO 10
PROCNO 1
F2 - Acquisition Parameters
Date_ 20190805
Time 14.21 h
INSTRUM spect
PROBHD Z862701_0064 (
PULPROG zg30
TD 65536
SOLVENT CDCl3
NS 16
DS 2
SWH 6009.615 Hz
FIDRES 0.183399 Hz
AQ 5.4525952 sec
RG 203
DW 83.200 usec
DE 6.50 usec
TE 298.0 K
D1 1.00000000 sec
TD0 1
SFO1 300.1318533 MHz
NUC1 1H
P1 13.00 usec
PLW1 20.00000000 W
F2 - Processing parameters
SI 65536
SF 300.1300072 MHz
WDW EM
SSB 0
LB 0.30 Hz
GB 0
PC 1.00
Ph
N
OMe
F3C
CO2Et
5j
8.5 8.0 7.5 7.0 6.5 6.0 5.5 5.0 4.5 4.0 3.5 3.0 2.5 2.0 1.5 ppm
1.00
2.03
3.22
2.02
2.07
1.02
1.01
2.24
2.99
1.05
1.07
3.41

Current Data Parameters
NAME WIN-1037-02
EXPNO 11
PROCNO 1

F2 - Acquisition Parameters
Date_ 20190805
Time 14.23 h
INSTRUM spect
PROBHD Z862701_0064 (
PULPROG zgflqn
TD 131072
SOLVENT CDCl3
NS 16
DS 4
SWH 66964.289 Hz
FIDRES 1.021794 Hz
AQ 0.9786710 sec
RG 2050
DW 7.467 usec
DE 6.50 usec
TE 298.0 K
D1 1.00000000 sec
TD0 1
SFO1 282.3781148 MHz
NUC1 19F
P1 8.60 usec
PLW1 19.99900055 W

F2 - Processing parameters
SI 65536
SF 282.4043550 MHz
WDW EM
SSB 0
LB 0.30 Hz
GB 0
PC 1.00

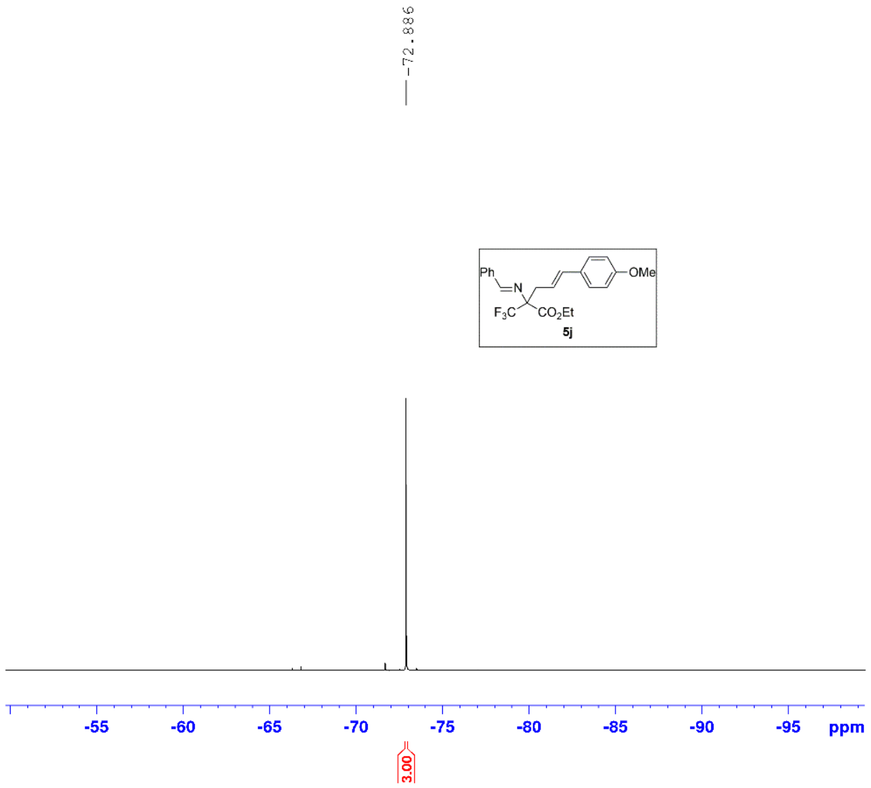

Current Data Parameters
NAME WIN-1037-02
EXPNO 20
PROCNO 1

F2 - Acquisition Parameters
Date_ 20190805
Time 21.18 h
INSTRUM spect
PROBHD Z862701_0064 (
PULPROG zgpg30
TD 65536
SOLVENT CDCl3
NS 2048
DS 4
SWH 18028.846 Hz
FIDRES 0.550197 Hz
AQ 1.8175317 sec
RG 2050
DW 27.733 usec
DE 27.73 usec
TE 298.3 K
D1 2.00000000 sec
D11 0.03000000 sec
TD0 1
SFO1 75.4752949 MHz
NUC1 13C
P1 8.88 usec
PLW1 50.00000000 W
SFO2 300.1312005 MHz
NUC2 1H
CPDPRG[2 waltz16
PCPD2 90.00 usec
PLW2 20.00000000 W
PLW12 0.41727999 W
PLW13 0.20988999 W

F2 - Processing parameters
SI 32768
SF 75.4677392 MHz
WDW EM
SSB 0
LB 1.00 Hz
GB 0
PC 1.40

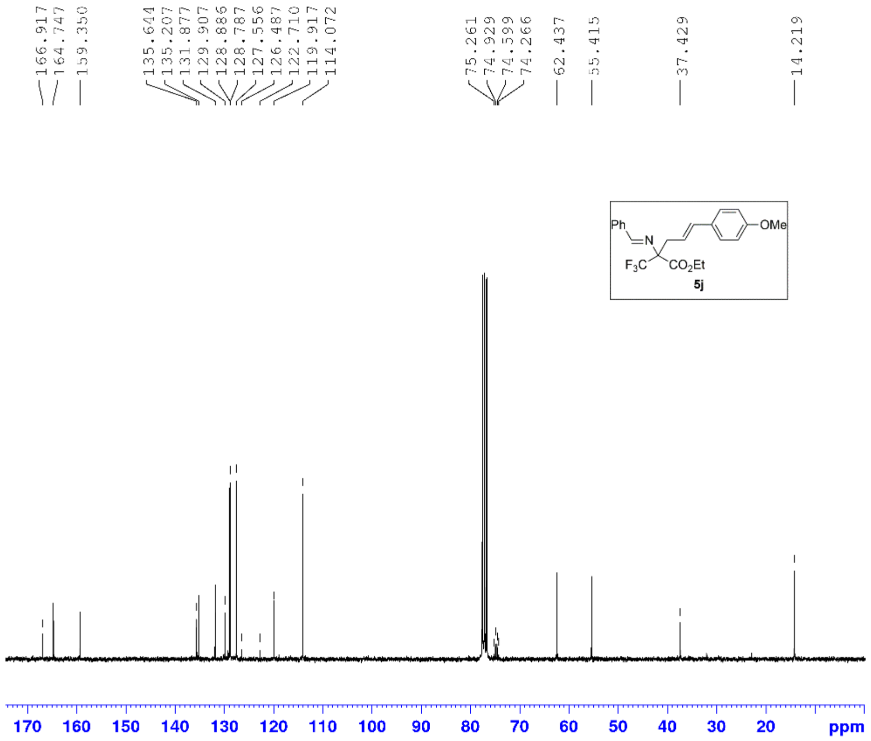

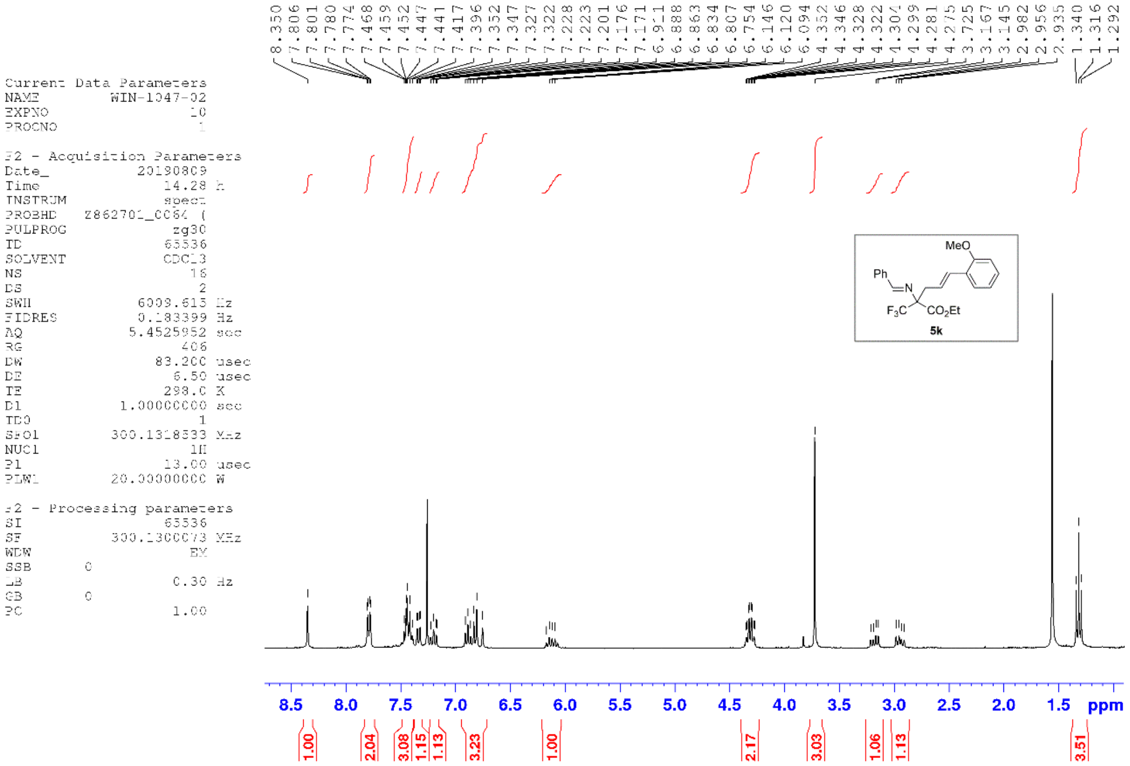
Current Data Parameters
NAME WIN-1047-02
EXPNO 10
PROCNO [illegible]
F2 - Acquisition Parameters
Date_ 20190809
Time 14.28 h
INSTRUM spect
PROBHD Z862701_0064 (
PULPROG zg30
TD 65536
SOLVENT CDCl3
NS 16
DS 2
SWH 6009.615 Hz
FIDRES 0.183399 Hz
AQ 5.4525952 sec
RG 406
DW 83.200 usec
DE 6.50 usec
TE 298.0 K
D1 1.00000000 sec
TD0 1
SFO1 300.1318533 MHz
NUC1 1H
P1 13.00 usec
PLW1 20.00000000 W
F2 - Processing parameters
SI 65536
SF 300.1300073 MHz
WDW EM
SSB 0
LB 0.30 Hz
GB 0
PC 1.00
8.350 7.806 7.801 7.780 7.774 7.468 7.459 7.452 7.447 7.441 7.417 7.396 7.352 7.347 7.327 7.322 7.228 7.223 7.201 7.176 7.171 6.911 6.888 6.863 6.834 6.807 6.754 6.146 6.120 6.094 4.352 4.346 4.328 4.322 4.304 4.299 4.281 4.275 3.725 3.167 3.145 2.982 2.956 2.935 1.340 1.316 1.292
MeO
Ph
N
F3C
CO2Et
5k
8.5 8.0 7.5 7.0 6.5 6.0 5.5 5.0 4.5 4.0 3.5 3.0 2.5 2.0 1.5 ppm
1.00 2.04 3.08 1.15 1.13 3.23 1.00 2.17 3.03 1.06 1.13 3.51

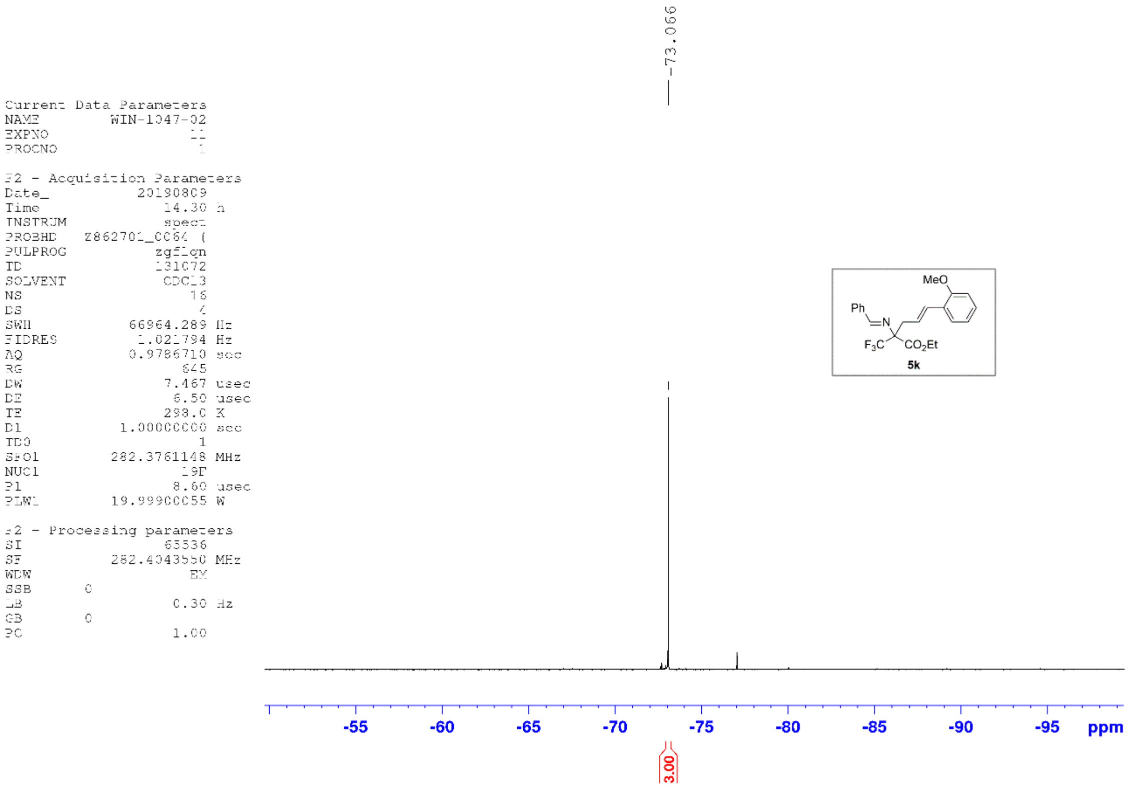
-73.066
Current Data Parameters
NAME WIN-1047-02
EXPNO 11
PROCNO 1
F2 - Acquisition Parameters
Date_ 20190809
Time 14.30 h
INSTRUM spect
PROBHD Z862701_0064 (
PULPROG zgflqn
TD 131072
SOLVENT CDCl3
NS 16
DS 4
SWH 66964.289 Hz
FIDRES 1.021794 Hz
AQ 0.9786710 sec
RG 645
DW 7.467 usec
DE 6.50 usec
TE 298.0 K
D1 1.00000000 sec
TD0 1
SFO1 282.3761148 MHz
NUC1 19F
P1 8.60 usec
PLW1 19.99900055 W
F2 - Processing parameters
SI 65536
SF 282.4043550 MHz
WDW EM
SSB 0
LB 0.30 Hz
GB 0
PC 1.00
MeO
Ph
N
F3C
CO2Et
5k
-55 -60 -65 -70 -75 -80 -85 -90 -95 ppm
3.00

```
Current Data Parameters
NAME           WIN-1047-02
EXPNO                   12
PROCNO                   1

F2 - Acquisition Parameters
Date_             20190810
Time                  8.32 h
INSTRUM              spect
PROBHD   Z862701_0064 (
PULPROG            zgpg30
TD                  65536
SOLVENT             CDCl3
NS                   6144
DS                      4
SWH             18028.846 Hz
FIDRES           0.550197 Hz
AQ              1.8175317 sec
RG                   2050
DW                 27.733 usec
DE                  27.73 usec
TE                  298.0 K
D1             2.00000000 sec
D11            0.03000000 sec
TD0                     1
SFO1           75.4752949 MHz
NUC1                  13C
P1                   8.88 usec
PLW1          50.00000000 W
SFO2          300.1312005 MHz
NUC2                   1H
CPDPRG[2]          waltz16
PCPD2               90.00 usec
PLW2          20.00000000 W
PLW12          0.41727999 W
PLW13          0.20988999 W

F2 - Processing parameters
SI                  32768
SF             75.4677381 MHz
WDW                    EM
SSB       0
LB                   1.00 Hz
GB        0
PC                   1.40
```

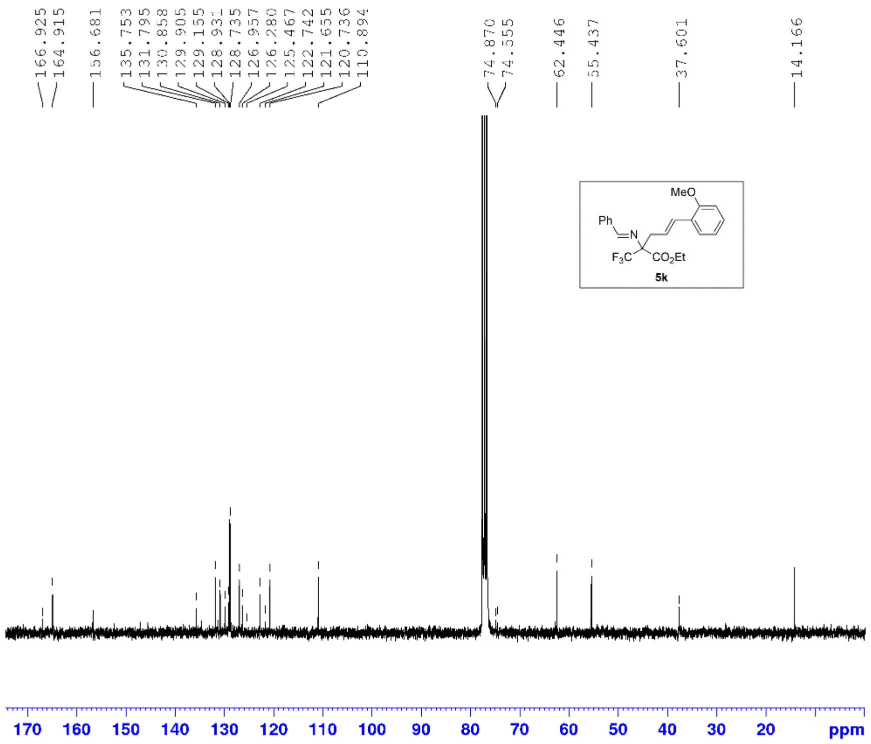


```
Current Data Parameters
NAME           WIN-1039-02
EXPNO                   10
PROCNO                   1

F2 - Acquisition Parameters
Date_             20190803
Time                 14.42 h
INSTRUM              spect
PROBHD   Z862701_0064 (
PULPROG              zg30
TD                  65536
SOLVENT             CDCl3
NS                     16
DS                      2
SWH              6009.615 Hz
FIDRES           0.183399 Hz
AQ              5.4525952 sec
RG                    203
DW                 83.200 usec
DE                   6.50 usec
TE                  298.0 K
D1             1.00000000 sec
TD0                     1
SFO1          300.1318533 MHz
NUC1                   1H
P1                  13.00 usec
PLW1          20.00000000 W

F2 - Processing parameters
SI                  65536
SF            300.1300070 MHz
WDW                    EM
SSB       0
LB                   0.30 Hz
GB        0
PC                   1.00
```

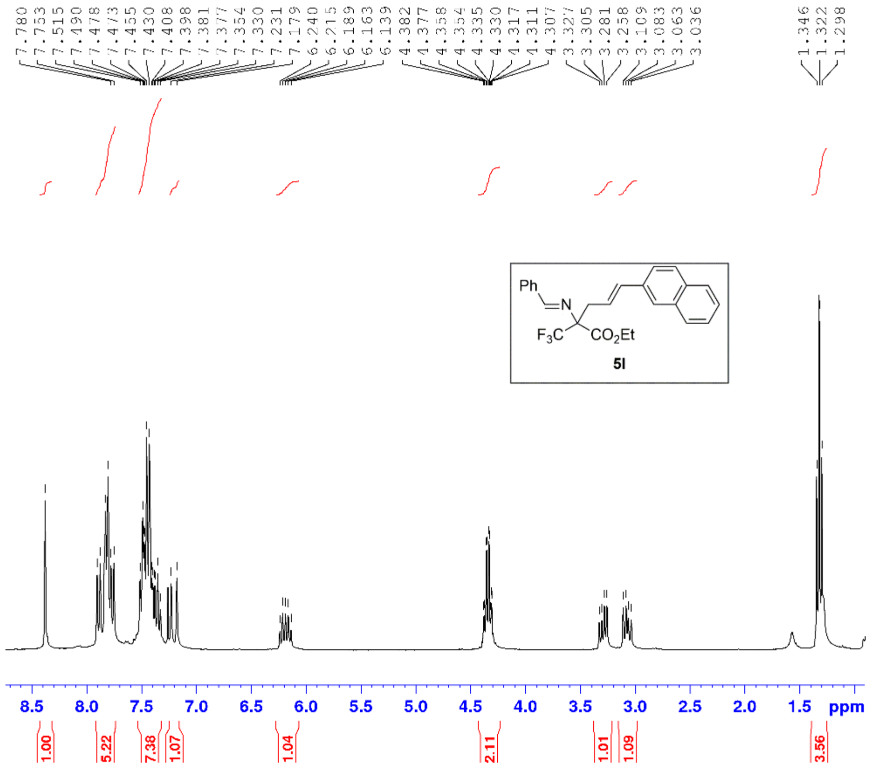

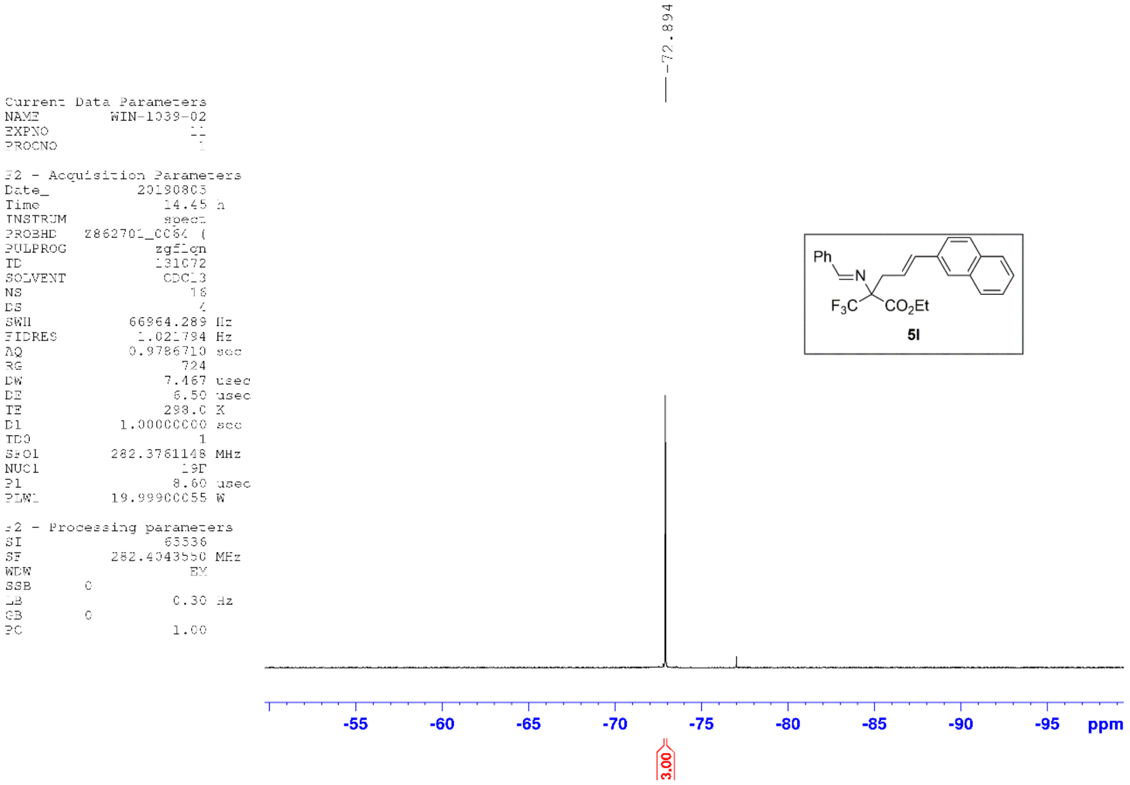

-72.894
Current Data Parameters
NAME WIN-1039-02
EXPNO 11
PROCNO 1
F2 - Acquisition Parameters
Date_ 20190805
Time 14.45 h
INSTRUM spect
PROBHD Z862701_0064 (
PULPROG zgflqn
TD 131072
SOLVENT CDCl3
NS 16
DS 4
SWH 66964.289 Hz
FIDRES 1.021794 Hz
AQ 0.9786710 sec
RG 724
DW 7.467 usec
DE 6.50 usec
TE 298.0 K
D1 1.00000000 sec
TD0 1
SFO1 282.3761148 MHz
NUC1 19F
P1 8.60 usec
PLW1 19.99900055 W
F2 - Processing parameters
SI 65536
SF 282.4043550 MHz
WDW EM
SSB 0
LB 0.30 Hz
GB 0
PC 1.00
Ph
N
F3C
CO2Et
5l
-55
-60
-65
-70
-75
-80
-85
-90
-95
ppm
3.00

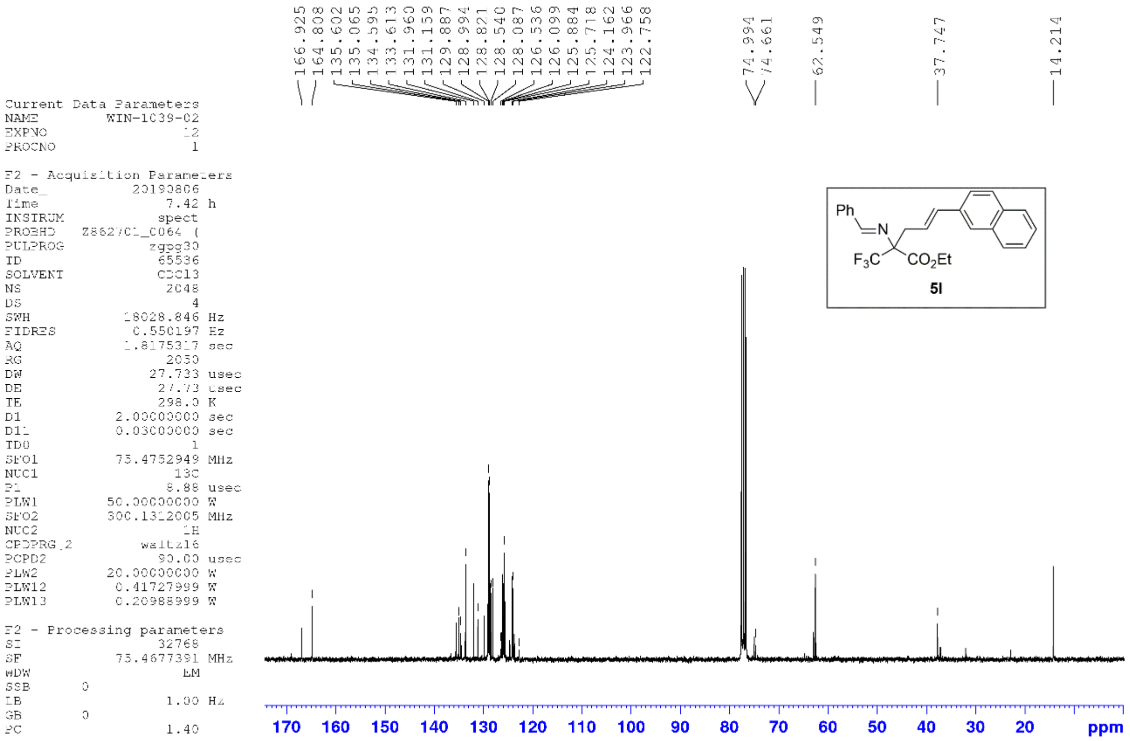

166.925
164.808
135.602
135.065
134.595
133.613
131.960
131.159
129.887
128.994
128.821
128.540
128.087
126.536
126.099
125.884
125.718
124.162
123.966
122.758
74.994
74.661
62.549
37.747
14.214
Current Data Parameters
NAME WIN-1039-02
EXPNO 12
PROCNO 1
F2 - Acquisition Parameters
Date_ 20190806
Time 7.42 h
INSTRUM spect
PROBHD Z862701_0064 (
PULPROG zgpg30
TD 65536
SOLVENT CDCl3
NS 2048
DS 4
SWH 18028.846 Hz
FIDRES 0.550197 Hz
AQ 1.8175317 sec
RG 2050
DW 27.733 usec
DE 27.73 usec
TE 298.3 K
D1 2.00000000 sec
D11 0.03000000 sec
TD0 1
SFO1 75.4752949 MHz
NUC1 13C
P1 8.88 usec
PLW1 50.00000000 W
SFO2 300.1312005 MHz
NUC2 1H
CPDPRG[2 waltz16
PCPD2 90.00 usec
PLW2 20.00000000 W
PLW12 0.41727999 W
PLW13 0.20988999 W
F2 - Processing parameters
SI 32768
SF 75.4677391 MHz
WDW EM
SSB 0
LB 1.00 Hz
GB 0
PC 1.40
Ph
N
F3C
CO2Et
5l
170
160
150
140
130
120
110
100
90
80
70
60
50
40
30
20
ppm

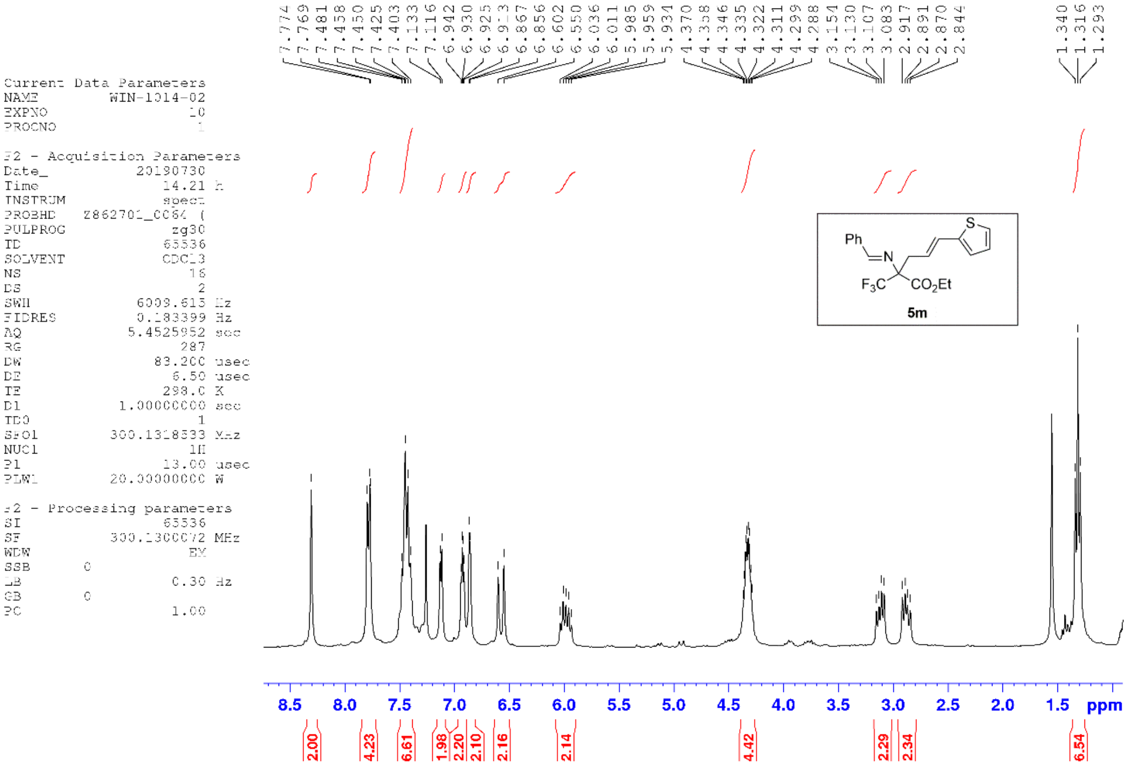
Current Data Parameters
NAME WIN-1014-02
EXPNO 10
PROCNO 1
F2 - Acquisition Parameters
Date_ 20190730
Time 14.21 h
INSTRUM spect
PROBHD Z862701_0064 (
PULPROG zg30
TD 65536
SOLVENT CDCl3
NS 16
DS 2
SWH 6009.615 Hz
FIDRES 0.183399 Hz
AQ 5.4525952 sec
RG 287
DW 83.200 usec
DE 6.50 usec
TE 298.0 K
D1 1.00000000 sec
TD0 1
SFO1 300.1318533 MHz
NUC1 1H
P1 13.00 usec
PLW1 20.00000000 W
F2 - Processing parameters
SI 65536
SF 300.1300072 MHz
WDW EM
SSB 0
LB 0.30 Hz
GB 0
PC 1.00
7.774
7.769
7.481
7.458
7.450
7.425
7.403
7.133
7.116
6.942
6.930
6.925
6.913
6.867
6.856
6.602
6.550
6.036
6.011
5.985
5.959
5.934
4.370
4.358
4.346
4.335
4.322
4.311
4.299
4.288
3.154
3.130
3.107
3.083
2.917
2.891
2.870
2.844
1.340
1.316
1.293
Ph
N
S
F3C
CO2Et
5m
8.5
8.0
7.5
7.0
6.5
6.0
5.5
5.0
4.5
4.0
3.5
3.0
2.5
2.0
1.5
ppm
2.00
4.23
6.61
1.98
2.20
2.10
2.16
2.14
4.42
2.29
2.34
6.54

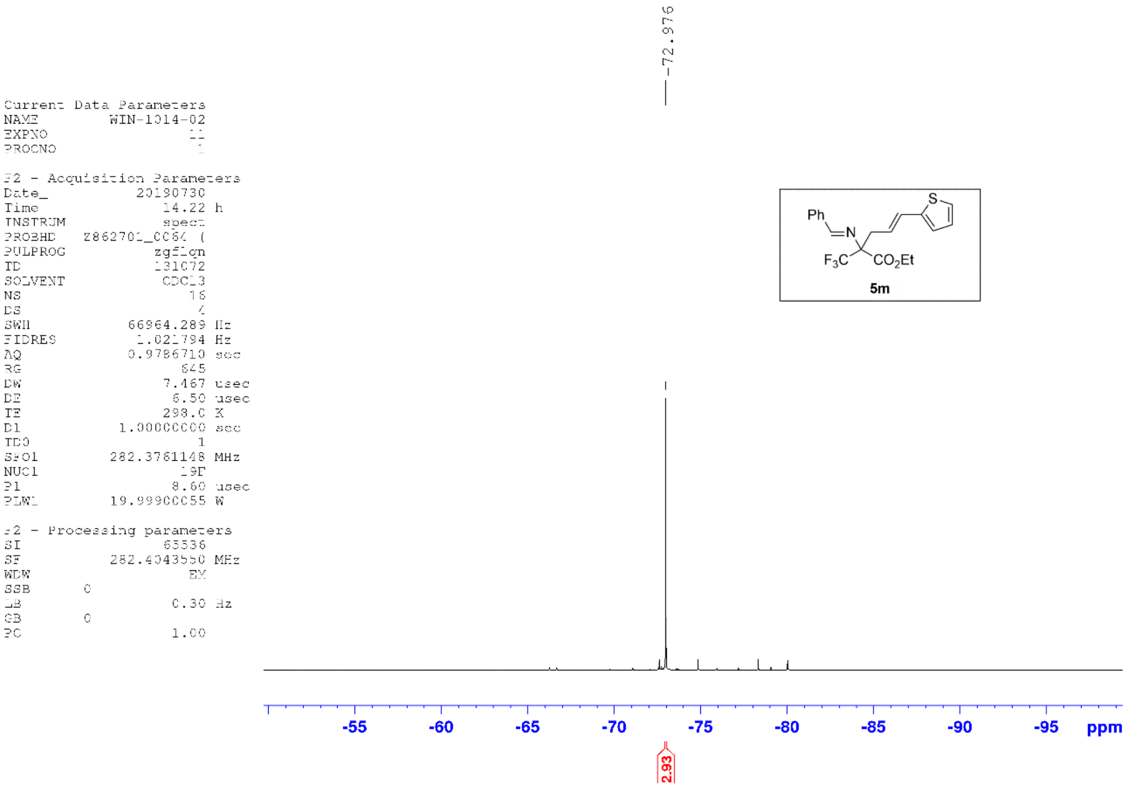
Current Data Parameters
NAME WIN-1014-02
EXPNO 11
PROCNO 1
F2 - Acquisition Parameters
Date_ 20190730
Time 14.22 h
INSTRUM spect
PROBHD Z862701_0064 (
PULPROG zgflqn
TD 131072
SOLVENT CDCl3
NS 16
DS 4
SWH 66964.289 Hz
FIDRES 1.021794 Hz
AQ 0.9786710 sec
RG 645
DW 7.467 usec
DE 6.50 usec
TE 298.0 K
D1 1.00000000 sec
TD0 1
SFO1 282.3761148 MHz
NUC1 19F
P1 8.60 usec
PLW1 19.99900055 W
F2 - Processing parameters
SI 65536
SF 282.4043550 MHz
WDW EM
SSB 0
LB 0.30 Hz
GB 0
PC 1.00
-72.976
Ph
N
S
F3C
CO2Et
5m
-55
-60
-65
-70
-75
-80
-85
-90
-95
ppm
2.93

```
Current Data Parameters
NAME          WIN-1014-02
EXPNO                  12
PROCNO                  1

F2 - Acquisition Parameters
Date_            20190801
Time                 3.30 h
INSTRUM             spect
PROBHD   Z862701_0064 (
PULPROG           zgpg30
TD                 65536
SOLVENT            CDCl3
NS                  2048
DS                     4
SWH            18028.846 Hz
FIDRES          0.550197 Hz
AQ             1.8175317 sec
RG                  2050
DW                27.733 usec
DE                 27.73 usec
TE                 298.0 K
D1            2.00000000 sec
D11           0.03000000 sec
TD0                    1
SFO1          75.4752949 MHz
NUC1                 13C
P1                  8.88 usec
PLW1         50.00000000 W
SFO2         300.1312005 MHz
NUC2                  1H
CPDPRG_2         waltz16
PCPD2              90.00 usec
PLW2         20.00000000 W
PLW12         0.41727999 W
PLW13         0.20988999 W

F2 - Processing parameters
SI                 32768
SF            75.4677384 MHz
WDW                   EM
SSB       0
LB                  1.00 Hz
GB        0
PC                  1.40
```

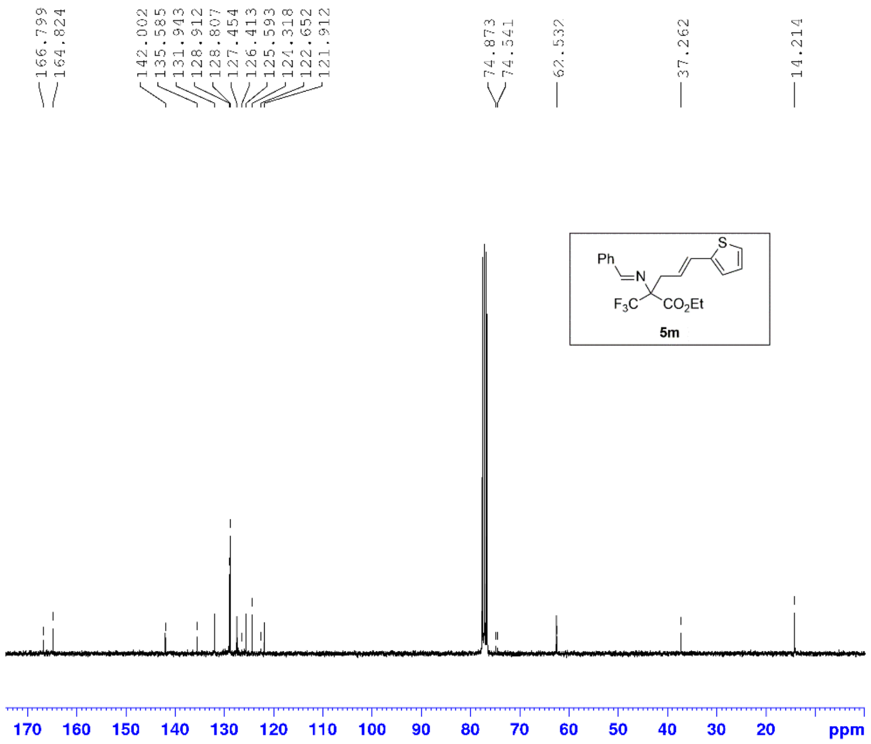


```
Current Data Parameters
NAME          WIN-672-03
EXPNO                 10
PROCNO                 1

F2 - Acquisition Parameters
Date_           20190117
Time               10.48 h
INSTRUM            spect
PROBHD   Z862701_0064 (
PULPROG             zg30
TD                 65536
SOLVENT            CDCl3
NS                    16
DS                     2
SWH             6009.615 Hz
FIDRES          0.183399 Hz
AQ             5.4525952 sec
RG                   456
DW                83.200 usec
DE                  6.50 usec
TE                 298.0 K
D1            1.00000000 sec
TD0                    1
SFO1         300.1318333 MHz
NUC1                  1H
P1                 13.00 usec
PLW1         20.00000000 W

F2 - Processing parameters
SI                 65536
SF           300.1300073 MHz
WDW                   EM
SSB       0
LB                  0.30 Hz
GB        0
PC                  1.00
```

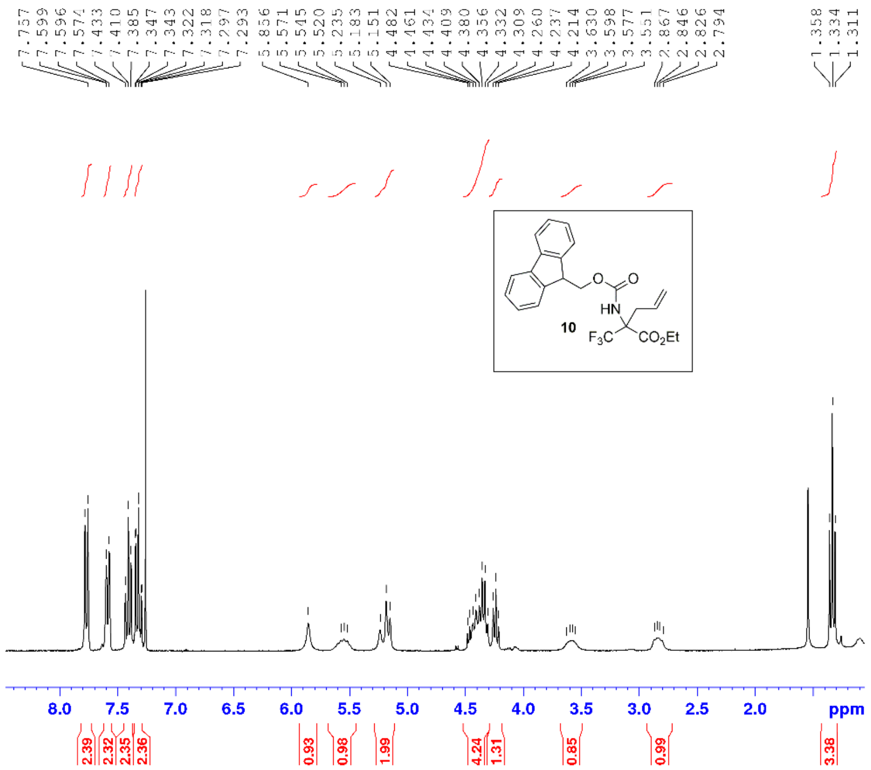

```
Current Data Parameters
NAME          WIN-672-03
EXPNO                 11
PROCNO                 1

F2 - Acquisition Parameters
Date_           20190117
Time               10.50 h
INSTRUM            spect
PROBHD   Z862701_0064 (
PULPROG           zgflqn
TD                131072
SOLVENT            CDCl3
NS                    16
DS                     4
SWH            66964.289 Hz
FIDRES          1.021794 Hz
AQ             0.9786710 sec
RG                   645
DW                 7.467 usec
DE                  6.50 usec
TE                 298.0 K
D1            1.00000000 sec
TD0                    1
SFO1         282.3761148 MHz
NUC1                 19F
P1                  8.60 usec
PLW1         19.99900055 W

F2 - Processing parameters
SI                 65536
SF           282.4043560 MHz
WDW                   EM
SSB      0
LB                  0.30 Hz
GB       0
PC                  1.00
```

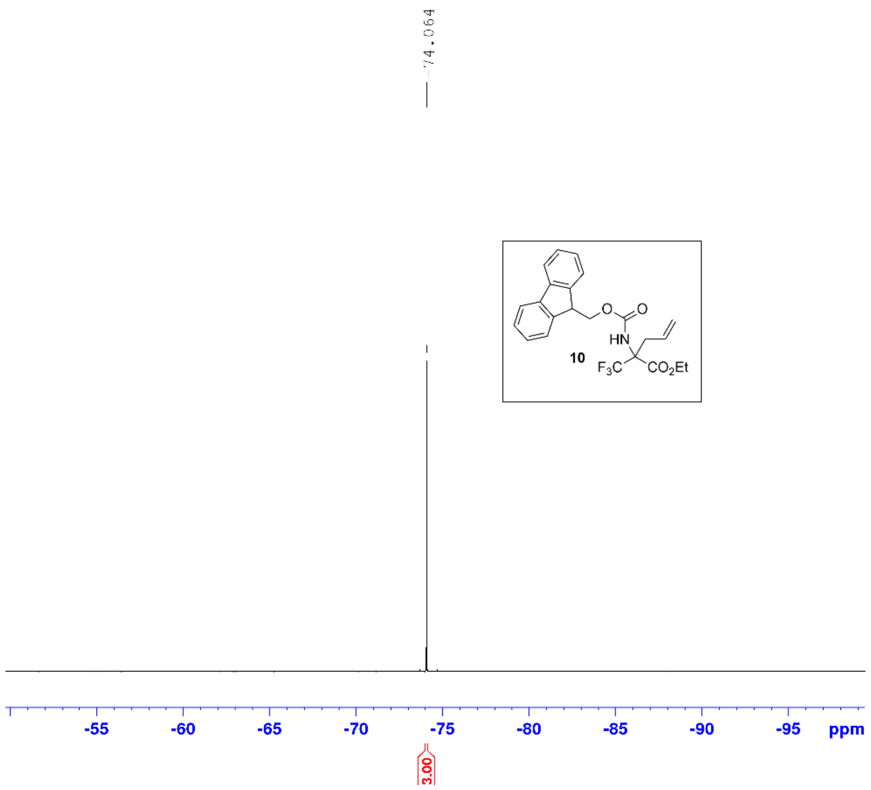


```
Current Data Parameters
NAME         WIN-1127-02
EXPNO                 10
PROCNO                 1

F2 - Acquisition Parameters
Date_           20191004
Time               12.44 h
INSTRUM            spect
PROBHD   Z862701_0064 (
PULPROG             zg30
TD                 65536
SOLVENT            CDCl3
NS                    16
DS                     2
SWH             6009.615 Hz
FIDRES          0.183399 Hz
AQ             5.4525952 sec
RG                   512
DW                83.200 usec
DE                  6.50 usec
TE                 298.0 K
D1            1.00000000 sec
TD0                    1
SFO1         300.1318533 MHz
NUC1                  1H
P1                 13.00 usec
PLW1         20.00000000 W

F2 - Processing parameters
SI                 65536
SF           300.1300072 MHz
WDW                   EM
SSB      0
LB                  0.30 Hz
GB       0
PC                  1.00
```

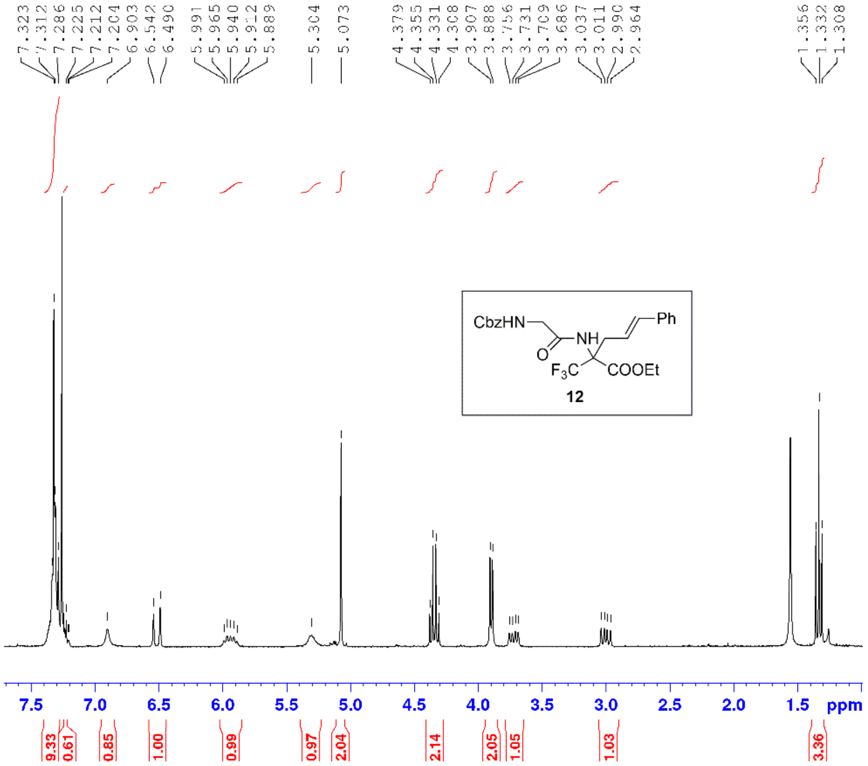

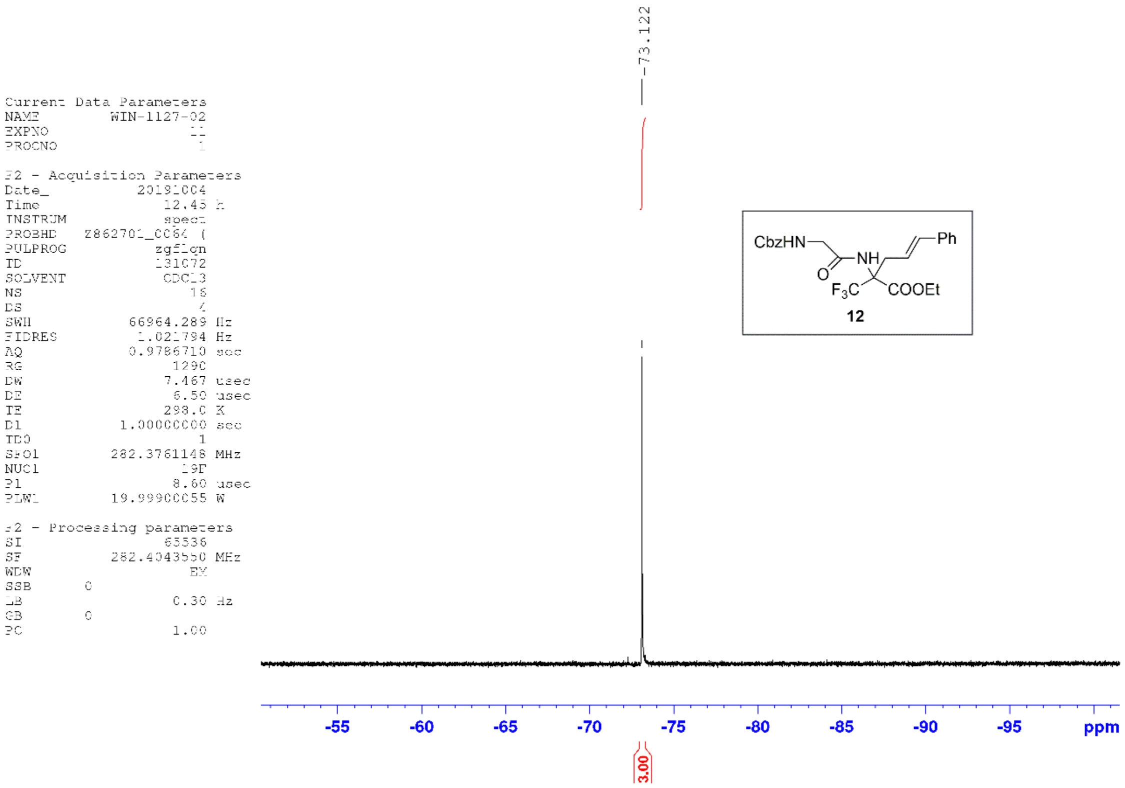
-73.122
Current Data Parameters
NAME WIN-1127-02
EXPNO 11
PROCNO 1
F2 - Acquisition Parameters
Date_ 20191004
Time 12.45 h
INSTRUM spect
PROBHD Z862701_0064 (
PULPROG zgflqn
TD 131072
SOLVENT CDCl3
NS 16
DS 4
SWH 66964.289 Hz
FIDRES 1.021794 Hz
AQ 0.9786710 sec
RG 1290
DW 7.467 usec
DE 6.50 usec
TE 298.0 K
D1 1.00000000 sec
TD0 1
SFO1 282.3761148 MHz
NUC1 19F
P1 8.60 usec
PLW1 19.99900055 W
F2 - Processing parameters
SI 65536
SF 282.4043550 MHz
WDW EM
SSB 0
LB 0.30 Hz
GB 0
PC 1.00
CbzHN
NH
Ph
O
F3C
COOEt
12
-55
-60
-65
-70
-75
-80
-85
-90
-95
ppm
3.00

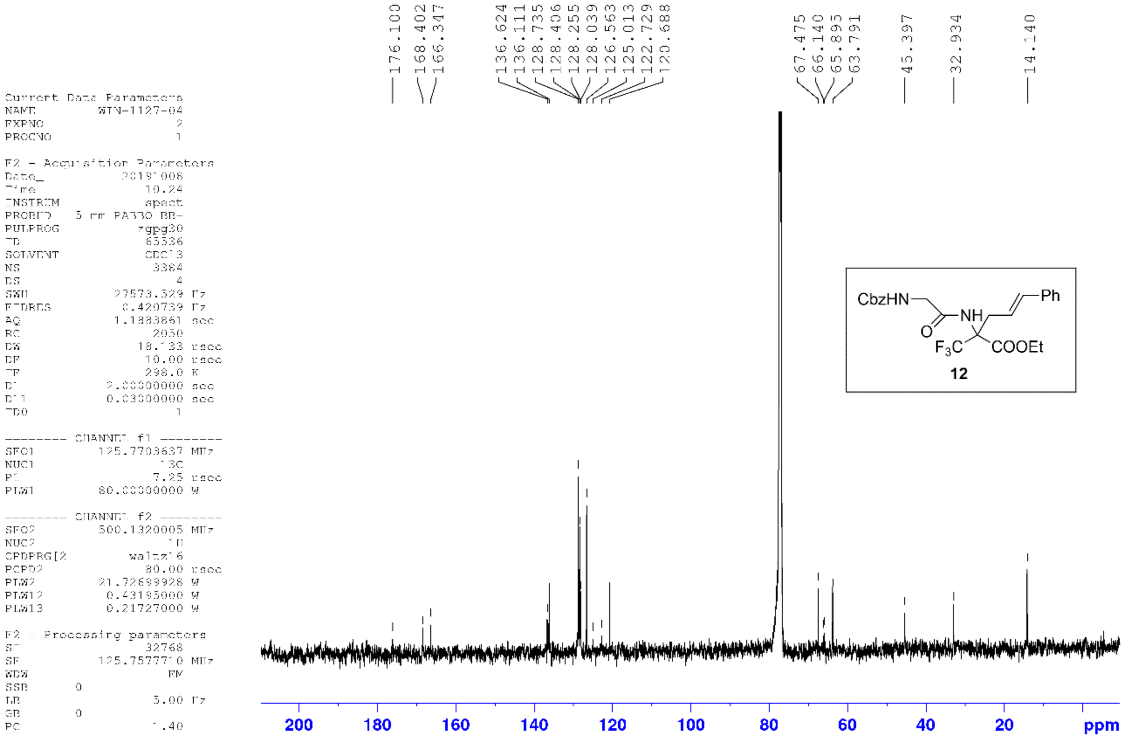
176.100
168.402
166.347
136.624
136.111
128.735
128.406
128.255
128.039
126.563
125.013
122.729
120.688
67.475
66.140
65.895
63.791
45.397
32.934
14.140
Current Data Parameters
NAME WIN-1127-04
EXPNO 2
PROCNO 1
F2 - Acquisition Parameters
Date_
Time 10.24
INSTRUM spect
PROBHD 5 mm PA330 BB-
PULPROG zgpg30
TD 65536
SOLVENT CDCl3
NS 3384
DS 4
SWH 27573.529 Hz
AQ 1.1883861 sec
RG 2050
DE 10.00 usec
TE 298.0 K
TD0 1
CHANNEL f1
SFO1 125.7703637 MHz
NUC1 13C
P1 7.25 usec
PLW1 80.00000000 W
CHANNEL f2
SFO2 500.1320005 MHz
NUC2 1H
CPDPRG[2 waltz16
PCPD2 80.00 usec
PLW2 21.72699928 W
PLW12 0.43195000 W
PLW13 0.21727000 W
F2 - Processing parameters
SI 32768
SF 125.7577710 MHz
WDW EM
SSB 0
LB 3.00 Hz
GB 0
PC 1.40
CbzHN
NH
Ph
O
F3C
COOEt
12
200
180
160
140
120
100
80
60
40
20
ppm

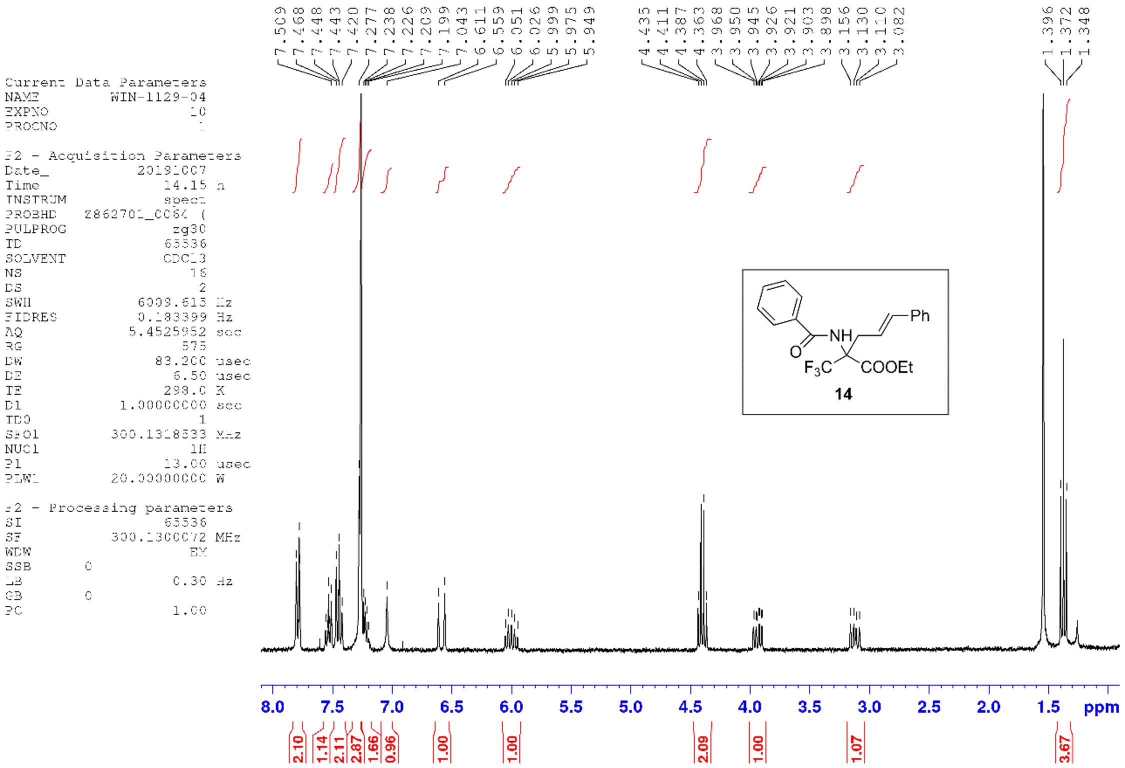
7.509 7.468 7.448 7.443 7.420 7.277 7.238 7.226 7.209 7.199 7.043 6.611 6.559 6.051 6.026 5.999 5.975 5.949
4.435 4.411 4.387 4.363 3.968 3.950 3.945 3.926 3.921 3.903 3.898 3.156 3.130 3.110 3.082
1.396 1.372 1.348
Current Data Parameters
NAME WIN-1129-04
EXPNO 10
PROCNO 1
F2 - Acquisition Parameters
Date_ 20191007
Time 14.15 h
INSTRUM spect
PROBHD Z862701_0064 (
PULPROG zg30
TD 65536
SOLVENT CDCl3
NS 16
DS 2
SWH 6009.615 Hz
FIDRES 0.183399 Hz
AQ 5.4525952 sec
RG 575
DW 83.200 usec
DE 6.50 usec
TE 298.0 K
D1 1.00000000 sec
TD0 1
SFO1 300.1318533 MHz
NUC1 1H
P1 13.00 usec
PLW1 20.00000000 W
F2 - Processing parameters
SI 65536
SF 300.1300072 MHz
WDW EM
SSB 0
LB 0.30 Hz
GB 0
PC 1.00
O
NH
Ph
F3C
COOEt
14
8.0 7.5 7.0 6.5 6.0 5.5 5.0 4.5 4.0 3.5 3.0 2.5 2.0 1.5 ppm
2.10 1.14 2.11 2.87 1.66 0.96 1.00 1.00 2.09 1.00 1.07 3.67

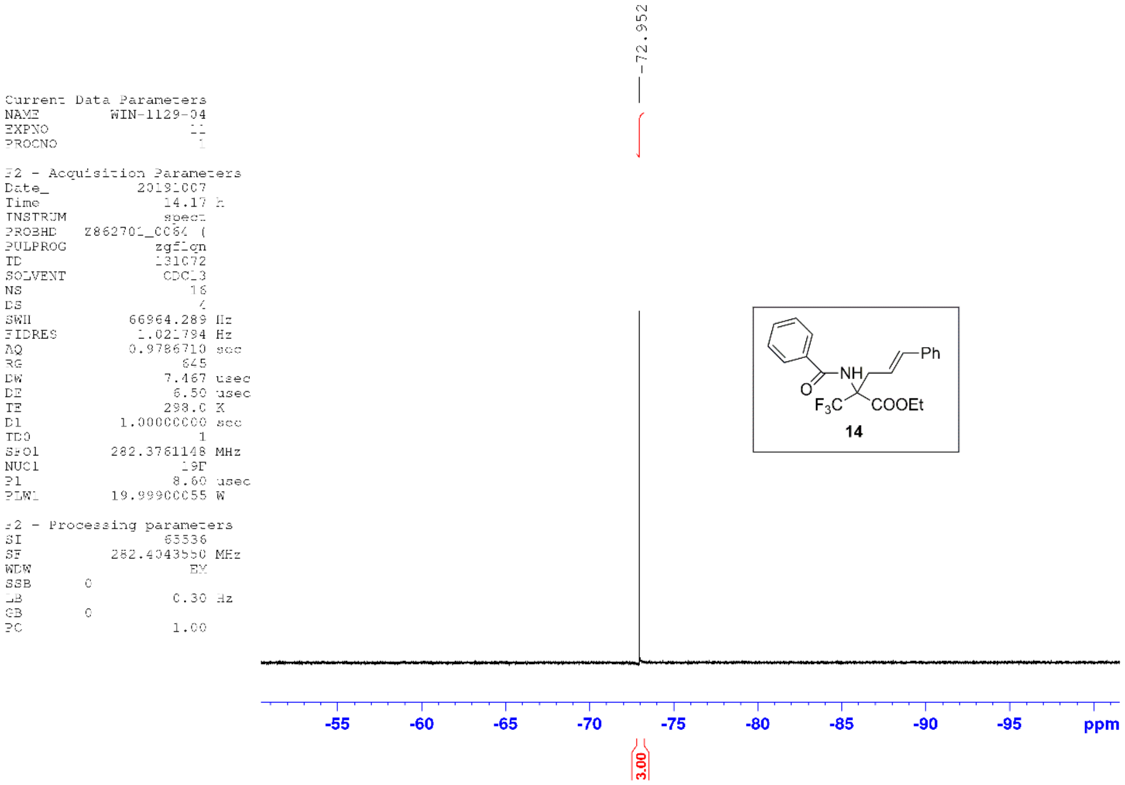
-72.952
Current Data Parameters
NAME WIN-1129-04
EXPNO 11
PROCNO 1
F2 - Acquisition Parameters
Date_ 20191007
Time 14.17 h
INSTRUM spect
PROBHD Z862701_0064 (
PULPROG zgflqn
TD 131072
SOLVENT CDCl3
NS 16
DS 4
SWH 66964.289 Hz
FIDRES 1.021794 Hz
AQ 0.9786710 sec
RG 645
DW 7.467 usec
DE 6.50 usec
TE 298.0 K
D1 1.00000000 sec
TD0 1
SFO1 282.3761148 MHz
NUC1 19F
P1 8.60 usec
PLW1 19.99900055 W
F2 - Processing parameters
SI 65536
SF 282.4043550 MHz
WDW EM
SSB 0
LB 0.30 Hz
GB 0
PC 1.00
O
NH
Ph
F3C
COOEt
14
-55 -60 -65 -70 -75 -80 -85 -90 -95 ppm
3.00
